# Fear learning–induced brain dynamics predict individual extinction memory expression following transcranial magnetic stimulation

**DOI:** 10.1038/s41467-026-78345-8

**Published:** 2026-10-05

**Authors:** Kai Zhang, Lihan Cui, B.Isabel Moallem, Hani Meelad, Zakiya Atiyah, Muhammad Badarnee, Isabella Mason, Zhenfu Wen, Mark S. George, Mohammed R. Milad

**Affiliations:** 1 Department of Psychiatry and Behavioral Sciences, University of Texas Health Science Center at Houston, Houston, TX USA; 2 Department of Psychiatry and Behavioral Sciences, Medical University of South Carolina, Charleston, SC USA; 3 Ralph H. Johnson VA Medical Center, Charleston, SC USA

**Keywords:** Extinction, Fear conditioning

## Abstract

Fear learning and extinction unfold as time-dependent processes. Herein, we examined whether fear learning dynamically reorganizes brain activity and whether such reorganization predicts subsequent extinction-memory expression under transcranial magnetic stimulation (TMS)-modulated extinction. Eighty-seven healthy adults completed a three-day Pavlovian threat-learning protocol with resting-state fMRI, TMS-modulated extinction, and extinction-memory fMRI. Using coactivation pattern analysis with a hidden Markov model, we identified a fear-learning–induced brain state characterized by increased engagement and transition uncertainty after conditioning. Functional connectivity reorganization within this state predicted individual differences in extinction recall- and renewal-related brain activation under TMS-modulated extinction (cross-validated; recall *r* = 0.47, *p* = 0.001; renewal *r* = 0.37, *p* = 0.01; permutation-tested), but not natural extinction. In separate brain–behavior correlation analyses, the neural variates identified in the TMS-modulated extinction were associated with behavioral expression. These findings demonstrate that fear learning reshapes brain-state dynamics and identifies a candidate biomarker of neuromodulation-linked extinction-memory expression.

## Abstract (web summary)

Zhang et al. show that fear learning reshapes spontaneous brain activity and that these changes can predict individual differences in how extinction memories are later expressed following TMS-modulated extinction.

## Introduction

Understanding the underlying neural mechanisms of how conditioned threat memories are generated, consolidated, and updated is central to explaining the neurobiology of human defensive behavior, and to informing interventions for anxiety- and trauma-related disorders^1–3^. Extensive neuroimaging research has identified the large-scale neural circuitry supporting fear conditioning and extinction, including the amygdala, hippocampus, insula, anterior cingulate cortex, and ventromedial prefrontal cortex (vmPFC)^4–10^. Additionally, converging evidence from both animal and human studies suggests that fear learning is inherently dynamic. In rodent models, the engagement of key fear-learning structures, including the amygdala, hippocampus, and vmPFC, varies across different stages of acquisition, consolidation, and extinction^11–18^. Post-learning consolidation processes emerge immediately following learning and continue to evolve over time^19^. Consistent with these observations, human neuroimaging studies have demonstrated that fear conditioning induces measurable reorganization of spontaneous brain activity during post-learning. Using both seed-based and data-driven approaches, prior work has shown that fear conditioning alters resting-state functional connectivity within amygdala-prefrontal, amygdala-hippocampal, and default-mode network systems^20,21^, that these changes can persist across consolidation periods ranging from hours to days, and that they are associated with subsequent fear expression, extinction efficacy, and individual differences in anxiety-related traits^22–25^. Collectively, these findings suggest that fear learning leaves a measurable and temporally evolving trace on large-scale brain organization that extends beyond the learning episode itself and contributes to subsequent memory expression.

Despite these advances, prior work has largely characterized post-learning brain reorganization at the level of regional connectivity or network interactions averaged across entire scans. While such approaches provide important information regarding average patterns of functional organization, they offer limited insight into the moment-to-moment evolution of spontaneous brain activity. Comparatively less is known about whether fear learning reorganizes spontaneous activity at the level of recurring transient brain states, including how frequently such states occur, how long they persist, and how they transition over time. Consistent with a broader shift in systems neuroscience, recent frameworks are increasingly moving beyond static descriptions toward dynamic models that characterize cognition and learning in terms of transitions -through time during and after learning- among a set of recurrent brain states^26–29^. For example, Sendi et al. demonstrated that alterations in the engagement and stability of specific brain states are associated with subsequent symptom expression following trauma exposure, highlighting the clinical relevance of dynamic state organization in the early aftermath of learning-related stress^30^. Longitudinal work from Awada et al. further showed that reproducible coactivation patterns constitute stable brain-state signatures linked to motivational and affective disturbances in psychiatric populations^31^. Complementarily, clinical trial research from Cai et al. revealed that treatment response in major depressive disorder and bipolar disorder is reflected in reorganization of brain-state engagement and transition probabilities^32^. These findings illustrate that characterizing state-to-state switching dynamics provides a principled framework for interrogating how cognitive processes and neuromodulation reshape functional brain organization over time.

However, the specific contribution of transient brain-state organization to fear-learning consolidation and subsequent memory expression remains insufficiently understood. In particular, it remains unknown whether fear-learning–induced reorganization of spontaneous brain-state architecture can prospectively predict subsequent extinction-memory expression and neuromodulation efficacy.

Against this background, the present work addresses two important and unresolved questions. First, how fear conditioning dynamically reconfigures the engagement, persistence, and switching structure of spontaneous brain states immediately after learning. Second, whether learning-induced brain-state reorganization predicts subsequent extinction-memory expression, and whether this relationship differs between neuromodulation-enhanced and standard extinction learning procedures. Within this process, we leveraged transcranial magnetic stimulation (TMS) as a mechanistically motivated neuromodulation approach targeting dorsolateral prefrontal cortex (DLPFC)—designed to indirectly modulate ventromedial prefrontal cortex (vmPFC) function through connectivity-guided targeting—to facilitate extinction memory, consistent with prior rodent and human studies^1,33–38^. Yet, it remains unresolved whether fear-learning–induced brain-state reorganization can be leveraged as an individual-level neural marker for prospectively predicting extinction-memory expression, and whether the predictive relevance of such reorganization depends on neuromodulation intervention versus natural extinction.

**Fig. 1:**
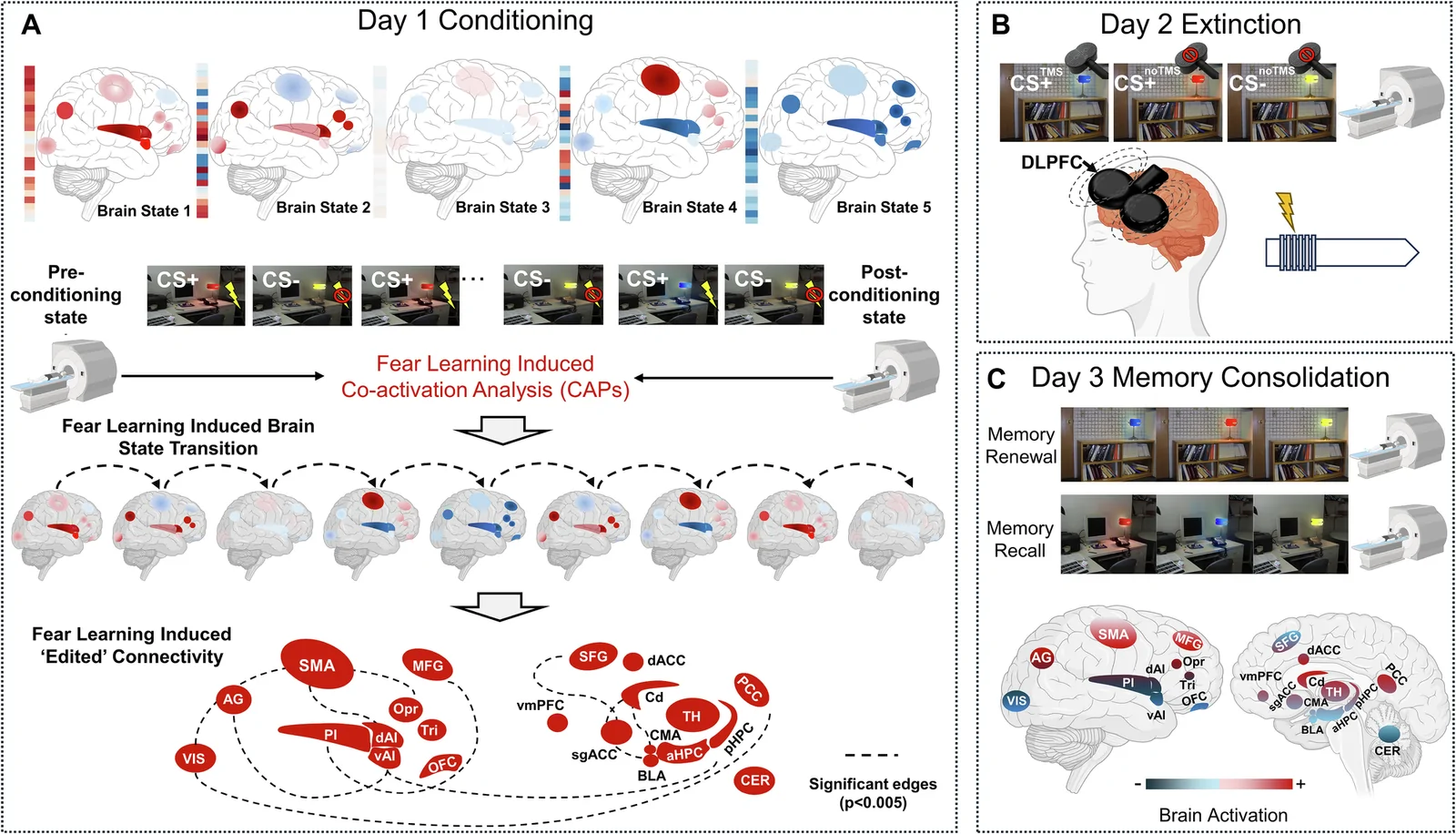
Conceptual and Analytical framework for tracking fear-learning–induced dynamic neural mechanism. Participants completed a three-day experimental protocol (Milad fear learning paradigm) with fear conditioning and extinction. **A** On Day 1, participants underwent a fear conditioning paradigm, with resting-state fMRI acquired before (PRE) and after (POST) conditioning to capture learning-induced changes in spontaneous brain activity. Coactivation pattern (CAP) analysis was applied to the combined PRE- and POST-conditioning resting-state fMRI data. The five numbered brain states shown at the top of (**A**) represent recurrent coactivation patterns identified by the hidden Markov model from the combined resting-state scans. The schematic immediately below the conditioning paradigm illustrates representative transitions among these recurrent brain states during resting-state fMRI, and changes in state engagement and transition dynamics before versus after conditioning were used to quantify fear-learning–induced brain-state reorganization. At the bottom of (**A**), functional connectivity was estimated within the identified fear-learning–related brain state to characterize conditioning-induced connectivity reorganization, with only significant connectivity changes illustrated schematically. **B** On Day 2, participants received TMS-modulated extinction targeting the dorsolateral prefrontal cortex (DLPFC). **C** On Day 3, task for memory recall and fear renewal. Brain activation was used to evaluate extinction memory consolidation. BLA basolateral amygdala, CMA centromedial amygdala, aHPC anterior hippocampus, pHPC posterior hippocampus, dAI dorsal anterior insula, vAI ventral anterior insula, PI posterior insular cortex, sgACC subgenual anterior cingulate cortex, dACC dorsal anterior cingulate cortex, vmPFC ventromedial prefrontal cortex, SFG superior frontal gyrus, MFG middle frontal gyrus, Tri (IFtri) triangular part of inferior frontal gyrus, Opr (IFGoper) opercular part of the inferior frontal gyrus, Precentral precentral gyrus, Postcentral Postcentral gyrus, SMA supplementary motor area, AG angular gyrus, PCC posterior cingulate cortex, VIS visual cortex, TH thalamus, Cd caudate nucleus, OFC Orbital frontal cortex, CER cerebellum). Icons were created in BioRender, with attribution lines: *Zhang, K. (2026)* https://BioRender.com/d8rx7ph*, Zhang, K. (2026)* https://BioRender.com/6edw6d9*, and Zhang, K. (2026)* https://BioRender.com/ku1s189.

To begin to fill in the gap, we implemented a three-day Pavlovian fear-learning and extinction protocol combined with neuromodulation and data-driven computational modeling (Fig. 1). On Day 1, 87 healthy participants (43 males and 44 females; mean age = 28.79 ± 9.20 years) completed a Pavlovian fear-conditioning paradigm, in which conditioned threat associations were learned by pairing visual cues of distinct colors (CS + ) with aversive electric shocks, while other cues (CS − ) were never reinforced. On Day 2, participants underwent extinction learning under transcranial magnetic stimulation (TMS) targeting the dorsolateral prefrontal cortex (DLPFC), enabling experimental modulation of regulatory processes during fear extinction. On Day 3, participants completed memory recall and renewal tasks to probe the expression of conditioned threat memories following extinction and neuromodulation. We hypothesized that fear learning would induce systematic reorganization of brain-state dynamics and that such learning-induced neural reorganization would contain information relevant to subsequent extinction-memory expression. To test this possibility, we quantified fear-learning–induced changes in spontaneous brain dynamics from pre- to post-conditioning and examined whether these changes predicted subsequent memory expression under TMS-modulated extinction and natural extinction. We further evaluated the behavioral relevance of these neural signatures by examining their relationship to individual differences in fear expression. These findings provide insight into the neural mechanisms of fear-learning–induced brain-state reorganization and demonstrate that such learning-induced brain-state reorganization may serve as a prospective neural biomarker linking fear acquisition to subsequent memory expression. An illustration of our conceptual and analytic framework is shown in Fig. 1.

## Results

### Fear-learning induced Brain State and its Dynamics Indices

Successful fear acquisition was confirmed by behavioral and psychophysiological measures (Suppl. Fig. 1). We employed a data-driven approach based on a Hidden Markov Model (HMM) to perform coactivation pattern (CAP) analysis. Resting-state fMRI data acquired before and after fear conditioning, defined within an extended 24-node threat-circuit parcellation^18^, were analyzed to characterize the dominant modes of spontaneous brain activity at the population level. Paired t-tests were then used to compare CAP-derived dynamic indicators between pre- and post-conditioning, enabling identification of the dominant brain state modulated by fear learning.

**Fig. 2:**
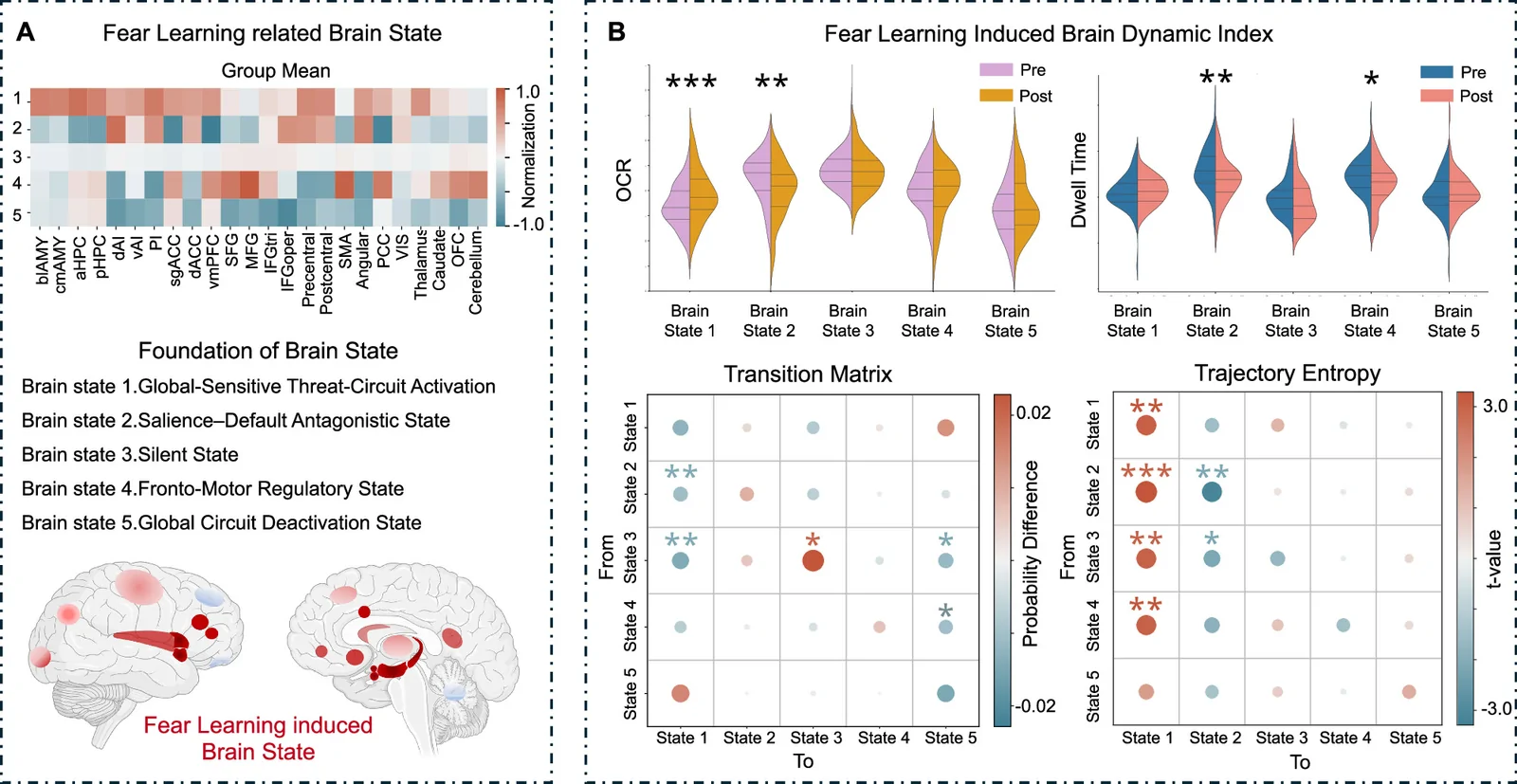
Identification of fear-learning–related dynamic brain states using coactivation pattern (CAP) analysis. **A** Definition of a fear-learning–related brain state based on convergent changes across the four dynamic metrics. The corresponding spatial coactivation pattern is shown within the extended 24-node threat-circuit mask. **B** CAP-derived dynamic metrics characterizing fear-learning–induced brain-state reorganization during Day 1 resting-state fMRI. Four indices were quantified, including occurrence rate (OCR), dwell time, state transition probability matrix, and trajectory entropy, capturing the temporal prevalence, persistence, transition structure, and dynamical complexity of coactivation patterns before and after fear conditioning. Statistical significance was assessed using two-sided paired t-tests. *P*-values were adjusted for multiple comparisons using the Benjamini–Hochberg false discovery rate (FDR) procedure within each dynamic metric. Data were obtained from 80 independent healthy participants (*n* = 80). Asterisks indicate FDR-adjusted significance levels (*p < 0.05, **p < 0.01, *p < 0.001). Exact FDR-adjusted *p*-values for significant comparisons were: OCR, State 1, *p* = 0.0002 and State 2, *p* = 0.0011. Dwell time, State 2, *p* = 0.0060 and State 4, *p* = 0.0365. Transition probability, State 2 → 1, *p* = 0.0061, State 3 → 1, *p* = 0.0024, State 3 → 3, *p* = 0.0132, State 3 → 5, *p* = .0115, and State 4 → 5, *p* = 0.0350. Trajectory entropy, State 1 → 1, *p* = 0.0028, State 2 → 1, *p* = 0.0008, State 2 → 2, *p* = 0.0029, State 3 → 1, *p* = 0.0032, State 3 → 2, *p* = 0.0351, and State 4 → 1, *p* = 0.0033. Icons were created in BioRender, with attribution lines: *Zhang, K. (2026)* https://BioRender.com/d8rx7ph.

Model-selection analyses supported a five-state solution (Suppl. Fig. 2). Five distinct recurring coactivation patterns were defined as basic brain states across all participants. As shown in Fig. 2A, these states were distinguished by their spatial activation profiles and were labeled based on their predominant circuit engagement (Suppl. Fig. 3). (1) Conditioning-sensitive global circuit activation state, characterized by coordinated positive activation across the majority of regions and conditioning-related increases across multiple dynamic metrics following fear conditioning; (2) Salience–default mode antagonistic state, marked by elevated dAI (dorsal anterior insula), Angular activity with suppression of vmPFC (ventromedial prefrontal cortex), sgACC (subgenual anterior cingulate cortex), and PCC (posterior cingulate cortex); (3) Silent state, exhibiting minimal activation across regions; (4) Fronto-motor regulatory state, dominated by MFG (middle frontal gyrus), SMA (supplementary motor area), and PCC (posterior cingulate cortex) activation with concurrent suppression of insular regions and (5) Global circuit deactivation state, reflecting widespread attenuation across the circuit.

We next examined how fear learning reshaped the temporal dynamics of these states using four complementary dynamic metrics: occurrence rate (OCR) —how often states are engaged, mean dwell time (DT)—how long they are sustained, state transition probability—how they switch, and trajectory entropy—how uncertainty or flexibility the switching process is (Fig. 2B). Among the five states, the conditioning-sensitive global circuit activation state (State 1) showed a selective and consistent modulation following fear conditioning (Suppl. Fig. 4). Specifically, this state exhibited an increase in occurrence rate (*p*_FDR_ < 0.05) from pre- to post-conditioning, indicating more frequent engagement after learning. Analysis of the transition probability matrix revealed a biased reorganization of state transitions toward state 1, with increased transitions from States 2 and 3 into State 1 (*p*_FDR_ < 0.05) following conditioning. In parallel, trajectory entropy analyses demonstrated a selective increase in transition entropy involving State 1, reflecting enhanced flexibility and diversification of state-to-state trajectories converging on this fear-related configuration. Notably, entropy increases were observed for transitions from States 1–4 toward State 1 (*p*_FDR_ < 0.05), whereas no comparable changes were detected for other states. These effects remained robust after accounting for demographic variability, with neither age nor sex significantly associated with the observed state-level changes (Suppl. Information).

### Temporal dynamics of the fear-learning–induced brain state after conditioning

**Fig. 3:**
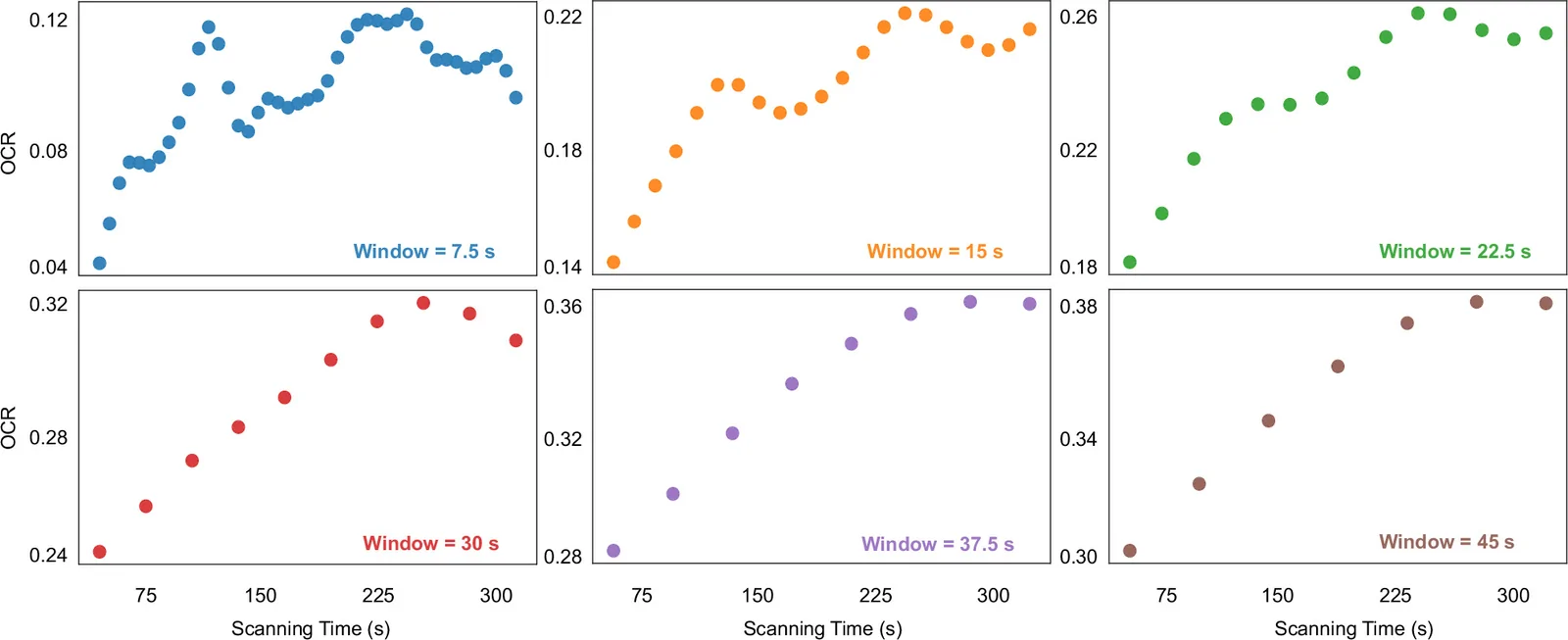
Temporal tracking of fear-learning–induced brain-state engagement during post-conditioning rest. Post-conditioning resting-state fMRI data were segmented using sliding temporal windows of varying lengths. For each window, the occurrence rate of the identified the brain state was computed, indexing its relative engagement over time. The six panels depict OCR trajectories across six window lengths (temporal resolutions), spanning coarse to fine scales. Across temporal resolutions, a progressive increase in the engagement of the fear-learning–related brain state over time during post-conditioning rest.

If fear learning induces a meaningful reorganization of large-scale brain-state dynamics, this reorganization would be expected to continue evolving during the post-learning resting period rather than emerge as an instantaneous shift. If so, a temporally-graded shift in these states would be expected. To examine how fear-learning–induced brain dynamics evolve over time following conditioning, we quantified the engagement of the fear-learning–related brain state during the post-conditioning resting period. Specifically, post-conditioning resting-state fMRI time series were segmented into sliding temporal windows of varying lengths. Within each window, we computed the occurrence rate (OCR) of the fear-learning–related brain state, enabling assessment of how state engagement changes as a function of time across multiple temporal resolutions. Across all window lengths, the OCR of the fear-learning–related brain state exhibited a consistent increase over time following fear acquisition (Fig. 3). Early windows showed relatively low engagement of this state, whereas progressively later windows were characterized by higher OCR, indicating a gradual accumulation of fear-learning–related state engagement over the course of the spontaneous resting period. Mean dwell time (DT) showed a similar increasing trend (Suppl. Fig. 5). This post-learning temporal dynamic supports the idea that this gradual shift might represent the initial phase of memory consolidation associated with threat learning.

### Fear-learning–induced brain reorganization predicts extinction memory

**Fig. 4:**
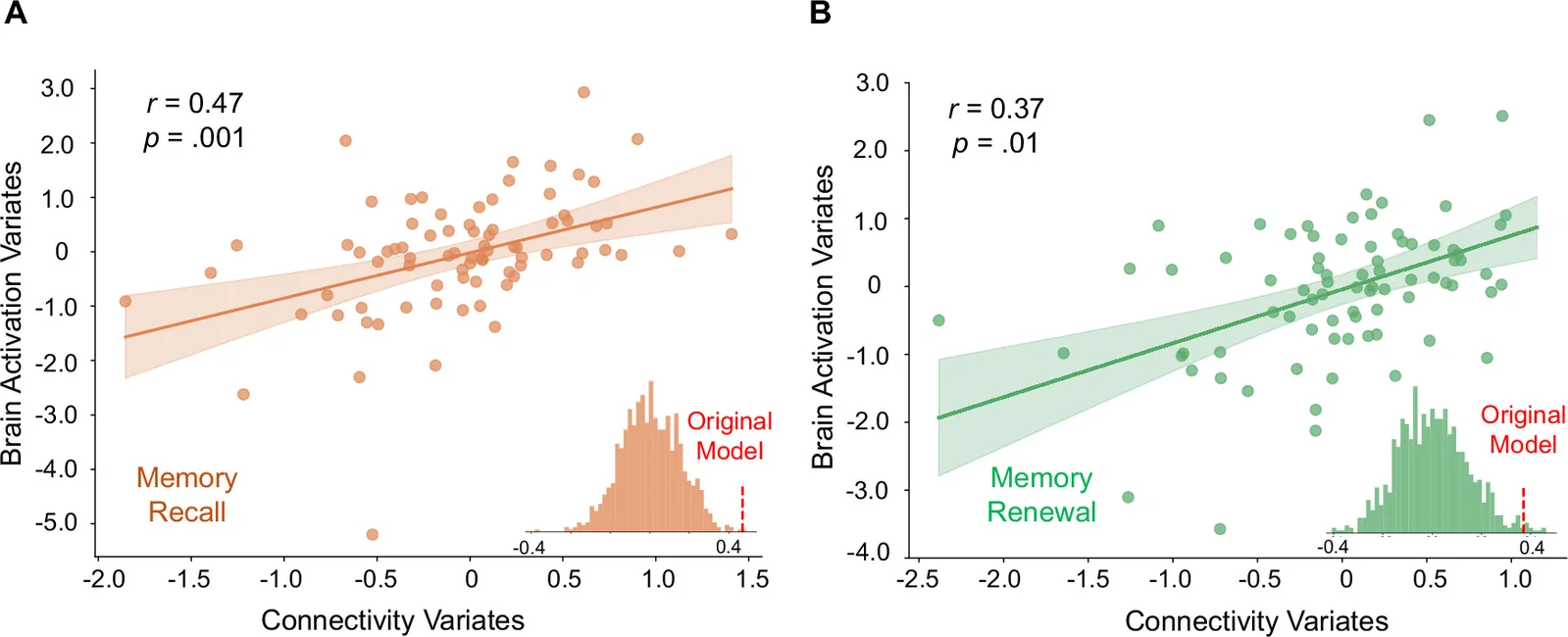
Fear-learning–induced brain reorganization predicts memory recall. (**A**) and renewal (**B**) brain activation. Connectivity variates were defined by the difference matrix of functional connectivity from post-conditioning minus pre-conditioning brain state 1 for each participant. Brain activation variates were activation value for each participant in recall/renewal phase. Statistical analyses were performed using data from 80 independent healthy participants (*n* = 80). Each data point represents one independent participant. Solid lines indicate least-squares linear fits, and shaded bands represent the 95% confidence intervals for the fitted mean responses. Prediction performance was evaluated using Pearson correlation between observed and predicted values. Statistical significance was assessed using two-sided permutation tests (1000 permutations). Insets show the null distributions generated by permutation testing; the red dashed line indicates the correlation coefficient obtained from the original (non-permuted) model. Correlation coefficients (*r*) and exact *P*-values are shown.

To determine whether fear-learning–induced brain reorganization predicts subsequent extinction-memory expression, we applied regularized canonical correlation analysis (rCCA) to relate conditioning-induced functional connectivity reorganization within the identified fear-learning state (predictor variables) to multivariate patterns of brain activation during extinction-memory recall and renewal (Fig. 4). Fear-learning–related connectivity changes (Suppl. Fig. 6) achieved significant out-of-sample predictive relation with the extinction memory-expression variate for both recall and renewal conditions (Suppl. Fig. 7), with *r*(78) = 0.47, permutation tests *p* = 0.001 (memory recall) and *r*(78) = 0.37, permutation tests *p* = 0.01 (memory renewal). Notably, this predictive relationship was specific to the TMS modulation condition. When the same predictive framework was applied to the natural extinction (CS+NoTMS), no significant association was observed for either recall (*r*(78) = −0.13, *p* > .05) or renewal (*r*(78) = 0.03, *p* > .05). Direct comparison of predictive performance between conditions further showed significantly greater prediction performance under TMS-modulated than natural extinction for both recall (Δr = 0.60, permutation *p* = 0.002) and renewal (Δr = 0.34, permutation *p* = 0.04). These findings demonstrate that conditioning-induced functional connectivity reorganization exhibited significantly stronger brain–brain predictive relationships under the TMS-modulated extinction condition than under natural extinction.

**Fig. 5:**
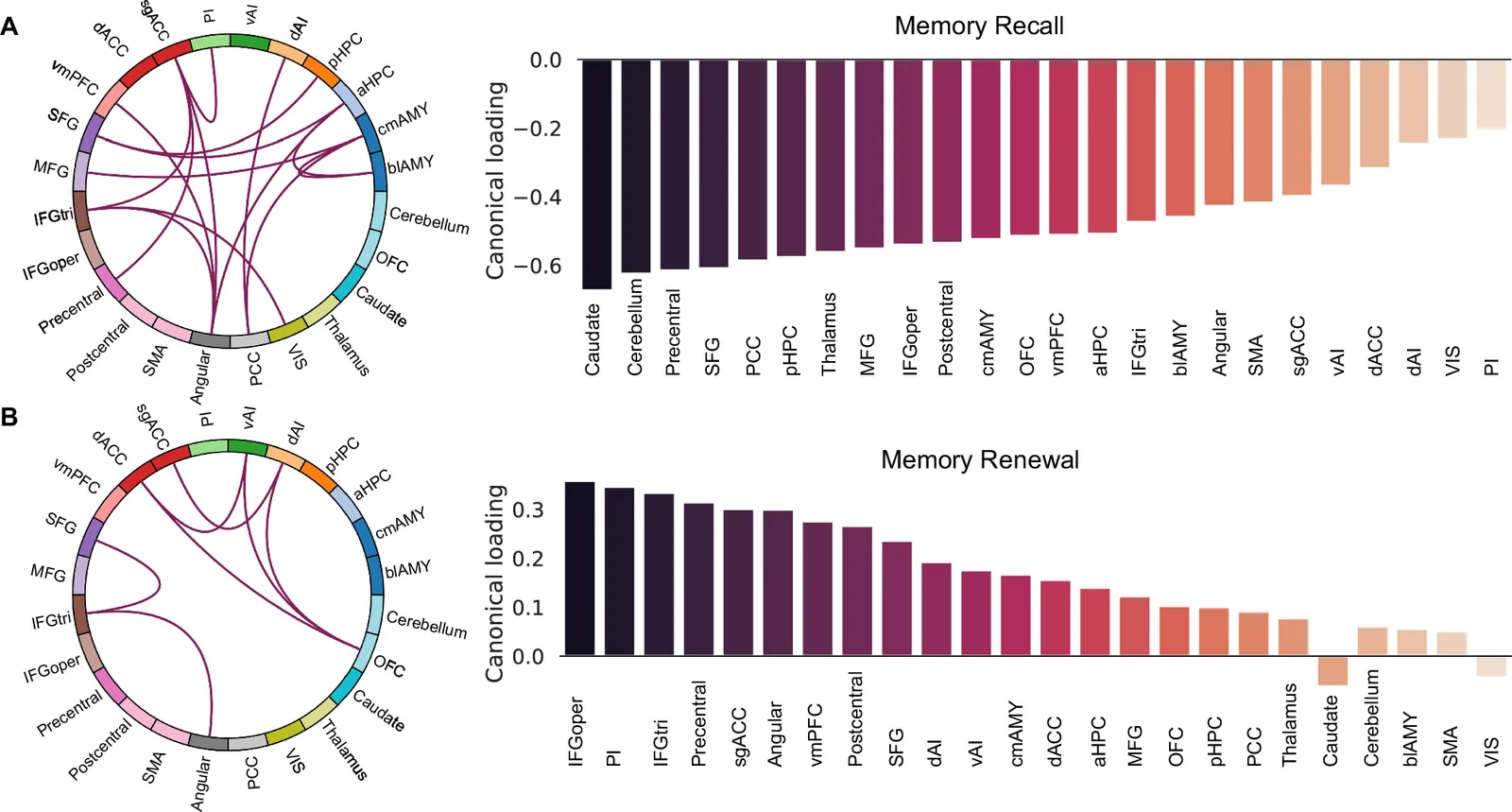
Canonical loading profiles reveal neural signatures linking fear-learning–induced connectivity reorganization to memory recall and renewal. (**A**) Recall and (**B**) renewal brain activation. Canonical loading analysis was performed to characterize the relative contributions of individual features to the canonical variates on both sides of the model.

To further characterize the neural signatures underlying these predictive relationships, we examined canonical loadings from the rCCA models to identify features contributing most strongly to each prediction (Fig. 5). For the recall condition, connectivity reorganization involved widespread contributions across the extended threat-circuit network. Canonical loading analysis showed that a large proportion of the 24 nodes contributed to the brain-organization variate. On the outcome side, the strongest contributors to the memory-expression variate were localized primarily to the caudate, cerebellum, precentral gyrus, superior frontal gyrus (SFG), and posterior cingulate cortex (PCC). In this model, greater conditioning-induced brain reorganization was associated with reduced activation in these regions, as reflected by negative canonical loadings. In contrast, prediction of renewal-phase activation was driven by a more selective set of learning-related connectivity changes, primarily involving orbitofrontal cortex (OFC), dorsal anterior insula (dAI), ventral anterior insula (vAI), and dorsal anterior cingulate cortex (dACC). On the outcome side, the strongest contributors included IFG opercularis, posterior insula (PI), IFG triangularis, precentral gyrus, and subgenual ACC (sgACC). In this case, greater conditioning-induced brain reorganization was associated with increased activation in these regions, as indicated by positive canonical loadings.

To evaluate the robustness of these neural signatures, we performed a non-parametric bootstrap stability analysis (1000 iterations) for both connectivity-side and activation-side canonical loading profiles. Across both recall and renewal models, the dominant connectivity features and regional activation patterns identified in the primary rCCA analyses were consistently reproduced across bootstrap resamples. Additionally, the highest-contributing functional connections and brain regions exhibited stable loading estimates and preserved their relative importance rankings across iterations (Further details are provided in Supplementary Fig. 8).

### Behavioral results

**Fig. 6:**
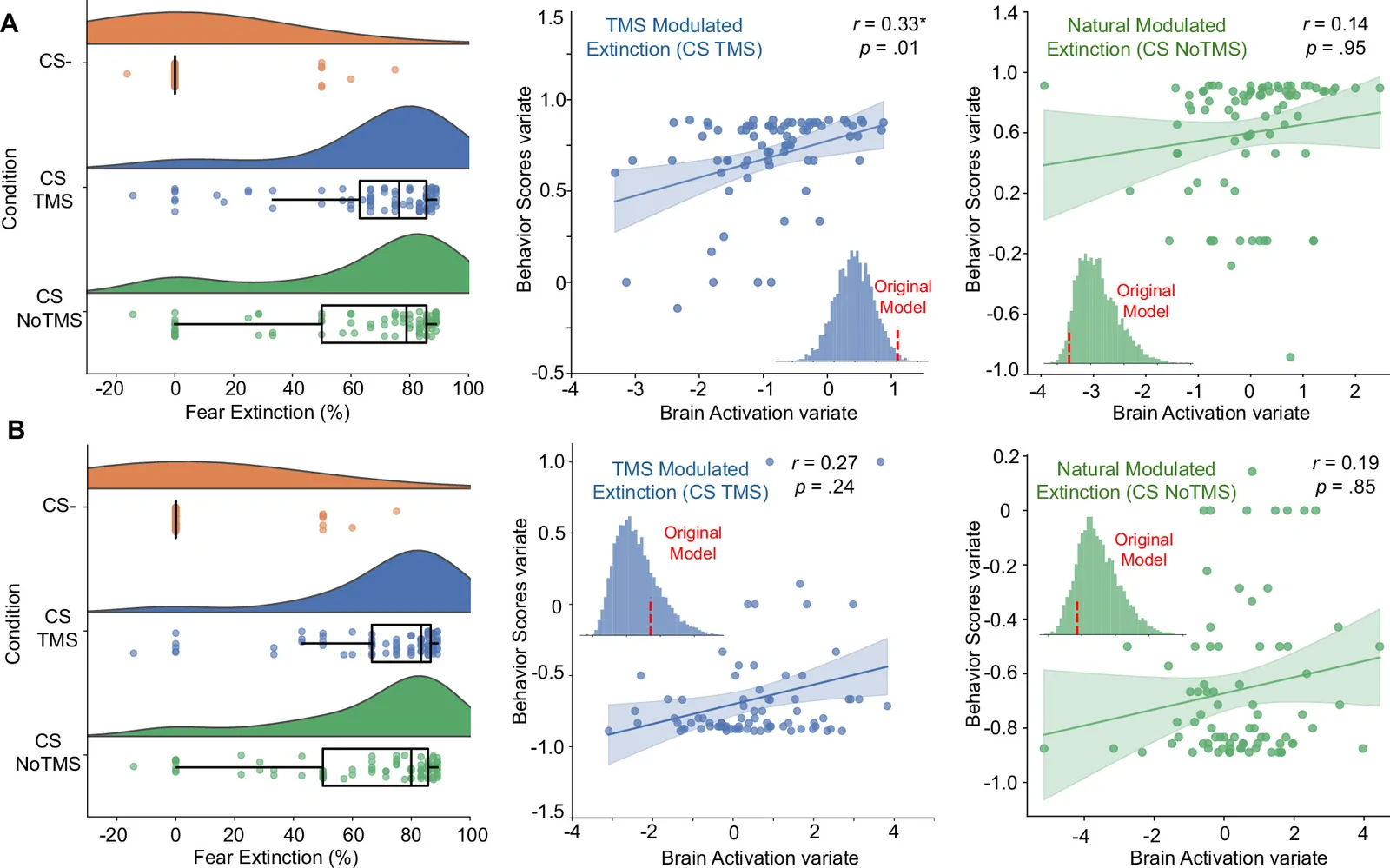
Behavioral expression of extinction memory and its association with neural measures. **A** Association between brain-derived neural variates and subjective fear ratings during TMS-modulated and natural extinction memory recall. **B** Association between brain-derived neural variates and subjective fear ratings during TMS-modulated and natural extinction memory renewal. Statistical analyses were performed using data from 80 independent healthy participants (*n* = 80). Each data point represents one independent participant; no technical replicates were included. Pearson correlation coefficients were used to assess brain–behavior associations. Boxes indicate the interquartile range (Q1–Q3), the centre line indicates the median, and whiskers extend to 1.5 × IQR. Insets show the null distributions generated by permutation testing, and the red dashed lines indicate the correlation coefficients obtained from the original (non-permuted) models. Brain–behavior associations were assessed using Pearson correlation. Statistical significance was determined using two-sided permutation tests (1000 permutations).

Behavioral indices of fear reduction were quantified using subjective fear ratings obtained during conditioning and memory expression phase (Fig. 6A, B). For both recall and renewal, CS+ showed a clear reduction of fear level after extinction learning. This reduction was observed in both the TMS and NoTMS conditions, whereas fear ratings to the safety cue (CS − ) remained stable and close to baseline across all phases. Although the magnitude of fear reduction did not differ significantly between the TMS and NoTMS conditions at the group level, the TMS condition exhibited a numerically greater degree of fear attenuation in both memory phases. This pattern suggests that TMS may enhance the expression of extinction memory, even in the absence of robust group-level behavioral differences. Consistent with the subjective fear ratings, psychophysiological analyses of skin conductance responses (SCR) confirmed successful fear acquisition during conditioning and progressive attenuation of conditioned responses during memory recall, supporting the effectiveness of the conditioning and extinction procedures (Suppl. Fig. 1).

To further examine the relationship between neural dynamics and behavioral expression, we conducted multivariate correlation analyses linking individual differences in behavior to brain-derived measures. As shown in Fig. 6A, B, TMS modulated extinction recall memory (*r*(78) = 0.33, *p* = .01) was significantly associated with subjective fear ratings. In contrast, no significant correlation was observed between natural extinction (CS+NoTMS) memory and behavioral scores (recall: *r*(78) = 0.14, *p* = .95; renewal: *r*(78) = 0.19, *p* = .85). Although the renewal analysis did not reach statistical significance following permutation testing, the effect size remained larger in the TMS condition than in the natural extinction condition. These findings suggest that significant brain–behavior associations were observed only for TMS-modulated extinction recall memory, while the overall pattern remained consistent with stronger brain–behavior coupling under TMS-modulated compared with natural extinction learning.

## Discussion

In the present study, we addressed two fundamental questions concerning the neural dynamics of human fear learning and its translational relevance. Specifically, we first demonstrated that fear learning selectively reshapes the temporal organization of spontaneous brain states, characterized by increased engagement and altered transition dynamics of a fear-related brain state within a threat-circuit. Second, we show that connectivity reorganization induced by fear learning was predictively associated with subsequent memory recall and renewal following extinction under TMS-neuromodulation. We establish a dynamic, explainable neural signature of fear learning that not only characterizes how the brain adapts to threat but also serves as a candidate biomarker for extinction memory expression.

Contemporary models of psychopathology increasingly emphasize that maladaptive learning processes in anxiety- and trauma-related disorders arise not solely from abnormalities within static and isolated brain regions, but from dysregulated dynamics across large-scale functional networks^1,39,40^. From a systems-level perspective, pathological fear may reflect impaired temporal coordination and flexibility among distributed circuits supporting threat detection, salience processing, memory consolidation, and regulatory control^41–46^. Previous studies have established that fear learning induces persistent reorganization of resting-state functional connectivity, with such changes being associated with subsequent fear expression and extinction outcomes. Extending this literature, our state-based framework characterizes how fear learning reshapes the temporal architecture of spontaneous brain activity through changes in the engagement, persistence, and switching of recurring large-scale brain states. Within this framework, the Hidden Markov Model offers a critical advantage by jointly modeling both spatial topologies and temporal transition probabilities at single-TR resolution^47,48^. Unlike conventional sliding-window approaches that rely on arbitrary temporal smoothing, or frame-wise clustering methods that discard temporal information, this generative probabilistic framework enables characterization of rapid, non-linear transitions among recurring brain states following fear learning. Leveraging this approach, we demonstrated that fear learning selectively amplified a distributed threat-related brain state shortly after learning. This state was characterized by coordinated engagement of medial temporal, insular, cingulate, and sensorimotor regions, consistent with an integrated network supporting threat encoding, interoceptive salience, and defensive action preparation. Notably, the strongest contributors included the anterior hippocampus, posterior insula, posterior hippocampus, precentral gyrus, and basolateral amygdala. The hippocampal and amygdala components are central to associative threat learning and contextual memory encoding, the posterior insula supports interoceptive and salience processing of aversive stimuli, and the precentral cortex likely reflects engagement of threat-related sensorimotor circuitry associated with motor preparation, action readiness, defensive preparation, and threat anticipation during fear learning. Critically, this fear-learning–related brain state did not merely emerge transiently after conditioning but exhibited a progressive increase in engagement throughout the post-conditioning resting period. That suggests that spontaneous brain activity continues to reorganize after conditioning, consistent with an ongoing systems-level consolidation process rather than a static imprint of learning. This observation naturally raises an important unresolved question: how long these state-level dynamics persist following fear learning. Prior work has suggested that fear-memory consolidation can continue for several hours following acquisition^24,49,50^, with most studies focusing primarily on the duration of post-learning neural processes. Although the present study cannot directly address this question because only a single post-conditioning resting-state fMRI run was acquired, the CAP framework provides a natural extension of these traditional approaches. By explicitly modeling the temporal evolution of recurring brain states, CAP has the potential to characterize not only how long consolidation-related dynamics persist, but also how rapidly they emerge and how stable their engagement becomes over time. Addressing these questions will require repeated resting-state fMRI acquisitions spanning the consolidation period, allowing future longitudinal studies to characterize the full trajectory of conditioning-related brain-state dynamics.

A central goal of contemporary neuroscience is to link large-scale brain organization to individual variability in treatment response, neuromodulation effects, and cognitive change^51–55^. Cross-validated predictive modeling has become a powerful framework for capturing the coupling between brain connectivity and functional outcomes at the level of individual subjects^56–58^. Building on our characterization of fear-learning–induced brain dynamics, we further demonstrate that connectivity reorganization within the fear-learning–related brain state serves as a candidate neural biomarker of subsequent extinction memory retrieval and expression. Specifically, functional connectivity changes from pre- to post-conditioning within this dynamic state significantly predicted individual patterns of brain activation during both memory recall and renewal. Crucially, the predicted activation patterns can be interpreted within a representational framework of threat versus safety processing. For example, by quantifying the relative activation across the 24-node threat-circuit regions, the resulting activation profile can reflect whether the current brain state is dominated by threat-related or safety-related circuitry. Notably, although both CS + TMS and CS+NoTMS conditions exhibited fear extinction, only following TMS-paired extinction did fear-learning–induced brain reorganization predict later recall and renewal activations. This dissociation may reflect an important property of neuromodulation. A growing body of literature suggests that focal brain stimulation does not remain restricted to the stimulated cortical site, but propagates through distributed large-scale functional networks. Such perturbations have been shown to vary substantially across individuals, contributing to considerable heterogeneity in both neural and behavioral responses to TMS^59–61^. Within this framework, TMS may alter the neural implementation of extinction learning relative to natural extinction. Rather than affecting all participants uniformly, stimulation may allow pre-existing individual differences in fear-learning–related brain organization to exert a stronger influence on subsequent memory-expression processes, thereby increasing the detectability of brain–brain predictive relationships. Meanwhile, the absence of significant prediction in the no-TMS condition does not indicate that Day 1 brain organization is unrelated to extinction without stimulation. It only indicates that the specific Day 1 neural signature examined here did not reliably predict the corresponding Day 3 activation pattern.

Although both recall and renewal index expressions of extinction memory, they reflect distinct cognitive and contextual processes. Canonical loading analyses revealed dissociable circuit-level signatures for recall and renewal. During memory recall, fear-learning–induced reorganization was distributed across a broad set of connections spanning subcortical, sensorimotor, and medial cortical regions. On the brain-organization variate, prominent contributions arose from connectivity changes involving hippocampal, striatal, cerebellar, and motor-related regions, suggesting that learning-induced reconfiguration within this fear-related state engages distributed systems supporting memory retrieval, action preparation, and contextual integration. Correspondingly, on the memory-expression variate, the most strongly weighted regions included the caudate, cerebellum, precentral gyrus, superior frontal gyrus, and posterior cingulate cortex, indicating that recall-related activation patterns reflect coordinated engagement of motor, cognitive control, and default-mode components. In contrast, renewal exhibited a more selective and regulatory-oriented signature. Fear-learning–related reorganization contributing to renewal prediction was dominated by connectivity changes among orbitofrontal, insular, and cingulate regions, including OFC, dorsal and ventral anterior insula, and dorsal ACC—regions centrally implicated in salience evaluation, interoceptive processing, and regulatory control. On the outcome side, renewal-related activation was most strongly weighted in inferior frontal operculum, posterior insula, inferior frontal gyrus, precentral cortex, and subgenual ACC, highlighting a network configuration consistent with conflict monitoring, affective regulation, motor readiness and defensive preparation during context-driven threat re-expression. The divergence of these neural signatures underscores that extinction memory expression is not monolithic but is instantiated through task- and context-dependent configurations of distributed brain systems shaped by prior fear learning.

Subjective fear ratings indicated that extinction was evident in both stimulation conditions: fear responses decreased from conditioning to the post-extinction memory phase for both CS + TMS and CS+NoTMS, whereas CS− showed minimal change relative to baseline. Although the magnitude of behavioral extinction did not significantly differ between CS + TMS and CS+NoTMS, extinction tended to be stronger in the TMS condition. To further examine whether the observed TMS-related effects could be attributed to pre-existing differences between the two conditioned cues, we explored the relationship between fear-learning–induced brain reorganization and subsequent behavioral change separately for the CS + TMS and CS+NoTMS conditions (i.e., two conditioned threat cues CSp1 and CSp2 established during Day 1). Following permutation-based inference, none of these brain–behavior associations reached statistical significance (Supplementary Fig. 9). Importantly, the overall effect-size patterns were comparable across the two CS+ conditions, providing no evidence that one cue exhibited systematically stronger coupling between fear-learning–induced reorganization and behavioral outcome prior to extinction learning. These findings suggest that the stronger brain–brain predictive relationships observed under TMS-modulated extinction are unlikely to be explained by intrinsic differences between the conditioned cues established during fear acquisition.

Several limitations should be acknowledged. One limitation is that the number of connectivity features exceeded the sample size, resulting in a high-dimensional prediction setting^62^. Although regularization, nested cross-validation, permutation testing, and bootstrap stability analyses were implemented to mitigate overfitting and assess model robustness, replication in larger independent cohorts will be important for establishing the generalizability of the identified neural signatures. Second, the present study focused exclusively on healthy participants. While this approach enabled a controlled characterization of fear-learning–related brain dynamics, it precludes direct inference about clinical populations in whom maladaptive fear learning is most relevant. In particular, it remains unknown whether the fear-learning–induced brain state identified here differs in prevalence, dynamics, or predictive utility between healthy individuals and patients with anxiety- or trauma-related disorders. Future work incorporating patient cohorts will be essential to determine whether alterations in this dynamic brain state represent a mechanistic marker of pathological fear learning and to refine dynamic neural phenotypes associated with impaired adaptive learning. Third, although our stimulation protocol was designed as a facilitatory neuromodulation intervention targeting the DLPFC-vmPFC regulatory circuit, our experimental protocol did not include sham stimulation or active stimulation over a control region. Therefore, although the observed predictive relationship was significantly stronger under the TMS-modulated condition within the present design, we cannot determine whether this finding reflects modulation of the targeted DLPFC-vmPFC circuit or nonspecific effects of active stimulation. A more mechanistic understanding of this effect will require future studies incorporating sham stimulation, active control stimulation sites, individualized electric-field modeling, on-target versus off-target stimulation comparisons, and multimodal neuroimaging approaches capable of directly characterizing stimulation-induced changes in large-scale brain networks.

In summary, this study provides a dynamic perspective of how human fear learning reshapes large-scale brain organization and how these changes carry predictive value for later memory expression under neuromodulation. By integrating coactivation pattern analysis with multivariate predictive modeling, we identified a fear-learning induced brain state whose engagement and dynamical flexibility increased following conditioning and continued to strengthen over time during post-conditioning. Critically, the magnitude of this learning-induced reorganization predicted individual differences in neural responses during subsequent memory recall and renewal, selectively under TMS-modulated extinction. These findings demonstrate that transient brain dynamics during fear learning are not merely epiphenomenal, but constitute a stable neural biomarker or signature linking learning history to future regulatory outcomes. The present study advances a dynamic neurobiological framework of fear learning and provides a translational bridge from mechanistic brain-state dynamics to individualized prediction of extinction memory expression.

## Methods

### Participants

Eighty-seven healthy adults (43 males and 44 females) with an age range of 18–60 years (mean ± SD = 28.79 ± 9.20 years) were recruited at The University of Texas Health Science Center at Houston (UTHouston). Sex was determined based on participant self-report and was not a prespecified factor in the study design. Sex-disaggregated inferential analyses were not performed because the study was not designed or powered to examine sex-specific effects. Exclusion criteria included any neurological, psychiatric, or MRI contraindications. All participants provided written informed consent in accordance with the Declaration of Helsinki and protocols approved by the Institutional Review Board of UTHouston. Participants received financial compensation for their participation. Seven participants were excluded from subsequent analyses because of incomplete imaging data (missing post-conditioning resting-state scans, *n* = 4; missing Day 3 memory-expression imaging data, *n* = 3), resulting in a final sample of 80 participants (41 males, 39 females; mean age = 28.36 ± 8.33 years, range = 18–60 years).

### Experimental design

Participants completed a three-day fear-learning and extinction paradigm^14^ (Fig. 1). Conditioned threat learning was induced using visual cues paired with aversive electric foot shocks (BIOPAC 160 Systems, Goleta, CA).

On Day 1 (fear conditioning), participants were presented with visual stimuli of three different colors. Two colors served as conditioned threat cues (CS + ), each presented in a separate block, whereas a third color served as a safety cue (CS − ) and was presented across both blocks. Each trial began with presentation of a contextual image (office) for 3 s, followed by presentation of the CS for 6 s. On reinforced CS+ trials, a 500-ms electric shock was delivered at CS offset, whereas CS− trials were never reinforced. The mean inter-trial interval was 15 s. Shock intensity was individually calibrated to be highly aversive but not painful. Resting-state fMRI scans were acquired immediately before and after conditioning to characterize fear-learning–related changes in spontaneous brain dynamics.

On Day 2 (extinction learning), participants underwent extinction training in the absence of shock. The same three conditioned cues were presented across two blocks, with four trials for each CS+ and four trials for the CS − . One CS+ was paired with brief trains of repetitive transcranial magnetic stimulation (rTMS; CS + TMS), whereas the other CS+ and the CS− were presented without stimulation (CS+NoTMS and CS − , respectively). rTMS was delivered to an individually defined target in the left dorsolateral prefrontal cortex (DLPFC), as detailed below. This within-subject design enabled comparison of extinction-memory expression following TMS-modulated versus non-TMS extinction.

On Day 3, participants completed extinction-memory recall and renewal testing during fMRI. Conditioned cues were presented without shock. Recall was assessed in the same contextual environment used during extinction learning on Day 2, whereas renewal was assessed in the original conditioning context from Day 1, allowing extinction-memory retrieval to be distinguished from context-dependent return of conditioned threat. The order of CS + TMS and CS+NoTMS blocks, as well as cue–color assignments, was randomized across participants. Subjective fear ratings and skin conductance responses were collected to characterize conditioned fear expression.

### TMS Protocol and Target Selection

Repetitive transcranial magnetic stimulation (rTMS) was administered during extinction learning on Day 2 using a MagPro X100 stimulator equipped with an MC-B65 figure-of-eight coil (MagVenture, Farum, Denmark). Neuronavigation was performed using Brainsight 2.4 (Rogue Research Inc.) based on each participant’s high-resolution T1-weighted anatomical image.

Because the vmPFC is not directly accessible to conventional TMS, an individualized connectivity-informed targeting approach was used to identify a surface-accessible cortical site functionally coupled with the vmPFC. For each participant, seed-based functional connectivity maps derived from fMRI acquired during fear conditioning were used to identify a target within the left posterior DLPFC showing strong functional connectivity with an a priori defined vmPFC region of interest. The individualized DLPFC target was registered to the participant’s anatomical reconstruction and maintained throughout stimulation using real-time neuronavigation.

Resting motor threshold (rMT) was determined over the hand area of the primary motor cortex as the minimum stimulation intensity eliciting visible hand muscle twitches in at least 3 of 6 trials. During extinction learning, rTMS was delivered at 20 Hz for 300 ms (6 pulses per train) at 100% of the individual rMT. Four trains were administered, yielding a total of 24 pulses. The brief train duration was selected based on stimulation protocols used in rodent studies and in our prior preliminary study^1,33^. rTMS was paired only with CS + TMS trials, whereas CS+NoTMS and CS− trials were presented without stimulation. Coil positioning was continuously monitored using Brainsight, and stimulation was administered only when spatial error was <1 mm. Additional details regarding target localization, neuronavigation and rTMS implementation are provided in the Supplementary Information.

### Neuroimaging Acquisition and Preprocessing

Neuroimaging data were acquired on a 3 T Siemens Prisma using a 64-channel head coil. High-resolution T1-weighted anatomical images were acquired for spatial normalization. Functional images were collected with a gradient-echo echo-planar imaging (EPI) sequence (TR = 1500 ms; TE = 35 ms; flip angle=62°; voxel size=2×2×2 mm; FOV = 208 mm; 72 slices, interleaved, multi-band acceleration factor = 4).

Data preprocessing was performed using *fMRIPrep* version 20.0.6^63^. T1-weighted images underwent intensity nonuniformity correction (N4BiasFieldCorrection, ANTs 2.3.3), skull stripping, tissue segmentation (gray matter, white matter, CSF), and nonlinear normalization to MNI152NLin2009cAsym space (ANTs’ antsRegistration). Functional images were corrected for head motion with mcflirt (FSL), adjusted for slice-timing using 3dTshift (AFNI), and co-registered to the corresponding T1-weighted image with boundary-based registration using flirt (FSL). The resulting functional data were normalized to standard MNI space and resampled to 2 mm isotropic resolution using Lanczos interpolation.

### Dynamic Coactivation Pattern Analysis

We implemented a data-driven coactivation pattern (CAP) analysis that captures momentary configurations of large-scale neural activity^26,64^. In this framework, individual BOLD volumes are treated as instantaneous snapshots of whole-brain activation and grouped into a finite set of recurring coactivation states, enabling the identification of transient brain states that emerge during spontaneous activity independent of task timing or experimental condition.

Pre- and post-conditioning resting-state fMRI time series were concatenated across participants, yielding a matrix of size T × R, where T denotes the total number of time points and R the number of brain regions. This concatenated dataset served as the input for CAP modeling, allowing state estimation to be driven by shared spatiotemporal structure across individuals and learning phases.

To determine the appropriate number of coactivation states, clustering solutions ranging from 2 to 14 states were evaluated using complementary model-selection criteria, including the Davies–Bouldin index and the Ray–Turi index, which jointly assess within-state compactness and between-state separability^65^. Both metrics converged on an optimal solution comprising five distinct CAP states (Supplementary Fig. 2).

Temporal organization of CAP expression was modeled using a Gaussian hidden Markov model (HMM) with diagonal covariance, trained on the full dataset encompassing both pre- and post-conditioning scans. This probabilistic framework enabled estimation of state-specific temporal trajectories and transitions, thereby capturing learning-related reorganization in dynamic brain-state engagement.

Decoded state sequences were subsequently organized at the subject and phase level and used to derive multiple dynamic indices, including state occurrence rate (OCR), mean dwell time (DT), state transition probabilities (TP), and trajectory entropy (EMT). All these metrics provided a multidimensional characterization of how fear learning reshapes the temporal structure and stability of large-scale brain states^66–68^. Detailed mathematical formulations of the HMM, state decoding procedures, and computation of dynamic indices are provided in the Supplementary Information.

### Estimation of functional connectivity matrix and brain activation

Functional connectivity (FC) was estimated using an extended threat-circuit atlas comprising 24 regions of interest^18^. For each ROI, regional time series were extracted by averaging the BOLD signal across all voxels within the region. To minimize the impact of head motion, time points with framewise displacement (FD) exceeding 0.5 mm were censored prior to connectivity estimation. For each participant, FC matrices (24 × 24) were computed using pairwise Pearson correlations between ROI time series. For the state-specific analysis, BOLD time points assigned to State 1 were extracted separately from the pre- and post-conditioning resting-state scans, and FC matrices were computed using only these state-specific samples. Fear-learning-induced connectivity reorganization was quantified as the difference between post- and pre-conditioning State 1 FC. The upper triangular elements of each matrix (excluding the diagonal), corresponding to 276 unique functional connections [24 × (24 − 1)/2], were vectorized and used as input features for predictive modeling.

We used a univariate general linear model (GLM) implemented in the Nistats 0.0.1rc toolbox to estimate activation maps of different stimuli. Given evidence from both human and animal studies showing distinct neural activations across trials even within an experimental phase, we divided trials of each CS type into trial-blocks (4 trials per trial-block) and separately modeled the brain activity to each trial block. During the conditioning and extinction phases, four trial blocks were modeled for each CS. During the recall and renewal phases, two trial blocks (Early, Late) were modeled for CS + TMS, CS+NoTMS, and their corresponding CS− conditions. For each trial-block, the regressor were modeled as boxcar functions time-locked to the CS presentations and convolved with the canonical hemodynamic response function (HRF). Activation estimates from the first trial block (Early) of each condition were used for subsequent predictive analyses.

### Multivariate prediction of memory expression from fear-learning–induced brain reorganization

We implemented a multivariate framework based on regularized canonical correlation analysis to test whether fear learning–induced brain reorganization predicts later memory expression. rCCA is a multivariate statistical method^69^ that identifies pairs of latent components (canonical variates) that maximize the correlation between two high-dimensional variable sets—in this case, state-specific functional connectivity reorganization following fear learning and brain activation patterns during subsequent memory recall or renewal. Compared with classical CCA, rCCA incorporates an L2 regularization term that stabilizes covariance estimation and reduces overfitting, making it well suited for high-dimensional neuroimaging data with limited sample sizes. Model regularization parameters were optimized via a grid-search procedure spanning values from 0 to 1 in increments of 0.05. To prevent information leakage during rCCA model fitting, hyperparameter selection was performed exclusively within the training data using an inner 10-fold cross-validation procedure within a nested cross-validation framework. The optimal regularization parameters identified in the inner loop were then fixed and applied to held-out test data to evaluate out-of-sample predictive performance. Although rCCA yields multiple pairs of canonical variates, primary analyses focused on the first canonical mode, which captures the maximal shared variance between fear-learning–related brain reorganization and later memory-related brain activation. Statistical significance of this canonical association was assessed using a permutation testing procedure, confirming that the observed brain–brain correspondence exceeded chance-level expectations. To characterize neural signatures contributing to the predictive models, canonical loadings were computed as the Pearson correlation between each original brain feature (X-side: functional connectivity; Y-side: brain activation) and the corresponding canonical variate derived from the model. For visualization of connectivity-side neural signatures, significant functional connections were identified using false discovery rate (FDR) correction (*p* < .05) across all connectivity features. To evaluate the stability of the identified neural signatures, we additionally performed a non-parametric bootstrap procedure (1000 iterations). In each iteration, participants were resampled with replacement and canonical loadings were recomputed. Bootstrap distributions were then used to estimate confidence intervals and assess the stability of both connectivity-based and activation-based loading profiles across resamples.

### Behavioral and psychophysiological assessment of fear learning and extinction

Behavioral indices of fear learning and extinction were assessed using computerized subjective fear ratings collected inside the MRI scanner. After completion of the conditioning phase, participants completed a questionnaire in which they rated how afraid they were of each stimulus color at both the beginning and the end of the conditioning session. Ratings were provided using a 9-point numerical rating scale (1 = least afraid, 9 = most afraid) by selecting the corresponding response with a computer mouse. The same procedure was repeated following the recall and renewal sessions to assess subjective fear memory expression.

Skin conductance responses (SCRs) were also continuously acquired during the conditioning, memory recall, and renewal phases to provide an implicit psychophysiological measure of conditioned fear expression. Detailed SCR acquisition, preprocessing, and statistical analyses are described in the Supplementary Information.

To quantify individual differences in extinction-related fear reduction, we computed an extinction degree (ED) defined as the normalized change in fear ratings from conditioning to memory expression. Specifically, ED was calculated as the difference between fear ratings obtained after extinction recall phase (i.e., after memory consolidation) and fear ratings obtained immediately after conditioning, divided by the post-conditioning fear level:

$${ED}=\frac{{{Fear}\,{Rate}}_{{Conditioning}}-{{Fear}\,{Rate}}_{{Extinction}}}{{{Fear}\,{rate}}_{{Conditioning}}}\times 100\%$$

To examine the behavioral relevance of fear-learning–induced brain reorganization, we tested the relationship between ED and the multivariate brain variates derived from the predictive modeling analyses. Specifically, correlations were computed between individual ED scores and the canonical variates capturing fear-learning–related brain reorganization and subsequent memory-related brain activation, allowing assessment of whether neural reorganization during fear learning and its predictive mapping to memory expression covaried with subjective extinction outcomes.

### Statistical analysis

All dynamic metrics were compared pre versus post conditioning using two-sided paired-sample t-tests with FDR correction (*p* < 0.05). Permutation testing (1000 permutations) was used to assess the statistical significance of predictive associations between fear learning-induced brain reorganization and brain activation of extinction memory. Age and sex were additionally evaluated as covariates in sensitivity analyses of the CAP and rCCA models to assess the robustness of the primary findings. A non-parametric bootstrap procedure was performed to evaluate the stability of the identified neural signatures. Effect sizes are reported as Pearson’s correlation coefficients (*r*) or Cohen’s *d*z for paired comparisons, as appropriate. Unless otherwise specified, all behavioral, psychophysiological, and neuroimaging measurements were acquired repeatedly from the same participants across the three-day experimental protocol.

### Reporting summary

Further information on research design is available in the Nature Portfolio Reporting Summary linked to this article.

## Supplementary information

Supplementary Information

Reporting Summary

Transparent Peer Review file

## Source data

Source Data

## Data Availability

Source data are provided with this paper. The participant-level neuroimaging and behavioral data of this study are not publicly available because public deposition is restricted by the conditions of the institutional ethical approval and data-sharing requirements for the protection of participant privacy. Access to de-identified data may be requested from the corresponding author, Mohammed R. Milad (Mohammed.R.Milad@uth.tmc.edu). Requests should include a description of the proposed research and will be reviewed in accordance with applicable institutional and NIH data-sharing requirements. Data will be made available to qualified researchers for research purposes following approval of the request and completion of any required institutional data-use agreement. Source data are provided with this paper.

## Code availability

The code used for data processing and statistical analyses is publicly available through the GitHub repository and has been permanently archived in Zenodo^70^ (https://doi.org/10.5281/zenodo.21829352). The repository includes scripts for time-series extraction, functional connectivity computation, co-activation pattern (CAP) analysis (including the calculation of four CAP-related metrics), and rCCA-based prediction analyses. The implementation builds upon publicly available Python libraries including CCA-Zoo https://cca-zoo.readthedocs.io/en/latest/modules/classes.html, Nilearn https://nilearn.github.io/stable/index.html for fMRI data processing, and Seaborn https://seaborn.pydata.org/ for data visualization.
