# Supplementary material for: Fear learning–induced brain dynamics predict individual extinction memory expression following transcranial magnetic stimulation
Source: Nat Commun. 2026 Oct 5;17:11357. doi: 10.1038/s41467-026-78345-8 (PMC13728185; doi:10.1038/s41467-026-78345-8)
Supplement: Supplementary file 1 [file 41467_2026_78345_MOESM1_ESM.pdf]

Supplementary Information

Fear Learning–Induced Brain Dynamics Predict Individual Extinction Memory Expression Following Transcranial Magnetic Stimulation

Kai Zhang, Lihan Cui, B Isabel Moallem, Hani Meelad, Zakiya Atiyah, Muhammad Badarnee, Isabella Mason, Zhenfu Wen, Mark S. George, Mohammed R. Milad

Methods

TMS Protocol

1.TMS Administration and Neuronavigation

Transcranial magnetic stimulation (TMS) was administered during extinction learning on Day 2 using a MagPro X100 stimulator equipped with an MC-B65 figure-of-eight coil (MagVenture, Farum, Denmark). Neuronavigation was performed with Brainsight 2.4 (Rogue Research Inc.) to ensure accurate and reproducible coil positioning relative to each participant's individual brain anatomy throughout the stimulation session.

2.Structural and Functional Image Integration

High-resolution T1-weighted anatomical images acquired during the MRI session were imported into Brainsight for each participant. To guide stimulation target selection, individual anatomical images were integrated with functional connectivity maps derived from task-related fMRI analyses conducted during fear conditioning. Specifically, seed-based functional connectivity maps identifying cortical regions functionally coupled with ventromedial prefrontal cortex (vmPFC) were overlaid onto each participant's anatomical reconstruction.

Skin and full-brain curvilinear surface reconstructions were generated within Brainsight to enable precise visualization of cortical landmarks and to facilitate accurate coil placement and orientation relative to the underlying cortical target.

3.Connectivity-Informed Target Definition

Because vmPFC is a deep cortical structure not directly accessible to TMS, a connectivity-informed targeting strategy was employed to identify a surface-accessible cortical site capable of indirectly modulating vmPFC circuitry. Seed-based functional connectivity analyses were performed using fMRI data acquired during fear conditioning. A vmPFC region of interest was defined a priori, and for each participant, the vmPFC time series was correlated with voxel-wise BOLD signals across the brain to generate individual whole-brain functional connectivity maps.

Based on these maps, a target within the left posterior dorsolateral prefrontal cortex (DLPFC) was identified as the cortical region showing the strongest and most reliable

functional connectivity with vmPFC. This region was selected as the primary stimulation target with the rationale that stimulation at this site would induce secondary modulation of vmPFC through established functional network pathways relevant to fear regulation.

4.Landmark Registration and Target Localization

Standard cranial landmarks—including the left and right preauricular points, nasion, and nose tip—were identified for each participant and registered to establish accurate coregistration between head position and MRI space. Registration accuracy was verified using Brainsight's built-in validation procedure prior to stimulation.

Two stimulation sites were defined for each participant:

- Motor threshold (MT) site, localized over the hand knob region of the primary motor cortex and used to determine the individual resting motor threshold.
- DLPFC stimulation site, defined using the connectivity-informed procedure described above and targeted to induce secondary modulation of vmPFC circuitry during extinction learning.

5.Stimulation Parameters

The TMS coil was calibrated using Brainsight's tool calibration procedure prior to each session. Resting motor threshold (rMT) was determined by stimulating the motor cortex and identifying the minimum intensity that elicited visible hand muscle twitches in at least 3 out of 6 trials. Stimulation intensity for the extinction protocol was set to 100% of the individual rMT.

During extinction learning, repetitive TMS (rTMS) was delivered at:

·Frequency: 20 Hz

·Train duration: 300 ms (6 pulses per train)

·Intensity: 100% rMT

·Total number of trains: 4 (24 pulses total)

The short train duration was selected to confine neuromodulatory effects to the target trial and minimize carryover effects across trials or blocks.

A within-subject control condition was implemented by pairing rTMS with only one conditioned stimulus (CS+TMS), while the other conditioned stimulus (CS+NoTMS) and the safety cue (CS−) were presented without stimulation. Coil positioning was

continuously monitored using Brainsight's real-time feedback (bullseye coil-centric view), and stimulation was administered only when spatial error was below 1 mm. Participants were closely monitored throughout stimulation to ensure comfort and safety. Stimulation was immediately discontinued if participants reported discomfort or intolerance. All procedures adhered to established safety guidelines for TMS application.

Dynamic Coactivation Pattern Analysis

To model temporal dynamics, we trained a Gaussian hidden Markov model (HMM) on the entire dataset, which included both pre- and post-conditioning scans from all participants. Each time point was assigned to the latent state with the highest posterior probability, yielding subject-specific state sequences for all participants across all tasks and sessions. State sequences were then organized into structured dictionaries indexed by subject, task, timepoint, and diagnostic group, and used to derive dynamic metrics including occurrence rate (OCR), mean dwell time (DT), state transition probability matrix (TP), and entropy of Markov trajectories (EMT). Specifically, these indices capture distinct but related aspects of brain dynamics—how often states are engaged (OCR), how long they are sustained (DT), how they switch (TP), and how predictable or uncertain the switching process is (EMT).

Occurrence. For each participant, we quantified state occurrence as the proportion of time points assigned to each latent state. Specifically, decoded state sequences were concatenated across tasks and the number of samples in each state was divided by the participant's total number of valid samples after preprocessing, yielding a k-dimensional vector of subject-specific occupancy rates that sum to 1.

Dwell time. For each participant and task, we computed dwell time from the decoded state sequence by identifying runs of consecutive time points assigned to the same latent state. A run's DT equals its run-length in TRs (i.e., the number of consecutive samples until the state changes), including self-runs of length 1. For each participant and state, we obtained a subject-specific mean DT.

Transition probabilities. We estimated transition probabilities empirically from each participant's decoded state. For each participant, we counted all observed transitions between successive time points across tasks (including self-transitions) to form a $k \times k$ count matrix. Counts were then row-normalized so that outgoing probabilities from each "from-state" summed to 1, yielding a participant-specific transition probability (TP) matrix.

Entropy of Markov trajectories. For each participant, we derived a $k \times k$ transition probability matrix *P* from empirical counts of adjacent states in the decoded sequence. We first computed the local (row) entropy in bits for each "from-state", $h_i = -\sum_j P_{ij} log P_{ij}$. We then obtained the stationary distribution *μ* (left eigenvector for eigenvalue 1, normalized to sum to 1) and calculated the trajectory entropy matrix *H*, which quantifies the expected cumulative uncertainty along Markov trajectories from state *i* to *j*. This was implemented following standard formulations using the fundamental matrix $(I - P + A)^{-1}$, where A tiles *μ* by rows, with corrections based on the local entropy vector and the chain's entropy rate. For group-level analysis, we summarized EMT at two levels: (i) a scalar EMT per participant obtained by averaging all entries, and (ii) the full EMT matrix *H* for element-wise comparisons. Higher EMT indicates greater uncertainty/dispersion of the state-transition process. Group-level trajectory entropy differences were visualized using matrix-based bubble plots. For each state pair (i,j), the color of each cell represented the paired t-statistic comparing pre- and post-condition EMT values across participants, while circle size reflected the absolute magnitude of the statistic. Diverging colormaps centered at zero were used to encode directionality of change, with warm colors indicating increases and cool colors indicating decreases. To ensure robust visualization and avoid scaling distortions caused by extreme values, color limits were symmetrically defined based on percentile thresholds of the statistic distribution.

Finally, we computed fear learning induced changes in dynamic features (OCR, DT, TP, EMT) between pre- and post-conditioning sessions.

Predictive Modeling Using Regularized Canonical Correlation Analysis (rCCA)

We examined whether fear-learning–induced brain reorganization can predict subsequent extinction-related memory expression, as indexed by task-evoked brain

activation and behavioral measures during recall and renewal. To this end, we applied regularized canonical correlation analysis (rCCA) with a nested cross-validation framework to assess multivariate associations between learning-related changes in dynamic brain organization and later neural and behavioral outcomes.

1. Brain and Outcome Variate Definition

Two multivariate domains were defined for the rCCA analyses.

Brain organization variate (*X*).

Based on the coactivation pattern (CAP) analysis, we identified a fear-learning–related brain state that showed systematic reorganization from pre- to post-conditioning. For each participant, we computed state-specific functional connectivity (FC) matrices for this fear-related CAP using resting-state fMRI acquired before and after conditioning. Regional time series were extracted from a predefined 24-node extended threat-circuit parcellation, and pairwise Pearson correlations were used to estimate FC within the CAP-assigned frames.

For each participant, FC matrices were computed separately for the pre- and post-conditioning resting-state scans and then differenced (POST − PRE) to index fear-learning–induced connectivity reorganization. The upper triangular elements of each FC matrix (excluding the diagonal), yielding 276 unique connections, were vectorized and used as the brain organization feature set (*X*).

Memory-expression variate (*Y*).

Outcome variables were defined as task-evoked neural activation patterns during Day 3 memory recall and renewal. Activation estimates were derived from task-based fMRI using a general linear model and summarized within the same 24-node threat-circuit atlas. Separate rCCA models were constructed for recall and renewal conditions. In complementary analyses, subjective fear ratings indexing extinction memory expression were also included as behavioral outcome variables.

2. rCCA Model Specification

We fitted the rCCA model to identify linear combinations of the brain organization variate (*X*) and memory-expression variate (*Y*) that maximized their correlation. rCCA was implemented using the CCA-Zoo Python library (version 2.5.0). Compared to standard

CCA, rCCA incorporates an L2 regularization term on the covariance matrices of both datasets, enhancing numerical stability and reducing overfitting in high-dimensional feature spaces where the number of predictors exceeds the number of observations.
The regularization parameter was optimized within an inner cross-validation loop, with candidate values ranging from 0 to 1 in increments of 0.05.

3. Nested Cross-Validation Procedure

To evaluate generalization performance and avoid circularity, we implemented a nested cross-validation framework. In the outer loop, we conducted 10-fold cross-validation repeated five times. In each iteration, nine folds were used for model training and one fold for testing. Canonical weights derived from the training data were applied to the held-out test data to generate predicted canonical variate scores.
Within each outer training set, an inner 10-fold cross-validation procedure was used to select the optimal regularization parameter. Specifically, the training data were split into ten inner folds, and for each candidate regularization value, the model was trained on nine folds and evaluated on the remaining fold. This process was repeated across all inner folds, and the parameter yielding the highest average performance was selected. The model was then refit using the full outer training set and applied to the outer test set. This nested design ensured that hyperparameter optimization was strictly confined to the training data, providing an unbiased estimate of out-of-sample predictive performance.

4. Permutation Testing

Statistical significance of the predictive associations was assessed using permutation testing. Specifically, we performed 1,000 permutations in which the rows of the outcome matrix (Y) were randomly shuffled prior to cross-validation, while preserving the structure of the brain organization matrix (X). The observed mean out-of-sample canonical correlation was compared against the resulting null distribution to obtain permutation-based p-values.

5. Canonical Loadings and Neural Signature Analysis

To characterize the neural features contributing most strongly to the predictive associations, we conducted a canonical loadings analysis using cross-validated

predictions. In each fold of the outer cross-validation, canonical variate scores were generated for participants in the test set. These predicted scores were then concatenated across folds to obtain a full set of out-of-sample canonical variate estimates.

Connectivity loadings were computed as the Pearson correlation between the predicted brain-organization variate scores and each functional connectivity feature across participants. Similarly, activation loadings were computed as the Pearson correlation between predicted memory-expression variate scores and regional activation features. Features exceeding a significance threshold of $p < .01$ were visualized to delineate the neural signature linking fear-learning–induced brain reorganization to later memory expression.

Skin Conductance Response Analysis

Skin conductance responses (SCRs) were recorded continuously during the conditioning, memory recall, and renewal phases to quantify implicit psychophysiological responses to conditioned stimuli.

For each conditioned-stimulus (CS) presentation, SCR amplitude was quantified as the peak response occurring within 0–6 s following CS onset relative to the mean signal during the 2-s pre-stimulus baseline. Trial labels were determined according to the corresponding event files. During conditioning, trials were categorized as CSm, CSp1, and CSp2. Because both CSp1 and CSp2 represented conditioned threat cues, they were combined as CS+, whereas CSm was treated as CS−.

For each participant, SCR amplitudes were averaged across all presentations of CS+ and CS−, respectively, and these participant-level estimates were compared using paired-sample t-tests to evaluate successful fear acquisition.

To further evaluate extinction-related changes in implicit fear expression, trial-by-trial SCR responses during the Day 3 memory-recall phase were extracted separately for CS+TMS, CS+NoTMS, and CS−. In addition, for each participant, SCR amplitudes were averaged separately across the first four and last four recall trials within each condition, and paired-sample t-tests were performed to assess changes in conditioned autonomic responses across the recall session.

Results

Psychophysiological evidence for successful fear acquisition and extinction

To determine whether the experimental paradigm successfully induced conditioned fear learning and extinction at the implicit psychophysiological level, we examined skin conductance responses (SCRs) during both the conditioning and Day 3 memory-recall phases.

During conditioning, conditioned threat cues (CS+) elicited significantly greater autonomic responses than safety cues (CS−) during early acquisition (Supplementary Figure 1A). Specifically, participant-level SCR amplitudes averaged across CS+ presentations were significantly greater than those averaged across CS− presentations (CS+: 0.43 ± 0.54; CS−: 0.20 ± 0.23; paired t-test: $t(78) = 5.60$, $p < .001$, Cohen's $dz = 0.63$), providing psychophysiological evidence for successful fear acquisition. We additionally performed a sensitivity analysis using square-root transformed SCR amplitudes (negative values were set to zero prior to transformation). The transformed data yielded highly similar results, with significantly greater SCR responses to CS+ than CS− (CS+: 0.560 ± 0.350; CS−: 0.369 ± 0.247; paired t-test: $t(78) = 7.94$, $p < .001$, Cohen's $dz = 0.89$).

We next examined trial-by-trial SCR dynamics during the Day 3 recall session to characterize the temporal evolution of conditioned autonomic responses during extinction-memory expression (Supplementary Figure 1B).

Both CS+TMS and CS+NoTMS conditions exhibited progressively decreasing SCR amplitudes across recall trials. Consistent with these trajectories, participant-level SCR amplitudes averaged across the first four recall trials were significantly greater than those averaged across the last four recall trials for both extinction conditions (CS+TMS: $t(78) = 4.08$, $p < .001$, Cohen's $dz = 0.46$; CS+NoTMS: $t(78) = 4.00$, $p < .001$, Cohen's $dz = 0.45$); this difference was replicated when using square-root transformed SCR amplitudes (CS+TMS: $t(78) = 4.93$, $p < .001$, Cohen's $dz = 0.55$; CS+NoTMS: $t(78) = 5.96$, $p < .001$, Cohen's $dz = 0.67$), indicating that conditioned autonomic responses progressively diminished during memory recall rather than remaining stable until the final subjective assessment.

Overall, these psychophysiological findings provide convergent evidence that the experimental paradigm successfully established conditioned fear acquisition and extinction-memory expression at both the subjective and autonomic levels.

Numbers of Coactivation States

To select the optimal number of recurrent CAPs, we computed clustering solutions for k=2 through k=14 and evaluated each using two internal validity metrics: the Davies–Bouldin (DB) index and the Ray–Turi (RT) index. In Supplementary Figure 2A, the DB index, which measures the ratio of intra-cluster scatter to inter-cluster separation, reaches its minimum value at k=5. A lower DB score indicates tighter, more distinct clusters, so this nadir suggests that a five-cluster solution most effectively balances within-state cohesion against between-state separation. The RT index (Supplementary Figure 2B) similarly attains one of its lowest values at k=5, indicating optimal cluster dispersion relative to each centroid. Based on the strength of this convergence, we fixed the number of CAPs to 5 for all subsequent HMM-CAP analyses.

State Labeling based on Spatial Activation Profiles

To improve interpretability, the five coactivation states were labeled according to their dominant spatial activation profiles. Specifically, state labels were assigned based on the ROIs showing the most prominent positive and negative activation weights, following a commonly adopted approach in CAP-based studies in which states are descriptively named according to their most salient regional or network-level activation patterns. The spatial activation profiles of all five states are shown in Supplementary Figure 3.

Spatial activation profile of the fear-learning–induced brain state

To characterize the spatial configuration of the fear-learning–related brain state identified in the CAP analysis (State 1), we examined its mean activation pattern across the 24 threat-circuit regions (Supplementary Figure 4). This state exhibited a distributed activation profile spanning medial temporal, insular, cingulate, and sensorimotor regions, indicating coordinated recruitment of multiple functionally distinct components of the threat-processing system.

Ranking regions according to activation magnitude revealed that the strongest contributors were the anterior hippocampus (aHPC), posterior insula (PI), posterior hippocampus (pHPC), precentral gyrus, and basolateral amygdala (blAMY), followed by centromedial amygdala (cmAMY), postcentral gyrus, and angular gyrus. These regions collectively encompass structures implicated in fear memory encoding and contextual representation (hippocampus), threat salience and interoceptive monitoring (insula), affective and associative learning (amygdala), and motor preparation (sensorimotor cortices). The broad spatial distribution indicates that this state reflects a coordinated whole-circuit configuration rather than focal activation within any single region.

Temporal Dynamics of Dwell Time

To further characterize the temporal evolution of post-learning brain dynamics, we examined the mean dwell time (DT) of the fear-learning–related brain state across the post-conditioning resting period. Using the same sliding-window approach described in the main text, the resting-state time series were segmented into windows of varying lengths, and within each window we computed the average duration for which the fear-learning–related state was continuously maintained.

Across all temporal resolutions, DT exhibited a progressive increase as a function of time following conditioning (Supplementary Figure 5). Early windows were characterized by shorter dwell durations, whereas later windows showed systematically prolonged state persistence. This temporal profile closely paralleled the time-dependent increase observed for the occurrence rate (OCR) reported in the main results.

Importantly, whereas OCR reflects the frequency with which a state is entered, DT quantifies how long that state is sustained once engaged. The convergence of these two independent dynamic metrics therefore indicates that post-learning neural dynamics are characterized not only by more frequent recruitment of the fear-learning–related brain state, but also by greater temporal stability of that state over time. Together, these supplementary findings provide convergent evidence for a temporally graded strengthening of this state during the post-conditioning resting period.

Connectivity Reorganization of the Fear-Learning–Related Brain State

To characterize the specific connectivity architecture underlying fear-learning–induced brain reorganization, we quantified state-specific functional connectivity differences between post- and pre-conditioning resting scans for the identified fear-learning–related brain state (State 1). For each participant, connectivity change matrices were computed as the raw difference between post-conditioning and pre-conditioning connectivity (POST − PRE), without additional normalization, and these individual matrices were used as predictors in the main multivariate analyses (Supplementary Figure 6A).

To identify connections showing consistent learning-related modulation at the group level, we conducted paired t-tests for each edge across participants. Statistical thresholds were set at three significance levels ($p < .05$, $p < .01$, and $p < .005$, uncorrected) to visualize the spatial pattern of connectivity reorganization across increasingly stringent criteria (Supplementary Figure 6B–D).

Group-Level Activation Patterns During Extinction Memory Expression After TMS

To characterize the spatial distribution of neural responses during extinction memory expression, we computed group-averaged activation maps across all participants for Day 3 recall and renewal tasks under TMS-modulated extinction. For each condition, activation values were extracted for each of the 24 predefined threat-circuit regions of interest (ROIs) and averaged across participants. ROIs were then ranked according to activation magnitude, and color intensity was assigned proportionally to activation strength to visualize regional contribution profiles.

During both recall and renewal conditions, activation patterns showed a consistent spatial organization (Supplementary Figure 7). Regions exhibiting the strongest positive activation included visual cortex (VIS), anterior insula subdivisions (dAI and vAI), inferior frontal gyrus opercular part (IFGoper), and dorsal anterior cingulate cortex (dACC), indicating prominent recruitment of sensory-salience–control circuitry during extinction memory expression. In contrast, regions showing relatively reduced activation included medial prefrontal and hippocampal structures, with the ventromedial prefrontal cortex (vmPFC), anterior hippocampus (aHPC), and basolateral amygdala (blAMY) among the most suppressed regions.

Comparing recall and renewal revealed partially overlapping yet distinct activation rankings. While both conditions shared strong engagement of sensory–salience regions, renewal exhibited relatively stronger modulation of hippocampal–limbic and motor-related regions, suggesting differential neural expression profiles across extinction memory retrieval contexts.

Demographic Covariate and Sensitivity Analyses

To evaluate whether demographic variability influenced the reported brain-state findings, we performed additional linear regression analyses including age and sex as predictors of conditioning-induced changes in occupancy rate (OCR) and dwell time (DT) for the identified fear-learning–related state (State 1). Neither age nor sex was significantly associated with OCR changes (age: $\beta = 5.19 \times 10^{-5}$, $p = .939$; sex: $\beta = -0.009$, $p = .433$). Similarly, neither age nor sex was significantly associated with DT changes (age: $\beta = -0.017$, $p = .152$; sex: $\beta = 0.170$, $p = .387$). These findings indicate that the observed fear-learning–related reorganization of brain-state dynamics was not attributable to demographic variability within the sample.

To further evaluate whether age and sex influenced the multivariate prediction results, we performed an additional age- and sex-controlled sensitivity analysis for the rCCA models. Specifically, age and sex effects were removed from both the brain-reorganization features and memory-expression features within each training fold prior to model fitting, thereby preventing information leakage while accounting for potential demographic influences. The resulting predictive performance remained highly comparable to the primary analyses. Significant brain–brain prediction was observed for both recall ($r = 0.43$, permutation $p = .008$) and renewal ($r = 0.33$, permutation $p = .03$). These values closely matched the original prediction results reported in the main manuscript, indicating that the observed predictive relationships were robust to demographic adjustment.

Stability of Connectivity- and Activation-Side Neural Signatures

To evaluate the robustness of the neural signatures identified by the rCCA models, we performed a non-parametric bootstrap analysis (1000 iterations) and estimated

confidence intervals for both connectivity-side and activation-side canonical loading profiles (Supplementary Figure 8).

For the recall model (Supplementary Figure 8A), the connectivity-side neural signature showed high stability across bootstrap resamples. The strongest contributing functional connections identified in the original analysis remained among the top-ranked features following resampling, and most major connections retained loading estimates that were consistently different from zero. Similarly, the activation-side neural signature was robust, with the caudate, cerebellum, precentral gyrus, superior frontal gyrus (SFG), posterior cingulate cortex (PCC), and related regions remaining the dominant contributors across bootstrap iterations. The relative ranking of these regions was largely preserved, indicating that the recall-related activation signature was not driven by a small subset of participants.

For the renewal model (Supplementary Figure 8B), bootstrap analysis likewise demonstrated stable connectivity-side and activation-side loading profiles. Functional connections involving the orbitofrontal cortex (OFC), dorsal anterior insula (dAI), ventral anterior insula (vAI), inferior frontal gyrus (IFG), and related regions consistently exhibited the strongest loading magnitudes across resamples. On the activation side, IFG opercularis, posterior insula (PI), IFG triangularis, precentral gyrus, subgenual ACC (sgACC), and neighboring regions remained the most prominent contributors. Additionally, the ordering of the highest-loading regions and connections closely matched that observed in the original models.

Overall, the bootstrap results demonstrated that both recall- and renewal-related predictive signatures were highly reproducible across resamples. The principal connectivity and activation features identified in the primary analyses consistently remained among the strongest contributors and exhibited stable loading estimates, supporting the robustness and interpretability of the identified brain–brain predictive relationships.

Analysis of Cue-Specific Brain–Behavior Associations

To examine whether the stronger brain–brain predictive relationships observed under TMS-modulated extinction could be attributed to intrinsic differences between the two

conditioned stimuli, we separately evaluated associations between fear-learning–induced brain reorganization and behavioral outcomes for each conditioned cue across recall (CS+TMS, CS+NoTMS) and renewal (CS+TMS, CS+NoTMS) conditions (Supplementary Figure 9). Because the CS+TMS and CS+NoTMS stimuli corresponded to distinct conditioned cues acquired during Day 1 fear learning (CS+1 and CS+2), this analysis allowed us to assess whether cue identity influenced subsequent brain–behavior coupling. Although moderate-to-large correlation coefficients were observed across all four conditions (recall CS+TMS: $r(78) = 0.70$; renewal CS+TMS: $r(78)=0.71$; recall CS+NoTMS: $r(78) = 0.72$; renewal CS+NoTMS: $r(78) = 0.73$), none of these associations survived permutation-based significance testing (recall CS+TMS: $p = .93$; renewal CS+TMS: $p = .87$; recall CS+NoTMS: $p = .60$; renewal CS+NoTMS: $p = .59$). This apparent discrepancy is expected because rCCA explicitly identifies linear projections that maximize the correlation between the two variable sets; therefore, in-sample canonical correlations can be relatively high even when the association does not generalize beyond the observed sample (Supplementary Figure 9). For this reason, we relied on permutation-based inference rather than the nominal correlation magnitude alone to determine statistical significance. The magnitude of the observed associations was comparable across the two conditioned cues and across recall and renewal phases. To evaluate whether the reported correlations were disproportionately influenced by a small number of observations, we performed an additional sensitivity analysis using Cook's distance. A simple linear regression was fitted between the two canonical variate scores, and Cook's distance was computed for each participant using the fitted regression model. Following a commonly used criterion, observations with Cook's distance greater than 4/N were considered potentially influential. Pearson and Spearman correlation analyses were then repeated after excluding these observations to assess the robustness of the reported associations. Repeating the correlation analyses after excluding these observations yielded Pearson correlation coefficients that were highly similar to those obtained using the complete dataset, and all major statistical conclusions remained unchanged. (recall CS+TMS: $r = 0.67$; renewal CS+TMS: $r = 0.65$; recall CS+NoTMS: $r = 0.75$; renewal CS+NoTMS: $r = 0.75$; all $p>.05$)

As another robustness analysis, Spearman rank correlations yielded a similar pattern of associations across all four conditions (recall CS+TMS: Spearman's *r*(78) = 0.41; renewal CS+TMS: Spearman's *r*(78) = 0.71; recall CS+NoTMS: Spearman's *r*(78) = 0.53; renewal CS+NoTMS: Spearman's *r*(78) = 0.67, all p>.05), indicating that the observed relationships were not driven by assumptions of normality or linearity.

Overall, the enhanced brain–brain predictive relationships observed in the TMS-modulated extinction condition are unlikely to be explained by cue-specific differences established during fear acquisition.

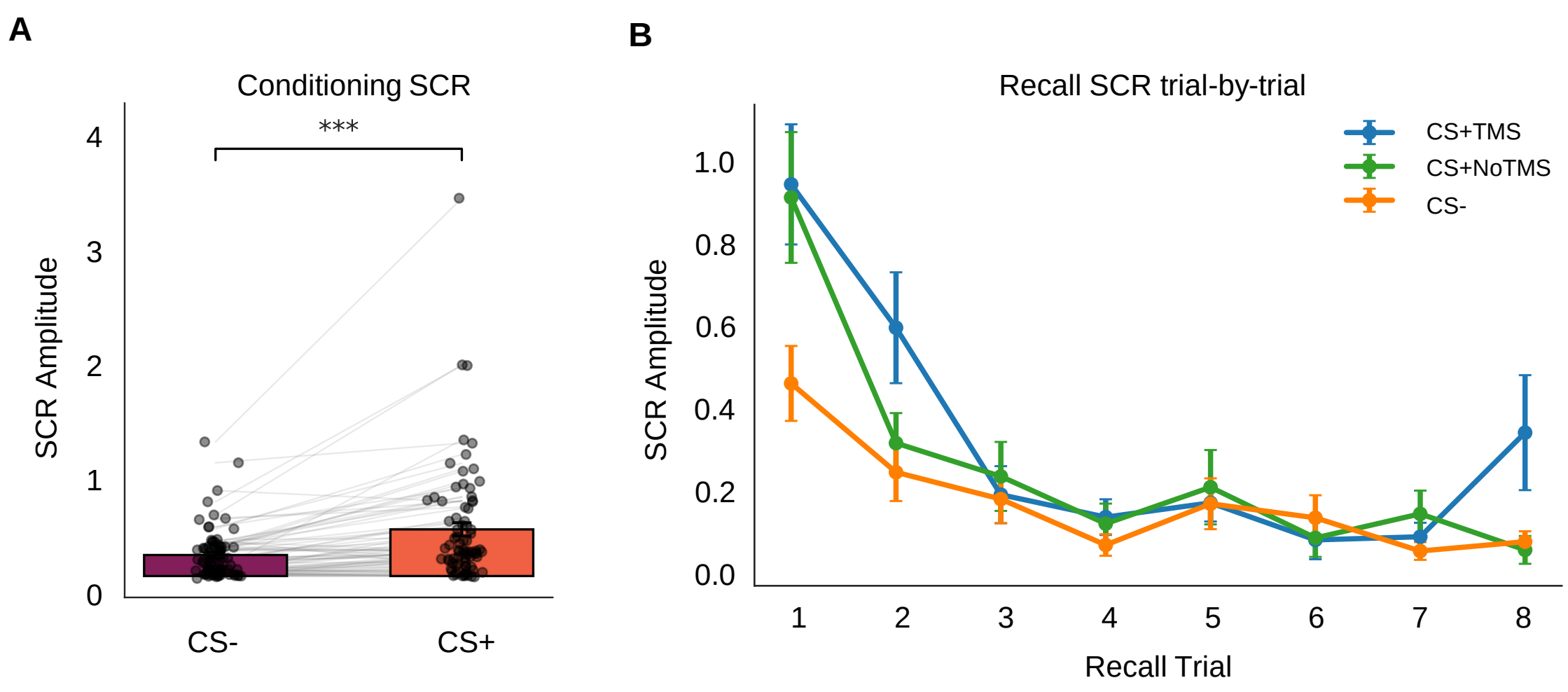

Supplementary Figure 1. Psychophysiological evidence for successful fear acquisition and extinction. (A) Comparison between CS+ and CS− during fear conditioning. Data are shown for *n* = 79 independent participants, with each dot representing one participant and lines connecting paired observations from the same participant. Participant-level SCR amplitudes were averaged across CS+ and CS− presentations and compared using a two-sided paired t-test ($t(78) = 5.60$, $p = 3.04\text{e-}07$, Cohen's *dz* = 0.63). ***$p < .001$. (B) Trial-by-trial psychophysiological dynamics during extinction-memory recall. Data are presented as mean ± SEM across *n* = 79 independent participants. SCR amplitudes averaged across the first four and last four recall trials were compared using two-sided paired t-tests for each extinction condition (CS+TMS: $t(78) = 4.08$, $p = 1.07\text{e-}04$, Cohen's *dz* = 0.46; CS+NoTMS: $t(78) = 4.00$, $p = 1.39\text{e-}04$, Cohen's *dz* = 0.45).

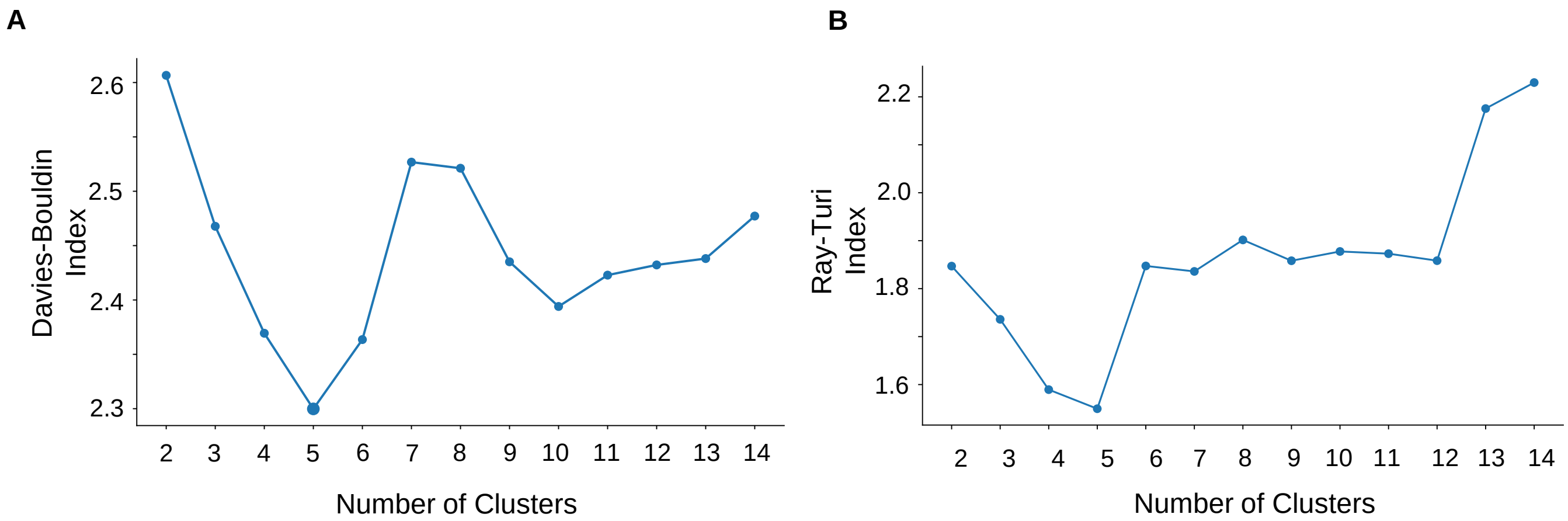

Supplementary Figure 2. Selection of the optimal number of co-activation patterns. (A) Optimal number calculated by the Davies–Bouldin Index. (B) Optimal number calculated by the Ray–Turi Index.

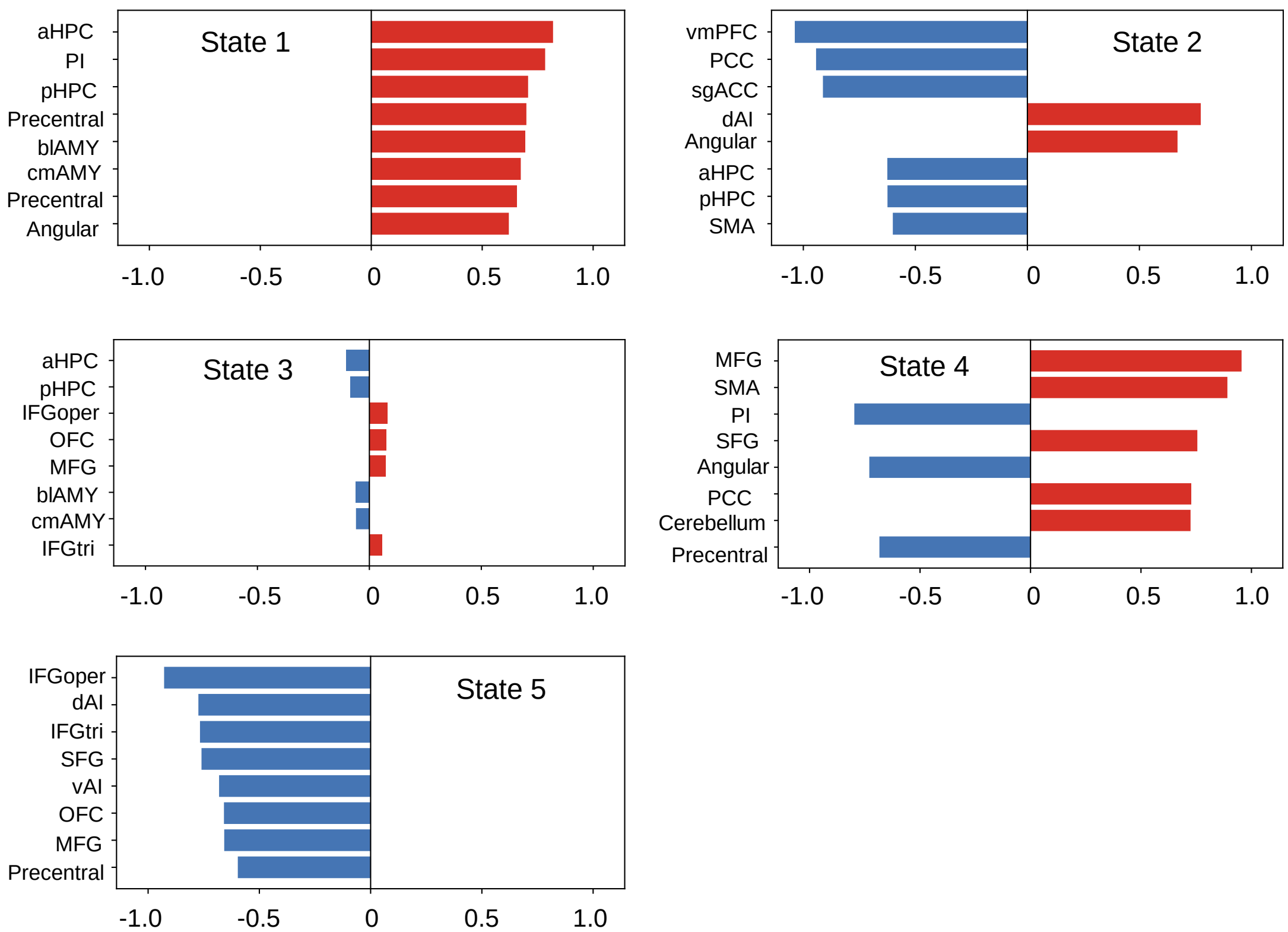

Supplementary Figure 3. Spatial activation profiles of the five coactivation states. For each state, red bars indicate the ROIs with the strongest positive activation weights and blue bars indicate the ROIs with the strongest negative activation weights.

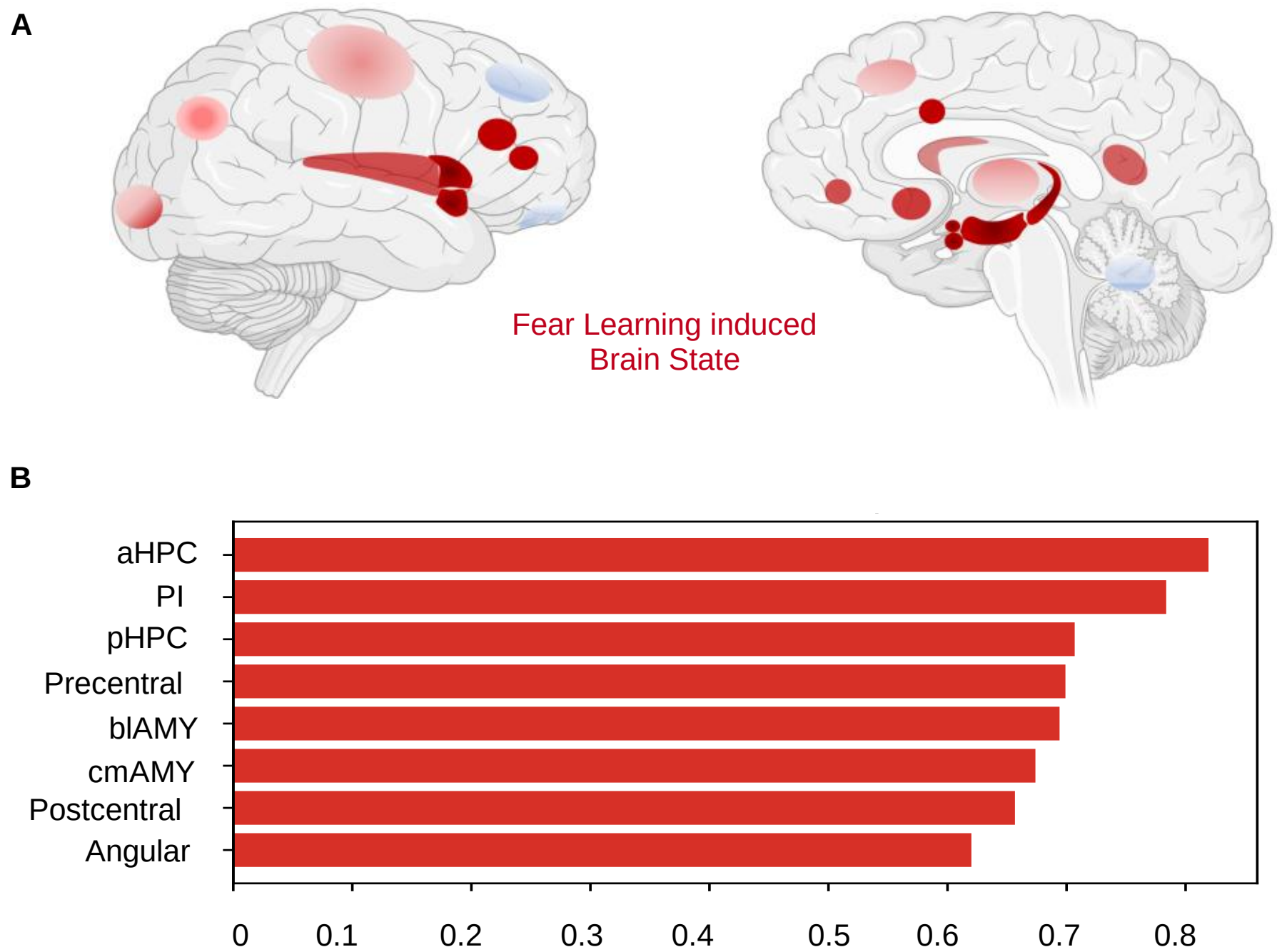

Supplementary Figure 4. Spatial activation pattern of the fear-learning–induced brain state. (A) Surface and medial views showing group-mean activation weights across the 24 threat-circuit ROIs. (B) Ranking of regional contributions based on activation magnitude. Icons were created in BioRender, with attribution lines: Zhang, K. (2026) https://BioRender.com/d8rx7ph**.**

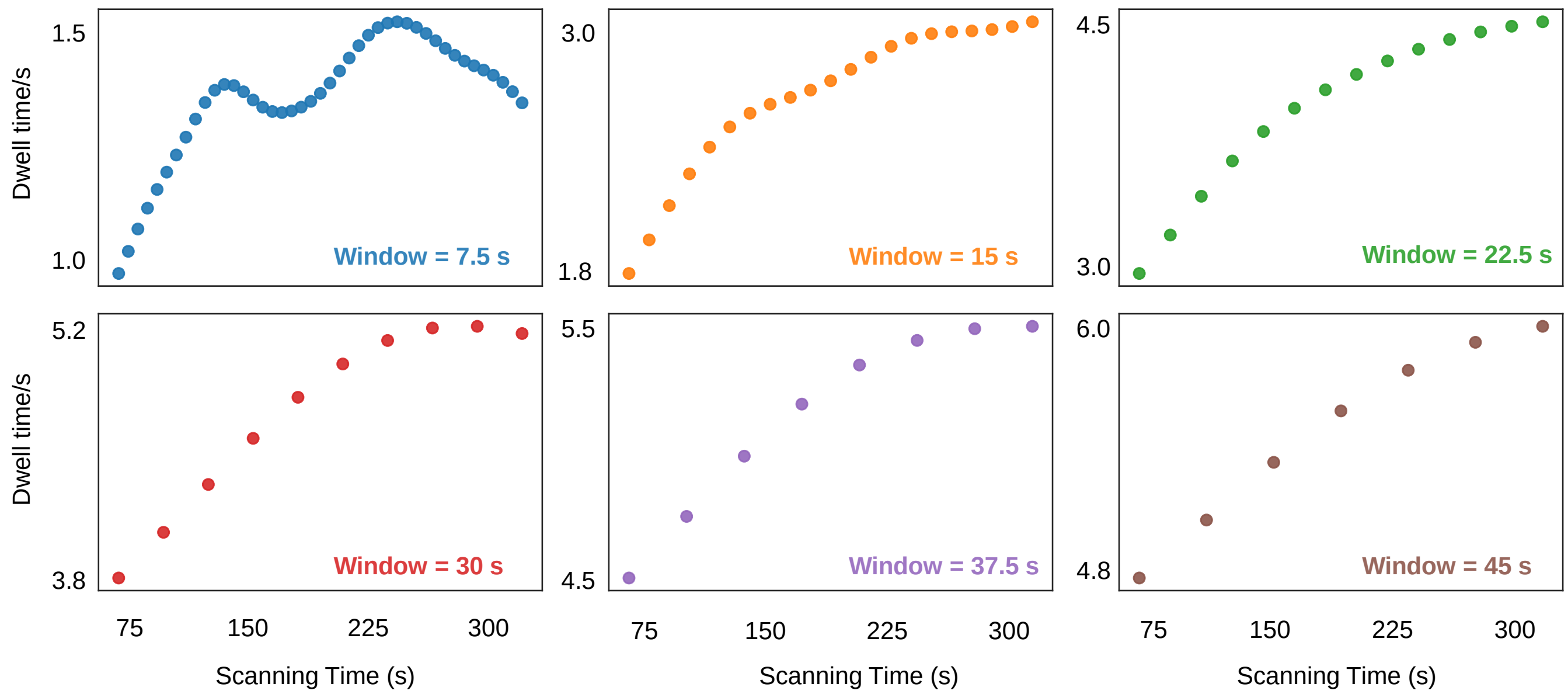

Supplementary Figure 5. Temporal tracking of fear-learning–induced brain-state engagement during post-conditioning rest.

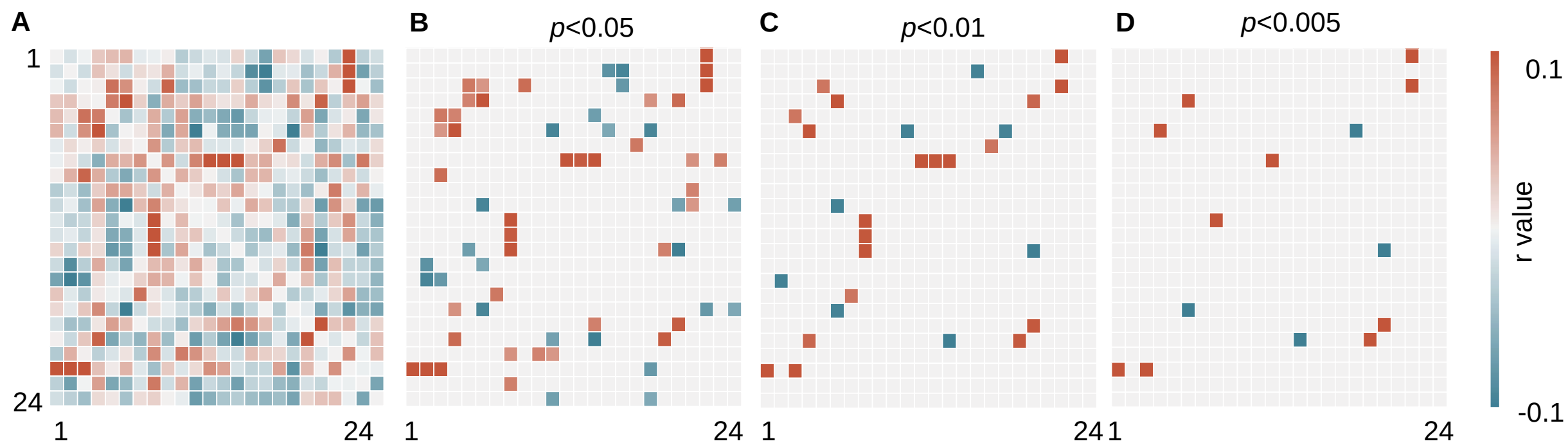

Supplementary Figure 6. Connectivity reorganization of the fear-learning–related brain state. (A) Group-level visualization of functional connectivity reorganization for the fear-learning–related brain state, computed as the raw difference between post-conditioning and pre-conditioning connectivity matrices (POST − PRE). (B-D) Group-level visualization of functional connectivity reorganization surviving $p < .05$, $p < .01$ and $p < .005$ (POST − PRE).

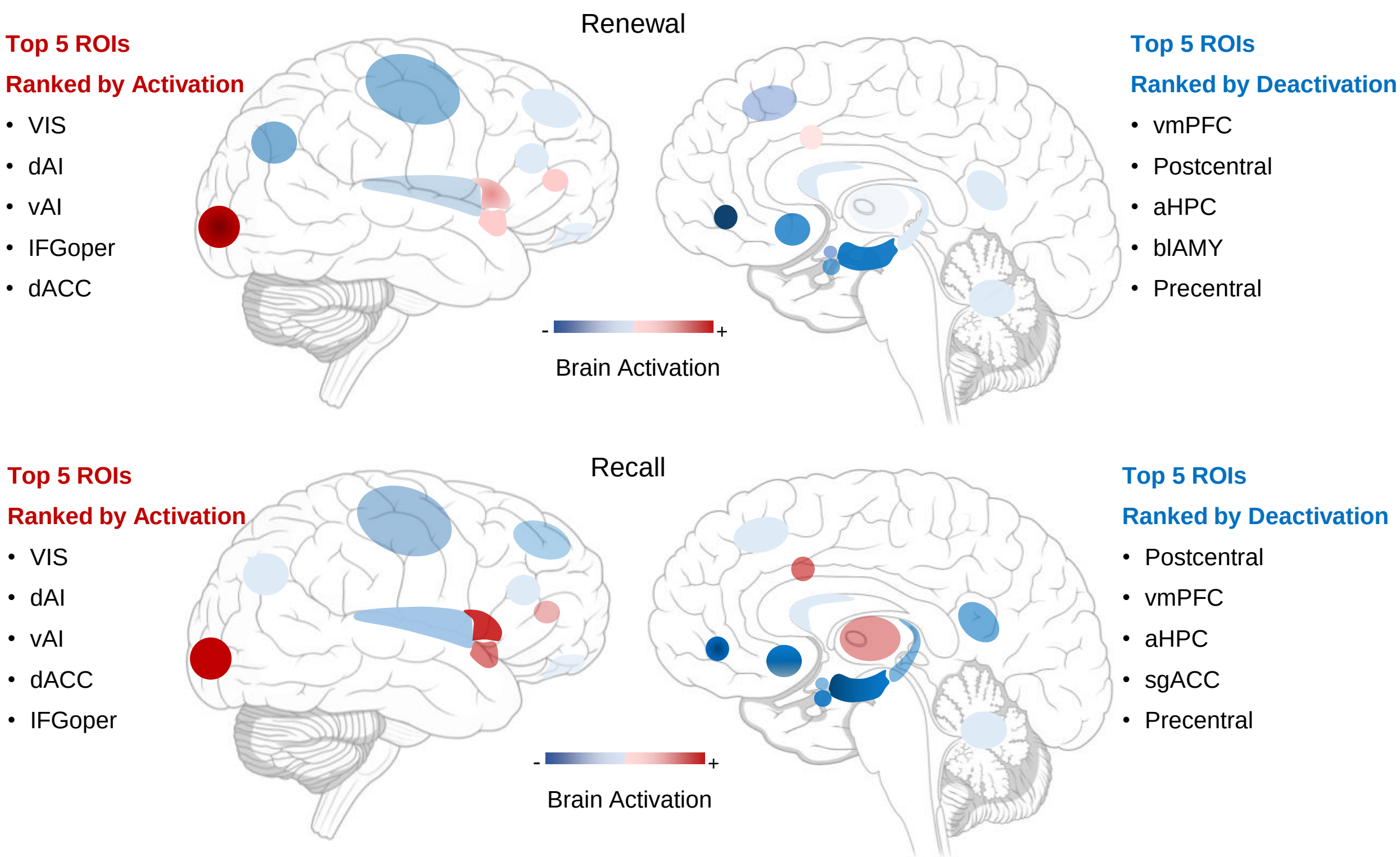

Supplementary Figure 7. Spatial distribution of mean ROI activation during extinction memory expression following TMS-modulated extinction. Top panel for renewal condition, bottom panel for recall condition. Icons were created in BioRender, with attribution lines: Zhang, K. (2026) https://BioRender.com/d8rx7ph.

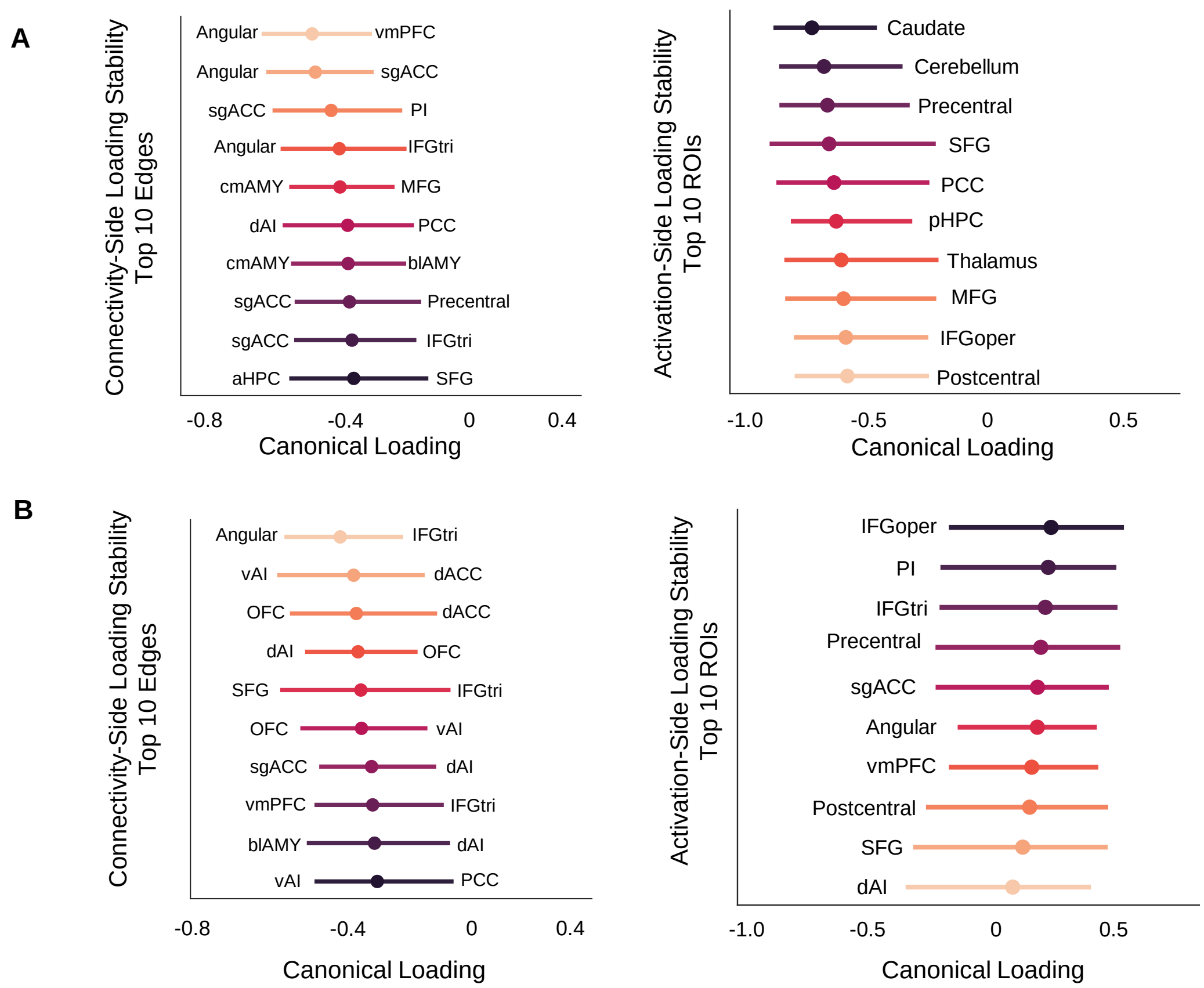

Supplementary Figure 8. Bootstrap stability of connectivity-side and activation-side neural signatures. (A) Bootstrap stability of the neural signatures identified in the extinction-memory recall prediction model. The left panel shows the top 10 connectivity edges and the right panel shows the top 10 activation ROIs ranked according to the stability of their canonical loadings across bootstrap resamples. (B) Corresponding bootstrap stability analysis for the extinction-memory renewal prediction model. Analyses were based on *n* = 80 independent participants. Bootstrap distributions were estimated using 1,000 resamples with replacement. Points indicate the mean canonical loading across bootstrap resamples, and horizontal lines indicate the 95% bootstrap confidence intervals (2.5th–97.5th percentiles). No technical replicates were included.

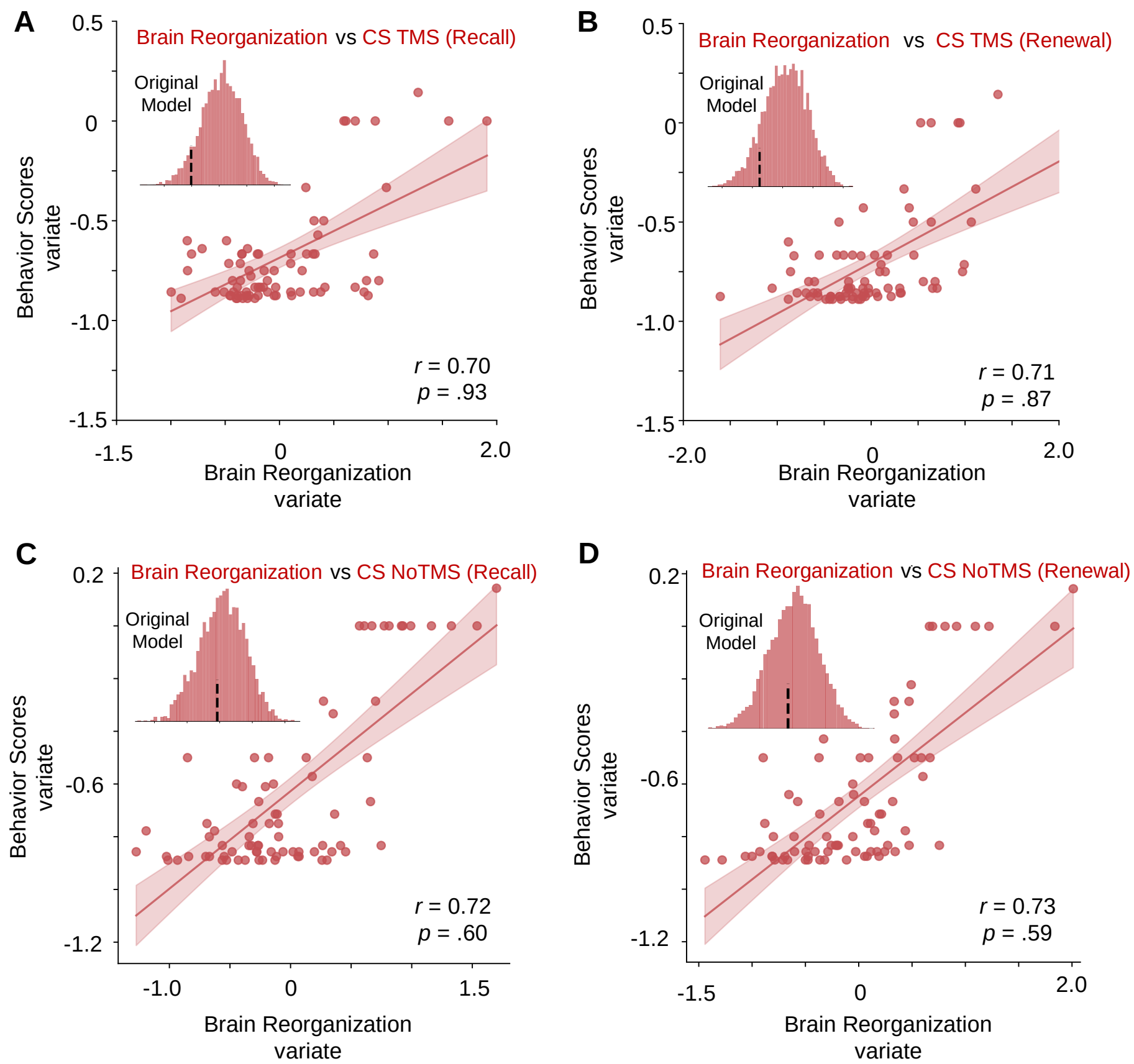

Supplementary Figure 9. Correlations between fear-learning–induced brain reorganization and behavioral scores across four experimental conditions. (A) Recall under CS+TMS; (B) Renewal under CS+TMS; (C) Recall under CS+NoTMS; (D) Renewal under CS+NoTMS. Statistical analyses were performed using data from 80 independent healthy participants (n = 80). Each data point represents one independent participant; no technical replicates were included. Insets show the null distributions generated by permutation testing, and the red dashed lines indicate the correlation coefficients obtained from the original (non-permuted) models. Brain–behavior associations were assessed using Pearson correlation. Statistical significance was determined using two-sided permutation tests (1000 permutations).
