# Supplementary material for: Fear learning–induced brain dynamics predict individual extinction memory expression following transcranial magnetic stimulation
Source: Nat Commun. 2026 Oct 5;17:11357. doi: 10.1038/s41467-026-78345-8 (PMC13728185; doi:10.1038/s41467-026-78345-8)
Supplement: Supplementary file 2 [file 41467_2026_78345_MOESM2_ESM.pdf]

nature portfolio

Corresponding author(s): Mohammed R. Milad

Last updated by author(s): Aug 7, 2026

Reporting Summary

Nature Portfolio wishes to improve the reproducibility of the work that we publish. This form provides structure for consistency and transparency in reporting. For further information on Nature Portfolio policies, see our Editorial Policies and the Editorial Policy Checklist.

Statistics

For all statistical analyses, confirm that the following items are present in the figure legend, table legend, main text, or Methods section.

n/a	Confirmed	
☐	☒	The exact sample size (*n*) for each experimental group/condition, given as a discrete number and unit of measurement
☐	☒	A statement on whether measurements were taken from distinct samples or whether the same sample was measured repeatedly
☐	☒	The statistical test(s) used AND whether they are one- or two-sided<br>*Only common tests should be described solely by name; describe more complex techniques in the Methods section.*
☐	☒	A description of all covariates tested
☐	☒	A description of any assumptions or corrections, such as tests of normality and adjustment for multiple comparisons
☐	☒	A full description of the statistical parameters including central tendency (e.g. means) or other basic estimates (e.g. regression coefficient) AND variation (e.g. standard deviation) or associated estimates of uncertainty (e.g. confidence intervals)
☐	☒	For null hypothesis testing, the test statistic (e.g. *F*, *t*, *r*) with confidence intervals, effect sizes, degrees of freedom and *P* value noted<br>*Give P values as exact values whenever suitable.*
☒	☐	For Bayesian analysis, information on the choice of priors and Markov chain Monte Carlo settings
☐	☒	For hierarchical and complex designs, identification of the appropriate level for tests and full reporting of outcomes
☐	☒	Estimates of effect sizes (e.g. Cohen's *d*, Pearson's *r*), indicating how they were calculated

Our web collection on statistics for biologists contains articles on many of the points above.

Software and code

Policy information about availability of computer code

Data collection	No specific software was used during data collection.
Data analysis	The code used for data processing and statistical analyses is publicly available through the GitHub repository and has been permanently archived in Zenodo (https://doi.org/10.5281/zenodo.21829352). The repository includes scripts for time-series extraction, functional connectivity computation, co-activation pattern (CAP) analysis (including the calculation of four CAP-related metrics), and rCCA-based prediction analyses. The implementation builds upon publicly available Python libraries including CCA-Zoo https://cca-zoo.readthedocs.io/en/latest/modules/classes.html,<br>Nilearn https://nilearn.github.io/stable/index.html for fMRI data processing, and Seaborn https://seaborn.pydata.org/ for data visualization.<br><br>NumPy v1.26.4; pandas v2.2.3; SciPy v1.11.4; scikit-learn v1.6.1; statsmodels v0.14.0; Matplotlib v3.10.1; Seaborn v0.13.2; NiBabel v5.2.1; Nilearn v0.11.1; CCA-Zoo 2.5.0; hmmlearn v0.3.3.

For manuscripts utilizing custom algorithms or software that are central to the research but not yet described in published literature, software must be made available to editors and reviewers. We strongly encourage code deposition in a community repository (e.g. GitHub). See the Nature Portfolio guidelines for submitting code & software for further information.

Data

Policy information about availability of data

All manuscripts must include a data availability statement. This statement should provide the following information, where applicable:

- Accession codes, unique identifiers, or web links for publicly available datasets
- A description of any restrictions on data availability
- For clinical datasets or third party data, please ensure that the statement adheres to our policy

Source data are provided with this paper. The data supporting the findings of this study have also been deposited in Figshare.The participant-level neuroimaging and behavioral data of this study are not publicly available because public deposition is restricted by the conditions of the institutional ethical approval and data-sharing requirements for the protection of participant privacy. Access to de-identified data may be requested from the corresponding author, Mohammed R. Milad (Mohammed.R.Milad@uth.tmc.edu). Requests should include a description of the proposed research and will be reviewed in accordance with applicable institutional and NIH data-sharing requirements. Data will be made available to qualified researchers for research purposes following approval of the request and completion of any required institutional data-use agreement.

Research involving human participants, their data, or biological material

Policy information about studies with human participants or human data. See also policy information about sex, gender (identity/presentation), and sexual orientation and race, ethnicity and racism.

Reporting on sex and gender	Sex was recorded as a biological variable (male/female) during participant enrollment. The final imaging sample included 41 males and 39 females(mean age = 28.36 ± 8.33 years, range = 18–60 years). The study was not designed or powered to investigate sex-specific effects. However, sex was evaluated as a covariate in sensitivity analyses of the CAP and rCCA models, and the primary findings remained unchanged.
Reporting on race, ethnicity, or other socially relevant groupings	Race and ethnicity data were not collected because they were not relevant to the objectives of the present study and were therefore not included in the study design or statistical analyses.
Population characteristics	Healthy adults aged 18–60 years were recruited at The University of Texas Health Science Center at Houston (UTHouston). The initial cohort comprised 87 participants (43 males and 44 females; mean age ± SD = 28.79 ± 9.20 years). Seven participants were excluded because of incomplete imaging data (missing post-conditioning resting-state scans, n = 4; missing Day 3 memory-expression imaging data, n = 3), resulting in a final sample of 80 participants (41 males and 39 females; mean age ± SD = 28.36 ± 8.33 years).Participants with neurological disorders, psychiatric disorders, or MRI contraindications were excluded.
Recruitment	Participants were recruited via advertisements posted on online platforms and public transportation advertisements.
Ethics oversight	All participants provided written informed consent in accordance with the Declaration of Helsinki and protocols approved by the Committee for the Protection of Human Subjects (CPHS), the Institutional Review Board of The University of Texas Health Science Center at Houston (UTHealth Houston).

Note that full information on the approval of the study protocol must also be provided in the manuscript.

Field-specific reporting

Please select the one below that is the best fit for your research. If you are not sure, read the appropriate sections before making your selection.

[x] Life sciences   [ ] Behavioural & social sciences   [ ] Ecological, evolutionary & environmental sciences

For a reference copy of the document with all sections, see nature.com/documents/nr-reporting-summary-flat.pdf

Life sciences study design

All studies must disclose on these points even when the disclosure is negative.

Sample size	No formal statistical method was used to predetermine sample size. The sample size was determined based on participant availability and is comparable to or larger than previous human fMRI studies combining fear conditioning, resting-state imaging, and transcranial magnetic stimulation. Eighty-seven participants were initially enrolled, and 80 participants with complete imaging data were included in the primary analyses.
Data exclusions	Participants with incomplete imaging data required for the primary analyses were excluded according to predefined quality-control criteria. Specifically, seven participants were excluded because of missing post-conditioning resting-state scans (n = 4) or missing Day 3 memory-expression imaging data (n = 3). Additional modality-specific analyses (e.g., SCR) used all available data after excluding participants with missing or technically unsuccessful recordings. No exclusions were made based on the statistical results.
Replication	Reproducibility was supported by standardized experimental, MRI acquisition, and TMS procedures applied consistently across participants; predefined preprocessing and quality-control procedures; repeated within-participant measurements across the three-day protocol; and complementary robustness analyses. Predictive analyses were evaluated using nested cross-validation and permutation testing, and the stability of the identified neural signatures was further assessed using bootstrap analyses and sensitivity analyses. Source Data and analysis code are provided to facilitate independent reproduction of the reported findings. No unsuccessful attempts to replicate the reported

	analyses were identified.
Randomization	The assignment of conditioned stimuli to the CS+TMS, CS+NoTMS, and CS− conditions was counterbalanced across participants to minimize potential stimulus-specific effects. Because all participants underwent the same experimental protocol, no random allocation to independent experimental groups was performed.
Blinding	Blinding of participants or investigators during data collection was not feasible because transcranial magnetic stimulation was administered as part of the experimental protocol. Neuroimaging preprocessing and statistical analyses were performed using standardized automated pipelines with predefined analysis procedures, minimizing potential investigator bias.

Reporting for specific materials, systems and methods

We require information from authors about some types of materials, experimental systems and methods used in many studies. Here, indicate whether each material, system or method listed is relevant to your study. If you are not sure if a list item applies to your research, read the appropriate section before selecting a response.

Materials & experimental systems

n/a	Involved in the study
☒	☐ Antibodies
☒	☐ Eukaryotic cell lines
☒	☐ Palaeontology and archaeology
☒	☐ Animals and other organisms
☒	☐ Clinical data
☒	☐ Dual use research of concern
☒	☐ Plants

Methods

n/a	Involved in the study
☒	☐ ChIP-seq
☒	☐ Flow cytometry
☐	☒ MRI-based neuroimaging

Plants

Seed stocks	NA
Novel plant genotypes	NA
Authentication	NA

Magnetic resonance imaging

Experimental design

Design type	The study combined resting-state and event-related task-based fMRI. Resting-state scans were acquired immediately before and after fear conditioning on Day 1. Event-related fMRI was acquired during fear conditioning(Day 1), fear-memory recall and renewal (Day 3) .
Design specifications	Participants completed a three-day fear-learning and extinction paradigm14 . On Day 1, participants underwent threat conditioning in which two visual cues (CS+) were paired with an aversive electric shock, whereas a third cue (CS−) was never paired with shock while fMRI data were being acquired. Resting-state fMRI was acquired immediately before and after fear conditioning to capture learning-induced changes in spontaneous brain activity. On Day 2, extinction learning was conducted without shock, during which one CS+ was paired with transcranial magnetic stimulation (TMS) delivered to the left dorsolateral prefrontal cortex (DLPFC), while the other CS+ and the CS− were presented without stimulation. On Day 3, participants completed extinction memory recall and fear renewal phases in which conditioned cues were presented without shock during extinction learning and conditioning contexts, respectively, allowing assessment of extinction memory retrieval and context-dependent fear renewal. On day 3, task-based fMRI data were collected during, extinction recall, fear renewal phases, and resting-state fMRI was acquired pre-task.
Behavioral performance measures	Behavioral performance was assessed using subjective fear ratings collected before and after fear conditioning and during memory-expression testing. Skin conductance responses (SCR) were recorded during conditioning, extinction, recall, and renewal as an objective psychophysiological index of conditioned fear. Extinction degree (ED) was calculated from subjective fear ratings to quantify individual differences in extinction-related fear reduction.

Acquisition

Imaging type(s): Blood oxygenation level-dependent (BOLD) functional MRI and high-resolution T1-weighted structural MRI

Field strength: 3 Tesla

Sequence & imaging parameters: Neuroimaging data were acquired on a 3T Siemens Prisma using a 64-channel head coil. High-resolution T1-weighted anatomical images were acquired for spatial normalization. functional images were collected with a gradient-echo echo-planar imaging (EPI) sequence (TR=1500ms; TE= 35 ms; flip angle=62°; voxel size=2×2×2mm; FOV = 208 mm; 72 slices, interleaved, multi-band acceleration factor = 4).

Area of acquisition: Whole-brain acquisition.

Diffusion MRI ☐ Used ☒ Not used

Preprocessing

Preprocessing software: Preprocessing was performed using fMRIPrep version 20.0.6, incorporating tools from ANTs (v2.3.3), FSL, AFNI, and FreeSurfer as implemented within the fMRIPrep workflow.

Normalization: Structural images were nonlinearly normalized to MNI152NLin2009cAsym space using ANTs' antsRegistration. Functional images were co-registered to the corresponding T1-weighted images and transformed into MNI space using the same nonlinear transformation.

Normalization template: MNI152NLin2009cAsym standard template.

Noise and artifact removal: Head-motion correction and slice-timing correction were performed during fMRIPrep preprocessing. For subsequent time-series extraction, nuisance regressors were generated using Nilearn's `load_confounds_strategy` with the scrubbing denoising strategy. Confounds were demeaned and standardized, and denoising included motion- and artifact-related regressors derived from fMRIPrep outputs. ROI time series were extracted using `NiftiLabelsMasker` with 6-mm spatial smoothing and temporal band-pass filtering from 0.01 to 0.08 Hz.

Volume censoring: Volume censoring was performed using Nilearn's load_confounds_strategy with denoise_strategy = "scrubbing". Volumes exceeding framewise displacement > 0.5 mm or standardized DVARS > 3 were censored using the generated sample mask.

Statistical modeling & inference

Model type and settings: The study combined several complementary statistical approaches. Dynamic brain-state metrics were compared between pre- and post-conditioning resting-state scans using paired-sample statistical analyses. Task-related brain activation was estimated using first-level general linear models (GLM), followed by group-level random-effects analyses. Predictive analyses were performed using regularized canonical correlation analysis (rCCA) with repeated nested cross-validation and permutation testing.

Effect(s) tested: Effects tested included (1) pre- versus post-conditioning differences in dynamic brain-state dynamic metric ;(2) task-related activation contrasts between conditioned threat and safety cues ; (3) multivariate prediction of extinction-memory activation from fear-learning–induced brain reorganization using rCCA; and (4) associations between neural measures and subjective fear ratings.

Specify type of analysis: ☐ Whole brain ☐ ROI-based ☒ Both

Anatomical location(s): All imaging analyses were performed within a predefined 24-node threat-circuit network

Statistic type for inference (See Eklund et al. 2016): Statistical inference was performed at the ROI level within the predefined 24-node threat-circuit network. Paired two-sided t-tests were used to compare dynamic brain-state metrics before and after conditioning. Task-related activation differences were evaluated within the predefined ROIs using general linear models (GLM), and multivariate predictive analyses were performed using regularized canonical correlation analysis (rCCA) with repeated nested cross-validation and permutation testing

Correction: Multiple comparisons across ROI-based analyses were controlled using the false discovery rate (FDR). Statistical significance of the multivariate predictive models was assessed using 1,000 permutation tests.

Models & analysis

n/a	Involved in the study
☐	☒ Functional and/or effective connectivity
☒	☐ Graph analysis
☐	☒ Multivariate modeling or predictive analysis

Functional and/or effective connectivity	Functional connectivity was estimated between all pairs of regions within the predefined 24-node threat-circuit network using Pearson correlation coefficients computed from ROI time series extracted from resting-state fMRI
Multivariate modeling and predictive analysis	Fear-learning-induced changes in functional connectivity served as predictor variables, and task-evoked activation patterns during memory recall or renewal served as outcome variables. Regularized canonical correlation analysis (rCCA) was implemented using repeated nested cross-validation (10-fold outer cross-validation repeated five times with an inner 10-fold cross-validation for hyperparameter selection). Model performance was evaluated using out-of-sample canonical correlation and statistical significance was assessed using 1,000 permutation tests.
