# Supplementary material for: Fear learning–induced brain dynamics predict individual extinction memory expression following transcranial magnetic stimulation
Source: Nat Commun. 2026 Oct 5;17:11357. doi: 10.1038/s41467-026-78345-8 (PMC13728185; doi:10.1038/s41467-026-78345-8)
Supplement: Supplementary file 3 [file 41467_2026_78345_MOESM3_ESM.pdf]

nature portfolio

Peer Review File

Fear Learning–Induced Brain Dynamics Predict Individual Extinction Memory Expression following Transcranial Magnetic Stimulation

Corresponding Author: Dr Mohammed Milad

This file contains all reviewer reports in order by version, followed by all author rebuttals in order by version.

Version 0:

Reviewer comments:

Reviewer #1

(Remarks to the Author)
In this work, Zhang and colleagues investigated the neural dynamics and the transient brain states subtending different phases of fear conditioning. In a complex fMRI-TMS experiment on healthy participants, authors disclose how different neural networks changes across different fear conditioning phases, and how the activation pattern in the acquisition phase can predict the pattern observed in subsequent phases, targeting the dlPFC cortex during the extinction session. Indeed, several interesting correlations between neural dynamics and subjective measures are reported.
I found this work incredibly interesting, and certainly lacking in the literature was a study of brain dynamics during the different phases of fear conditioning. I have no doubt that this study offers fundamental insights in this case and could be of interest to a wide range of scientists working in the field. I'm not an expert on resonance imaging, so I apologize; I sometimes found it difficult to understand certain passages. I simply have some suggestions to offer for improving an already excellent work:

Major points:
If I understand correctly, the only measure of conditioning here is subjective self-report, thus assessing only the subject's declarative memory rather than the implicit psychophysiological memory, which is often dissociated. How do the authors justify this point? How can they be sure that participants did not extinguish only at the end of the extinction session (i.e., when explicitly asked), rather than before?
The level of acquisition is also very important (Lonsdorf et al., 2017 Neuroscience & Biobehavioral Reviews), and it is not a given that the acquisition occurred at a psychophysiological level. Did the authors discard subjects who had not explicitly acquired it, the so-called learning-related non-responders?
How can authors exclude that the effect of the TMS is not related to the stimulation per sè? The lack of a control group with active stimulation in a control area is an important limitation that should be ackowlwdge.
The fact that post-acquisition brain dynamics do not predict brain patterning in subsequent sessions except when TMS is applied is not something I would expect, but rather, under natural conditions, a more "fearful" brain on day 1 would be expected to be more resistant to extinction, for example. Why is this not the case? Author should deeper discuss this point.
The participants ranged in age greatly, reaching up to 60 years old. Considering that brain changes, for example, in white matter (Fotenos et al., 2005 Neurology), occur after age 40, I think it's useful to understand why the authors chose such a large sample. Is it possible to cluster the subjects or use age as a covariate? Furthermore, how is the sample distributed by gender? A power analysis is missing.
Authors nicely show that a fear-learning induced brain state exhibited a progressive increase in engagement across the post-conditioning resting period. If I understand correctly, there has been no end to this increase. Do the authors have any idea how long this activity might continue to increase?
It would be very interesting to see how long the post conditioning dynamics last, or if these processes really last 6 hours, which is the time generally associated with memory consolidation. You may want to add this point in the discussion.
The motor cortex activation across fear conditioning phases is very interestingly, do authors believe is merely motor preparation? could you please comment on this?

Minor points
Figures are particularly hard to understand for non-experts. Could please the authors try to add more information? For

example, Figure 1 in my opinion is not very clear, in particular it is not clear which moment of the experiment the numbered state refers to, this is understood only later in the text by referring to Figure 2;
I suggest being consistent in the abstract (post conditioning or post-conditioning)
Please provide more details on how the subjective judgments were asked, were they done with VAS?

(Remarks on code availability)
Yes, the authors provided a clear README file and the uploaded codes they used for the analysis represent a usable resource for the community

Reviewer #2

(Remarks to the Author)
Comments for Transmission to the Authors

The present study addresses a genuinely important question, and the experimental design is a notable strength of the manuscript. The three-day multi-modal protocol, combining pre- and post-conditioning resting-state fMRI, individually targeted TMS during extinction, and multi-day memory recall and renewal testing, is carefully constructed and represents a methodologically ambitious human fear-learning paradigms in the recent literature. The integration of dynamic brain-state modeling with a predictive multivariate framework, and the use of the same participants across conditioning, neuromodulation, and memory expression phases, is commendable and provides a principled basis for the brain-brain predictive analyses, as well as the use of nested cross-validation scheme with permutation testing and rCCA with L2 penalization.
However, numerous concerns spanning the abstract, introduction, methods, results, and discussion require clarification before the manuscript can be accepted.

Abstract

1. Conflation of Brain Reorganization and TMS Effects (lines 25–28)
The abstract states that the study examined "how fear learning dynamically reorganizes brain activity immediately after learning, and whether such reorganization can be modulated with TMS application during extinction learning to prospectively predict extinction-memory expression." This phrasing conflates two separate processes: fear-learning-induced brain reorganization (Day 1, before TMS) and TMS applied during extinction (Day 2). The reorganization indexed by the predictive model is a Day 1 phenomenon; TMS did not modulate it. The abstract should clearly distinguish between these two processes to avoid the impression that TMS caused the brain reorganization that predicted memory.

2. Null Result Stated as a Finding (lines 40–41)
The statement "but not under natural extinction" is presented as a substantive finding. As detailed further below, this cannot be presented as a true mechanistic dissociation. It should not be stated in the abstract without qualification.

3. Outcome Variables
The abstract mentions both brain-to-brain and brain-to-behavior predictive associations in close proximity, which may create some ambiguity for readers regarding which correlations ($r = 0.47$, $r = 0.37$) refer to which outcome. A brief clarification distinguishing the two analyses more explicitly would improve readability.

Introduction

4. Methods Material in the Introduction
A substantial amount of methodological detail appears in the introduction, I understand this may be needed due to the journal structure but including descriptions of the analytic pipeline, parcellation approach, and predictive modeling framework (lines 108–121) makes it too heavy. This material should either be relocated to the Methods section or be better explained what it means, why is it of specific relevance for the paper questions?

5. Typographical Errors (lines 88, 112)
Line 88 contains the phrase "Withing this process," which should read "Within this process." Line 112 reads "TMS navigate extinction," which is grammatically incorrect and should be revised (e.g., "TMS-modulated extinction").

6. TMS Rationale Insufficiently Specified (lines 89–97)
The introduction describes TMS as "a widely used neuromodulation approach for probing the causal role of prefrontal control circuitry in fear-memory updating" but does not specify the expected direction of effect, whether stimulation was intended to enhance or suppress prefrontal activity, and by what mechanism. The introduction should state clearly whether the expected effect of DLPFC stimulation was facilitatory or inhibitory, and what the a priori prediction was regarding its effect on extinction memory. If TMS application was exploratory in nature, this should be explicitly stated and findings should be framed accordingly throughout the manuscript.

7. Transient Brain States vs. Resting-State Networks (lines 299–301)

The authors state that "human neuroimaging studies have rarely characterized how fear learning reorganizes brain dynamics at the level of transient brain states." This claim requires substantial qualification. There is an extensive literature examining resting-state network reorganization following fear conditioning using both seed-based and data-driven approaches. For example, Schultz et al. (2012) demonstrated that amygdala resting-state functional connectivity is significantly altered following Pavlovian fear conditioning, with increased amygdala-prefrontal coupling post-conditioning that correlated with behavioral fear indices, a finding directly relevant to the present studys design of acquiring resting-state fMRI before and after conditioning. Zidda et al. (2018) further showed that resting-state default mode network connectivity, specifically its coupling with the amygdala and hippocampus, is linked to individual differences in cue and context fear conditioning and predictive of trait anxiety, demonstrating that static resting-state network measures already carry meaningful individual-difference information about fear learning outcomes. Longitudinal work has further demonstrated that these alterations are not transient. Martynova et al. (2020) showed that increased resting-state functional connectivity of the left amygdala following fear conditioning persisted across immediate post-learning, 24-hour, and one-week scanning sessions in healthy participants, directly paralleling the pre-post conditioning design used here. Tambini et al. (2017) showed that amygdala-hippocampal functional connectivity increased progressively during post-acquisition and post-extinction rest, and that this persistent post-learning connectivity was stronger in participants who showed spontaneous recovery of fear 24 hours later, linking resting-state connectivity changes to subsequent memory expression in a manner that conceptually overlaps with the present papers core claim. Feng et al. (2014; 2016) further demonstrated that amygdala-dACC and hippocampus-insula resting-state connectivity were enhanced during fear memory consolidation, while amygdala-mPFC coupling was decreased, and that the change in amygdala-mPFC functional connectivity predicted individual subjective fear ratings and that resting-state functional connectivity between the amygdala and vmPFC following fear memory reactivation predicted subsequent extinction efficacy in a multi-day paradigm, again using a multi-session design comparable in structure to the present study. Given this body of literature, the authors claim of novelty cannot rest on the mere use of resting-state fMRI in the context of fear learning. The authors must clearly articulate what their coactivation pattern and hidden Markov model approach offers that prior static functional connectivity work does not, specifically how characterizing transient, moment-to-moment brain state dynamics differs from and extends prior network-level analyses. The authors do cite Marstaller et al. (2016; their ref. 41), who applied dynamic network methods to fear conditioning, which further underlines the need to more precisely situate the present contribution within the existing dynamic neuroimaging literature. Without this clarification, the framing of the papers novelty is overstated.

8. Overclaiming in the Closing Statement (lines 118–121)
The final sentence of the introduction states that the study "demonstrates that these learning-induced dynamics can be formalized as neural biomarkers that effectively predict extinction memory expression after neuromodulation." Given the absence of external validation, the inverted feature-to-sample ratio, and the modest cross-validated effect sizes, the term "effectively" overstates the strength of the evidence. This should be tempered accordingly, for example by using "may serve as" rather than "can be formalized as."

Methods

9. Discrepancy in Cross-Validation Design
The main manuscript describes a 5-fold nested cross-validation procedure, whereas the Supplementary Methods specify a 10-fold outer loop repeated five times with a 5-fold inner loop for hyperparameter selection. This is a substantive discrepancy that must be resolved. The authors should confirm which procedure was actually implemented and ensure consistency across the entire manuscript.

10. Feature-to-Sample Ratio
The predictive model ingests 276 functional connectivity features in a sample of N = 87, yielding a feature-to-sample ratio of approximately 3:1, substantially exceeding the commonly recommended minimum of 10 observations per predictor (Riley et al., 2019). While L2 regularization partially mitigates this imbalance, it does not eliminate the risk of unstable parameter estimation. The authors do not explicitly address how this inverted ratio may compromise the reliability of canonical loadings. A direct discussion of this issue, alongside evidence that the model is not capitalizing on noise, is warranted.

11. Definition and Justification of Five Coactivation States (lines 150–158)
The authors state that "Five distinct recurring patterns of spatial coactivation pattern was defined as basic brain state across all participants," a sentence that contains a grammatical error ("patterns... was") that should be corrected. More substantively, the rationale for five states is not presented in the main text. The main text should briefly describe the model selection criteria and why their convergence at k=5 was deemed sufficient. All acronyms introduced in lines 153–158 (dAI, vmPFC, sgACC, MFG, SMA, PCC) are undefined at first use and must be spelled out. Finally, the state labels appear to be assigned post-hoc based on spatial profiles, and the criteria and thresholds used to assign these interpretive labels should be made explicit.

12. TMS Rationale, Expected Direction, and Hypotheses
As noted in the introduction, it is not stated whether stimulation was intended to be inhibitory or excitatory, which circuit was expected to be modulated, or what the a priori hypotheses were. If this was an exploratory application of TMS, it should be framed explicitly as such throughout the methods, and all TMS-related findings should be interpreted as hypothesis-generating.

13. Outlier Handling and Correlation Type

The manuscript does not report whether outliers were assessed or excluded from the correlation analyses. Given the scatter visible in Figure S6, several data points appear to exert considerable leverage on the reported correlations. The authors should report: (a) whether outlier screening was performed and by what criterion; (b) whether results survive exclusion of potential outliers; and (c) whether Pearson or Spearman correlations were used and why, given that normality of canonical variate scores cannot be assumed.

14. Grammatical Error in Methods Narrative (lines 188–190)
The phrase "brain circuits to related to fear learning" (lines 188–189) contains a typographical error. More broadly, the sentence is syntactically unclear and should be rewritten for precision.

Results

15. Inconsistency Between Out-of-Sample and Supplementary Correlations
The main results report out-of-sample canonical correlations of $r = 0.47$ (recall, $p = 0.001$) and $r = 0.37$ (renewal, $p = 0.01$) for the TMS condition. However, Figure S6 in the supplement reports brain-behavior correlations of $r = 0.70–0.73$ across all four conditions, including CS+NoTMS. This magnitude gap is substantial and unexplained. The authors must clarify whether the Figure S6 values are cross-validated or in-sample estimates. In-sample correlations of $r > 0.70$ in $N = 87$ with 276 input features would constitute a serious overfitting concern and would directly undermine the cross-validation framework the paper is built upon. This is arguably the most critical inconsistency in the manuscript and must be addressed directly.

16. Result Reporting Does Not Follow Conventions (lines 269–270)
The sentence "TMS modulated extinction memory (recall: r=0.33, p=3e-3; renewal: r=0.27, p=0.01) were significantly associated with subjective fear ratings" does not follow standard reporting conventions and is grammatically incorrect. Results should be reported in standard format, e.g., $r(85) = 0.33$, $p = .003$. The subject-verb agreement error ("memory... were") should also be corrected.

17. Undefined Modulation Direction (lines 272–274)
The statement that "robust brain-behavior coupling emerged only when extinction was modulated by TMS" is insufficiently specific. The authors should state precisely in what direction and by what mechanism TMS modulated extinction-related brain activity and behavior, rather than using the uninformative term "modulated."

Discussion

18. Definition of "Fear-Related Brain State" (lines 283–286)
The discussion refers to "a fear-related brain state" as a central finding, but this label is not adequately justified. State 1 encompasses regions with broad functional roles including hippocampus, insula, and sensorimotor cortex. The authors should provide explicit justification for this label, for example by demonstrating that this states occurrence rate or dwell time correlates with conditioning-related indices, or that it is selectively engaged by CS+ versus CS- trials.

19. TMS Specificity Interpretation (lines 336–339)
The claim that the absence of prediction in the CS+NoTMS condition "suggests that naturally acquired extinction may engage a convergent, group-level neural implementation" is difficult to follow. What is meant by "convergent, group-level neural implementation," and why would such an implementation preclude individual-difference prediction? This interpretation is not self-evident and requires elaboration. Furthermore, the null result in CS+NoTMS ($r = 0.14–0.19$, $p > 0.05$) cannot be straightforwardly interpreted as evidence of a true mechanistic dissociation from the TMS condition. The issue is not the overall sample size, but rather that effect sizes of this magnitude are small enough that non-significance may reflect insufficient sensitivity to detect a weak effect rather than a true absence of one. Without a formal equivalence test specifying the smallest effect size of interest, or a power analysis targeting this specific effect size range, the observed null result in the NoTMS condition does not license the mechanistic conclusion the authors draw from it. The authors should either conduct such an analysis or substantially temper the interpretive claims made on the basis of this comparison.

General

20. Canonical Loading Stability
The manuscript reports canonical loading profiles as a primary interpretive result but provides no assessment of whether the same brain regions consistently drive the model across CV folds. A stability analysis, such as bootstrap confidence intervals or fold-by-fold correlation of loading vectors, is needed to support the neural signature interpretation.

21. Behavioral Measure
The extinction degree index relies exclusively on subjective fear ratings with no physiological corroboration (e.g., skin conductance response). This should be acknowledged as a limitation of the behavioral assessment.

Overall, the nested cross-validation architecture and permutation-based inference are genuine methodological strengths,

and the experimental design is among the more ambitious and carefully considered in the human fear-learning literature. However, the conflation of brain reorganization and TMS effects in the abstract, the absence of a clear TMS rationale and hypothesized direction, the unexplained discrepancy between out-of-sample and supplementary correlations, and several instances of imprecise or overstated interpretation all require substantive revision before the findings can be fully evaluated.

Declaration The reviewer used AI-assisted tools to support language revision of this review and to cross-check methodological details reported in the manuscript. All scientific judgments, interpretations, and recommendations are solely those of the reviewer.

(Remarks on code availability)

Version 1:

Reviewer comments:

Reviewer #1

(Remarks to the Author)
I am very satisfied with the authors' comments and appreciate the work done for the revision. I consider the manuscript now suitable for publication.

(Remarks on code availability)

Reviewer #2

(Remarks to the Author)
I am pleased with the thoroughness and quality of the authors' revisions. All major concerns have been addressed in a satisfactory manner. The addition of skin conductance response analyses, the bootstrap stability analysis for canonical loadings, the explicit clarification of in-sample versus cross-validated analyses, the direct permutation-based comparison between TMS and no-TMS predictive performance, and the substantially revised framing of the novelty claim in the introduction all represent meaningful improvements to the manuscript. The authors have also corrected all grammatical and reporting issues identified in the original review. I have no further concerns.

(Remarks on code availability)

Open Access This Peer Review File is licensed under a Creative Commons Attribution 4.0 International License, which permits use, sharing, adaptation, distribution and reproduction in any medium or format, as long as you give appropriate credit to the original author(s) and the source, provide a link to the Creative Commons license, and indicate if changes were made.

In cases where reviewers are anonymous, credit should be given to 'Anonymous Referee' and the source.
The images or other third party material in this Peer Review File are included in the article's Creative Commons license, unless indicated otherwise in a credit line to the material. If material is not included in the article's Creative Commons license and your intended use is not permitted by statutory regulation or exceeds the permitted use, you will need to obtain permission directly from the copyright holder.
To view a copy of this license, visit https://creativecommons.org/licenses/by/4.0/

Response Letter for All Comments

Responses to Reviewer #1

Comment (summary): *In this work, Zhang and colleagues investigated the neural dynamics and the transient brain states subtending different phases of fear conditioning. In a complex fMRI-TMS experiment on healthy participants, authors disclose how different neural networks changes across different fear conditioning phases, and how the activation pattern in the acquisition phase can predict the pattern observed in subsequent phases, targeting the dlPFC cortex during the extinction session. Indeed, several interesting correlations between neural dynamics and subjective measures are reported. I found this work incredibly interesting, and certainly lacking in the literature was a study of brain dynamics during the different phases of fear conditioning. I have no doubt that this study offers fundamental insights in this case and could be of interest to a wide range of scientists working in the field. I'm not an expert on resonance imaging, so I apologize; I sometimes found it difficult to understand certain passages. I simply have some suggestions to offer for improving an already excellent work.*

Response: We sincerely thank the reviewer for the thoughtful and highly encouraging assessment of our work. We greatly appreciate the reviewer's recognition of the novelty and potential impact of our study, as well as the constructive suggestions for improving the clarity, interpretation, and presentation of the manuscript.

In response to the reviewer's comments, we have carefully revised the manuscript throughout. Please see our point-to-point response to your comments below.

Major points:

Comment 1. *If I understand correctly, the only measure of conditioning here is subjective self-report, thus assessing only the subject's declarative memory rather than the implicit psychophysiological memory, which is often dissociated. How do the authors justify this point? How can they be sure that participants did not extinguish only at the end of the extinction session (i.e., when explicitly asked), rather than before?*

Response: We thank the reviewer for this important comment. We agree that subjective expectancy ratings primarily assess the declarative component of fear learning and do not directly capture implicit psychophysiological memory processes. Our justification here is that we aim to translate these findings to the clinic in prospective studies. And as such,

we believe that subjective reports would be most fit to analyze. But agree that it is important to demonstrate that extinction-related changes were not restricted to participants' explicit reports at the end of the session.

Although not reported in the original manuscript, skin conductance responses (SCRs) were acquired continuously throughout the conditioning, extinction-memory recall, and renewal phases. In response to the reviewer's suggestion, we performed additional psychophysiological analyses and have now included these results in the revised manuscript and Supplementary Materials. Of the 80 participants included in the primary neuroimaging analyses, SCR data from one participant were unavailable because of a recording failure during physiological acquisition. Consequently, all SCR analyses were conducted among the remaining 79 participants.

This analysis revealed significantly greater SCR responses to CS+ relative to CS− during conditioning (CS+: 0.43 ± 0.54; CS−: 0.20 ± 0.23; $t(78) = 5.60$, $p < .001$, Cohen's dz = 0.63), confirming successful conditioned fear acquisition at the psychophysiological level (figure below). We additionally performed a sensitivity analysis using square-root transformed SCR amplitudes (negative values were set to zero prior to transformation). The transformed data yielded highly similar results, with significantly greater SCR responses to CS+ than CS− (CS+: 0.560 ± 0.350; CS−: 0.369 ± 0.247; $t(78) = 7.94$, $p < .001$, Cohen's dz = 0.89). These findings demonstrate that fear learning was expressed not only in subjective reports but also in implicit autonomic responses.

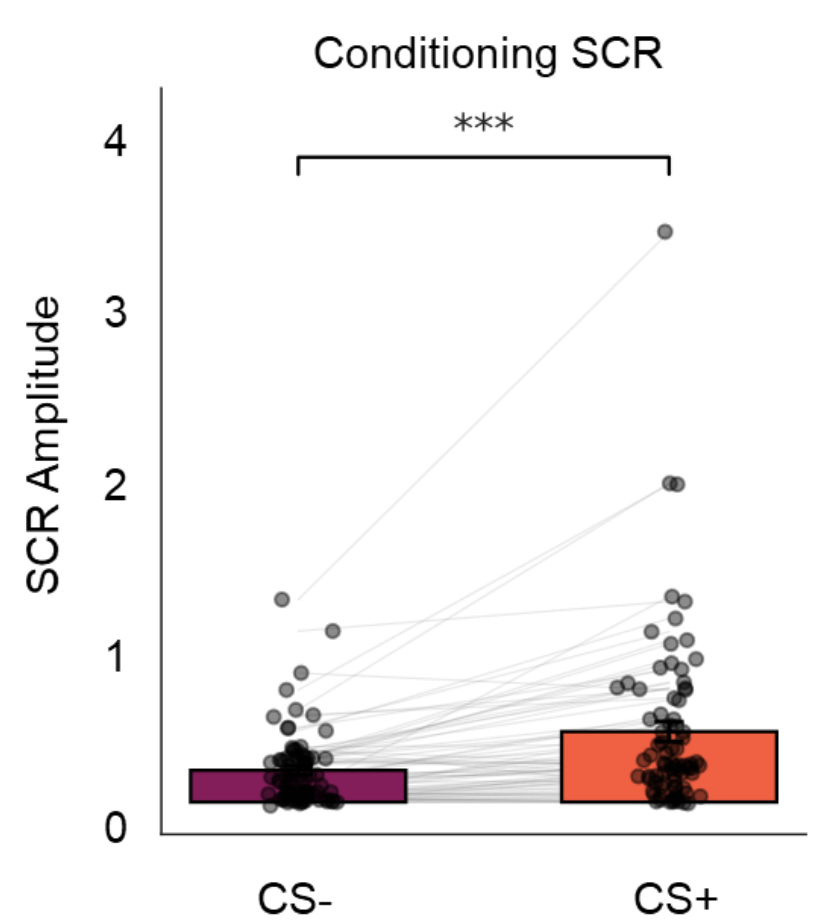

Figure S1A. Comparison of SCR amplitude between CS+ and CS- during fear conditioning

To further address the reviewer's concern regarding extinction, we examined trial-by-trial SCR responses during the Day 3 memory-recall phase. For each condition (CS+TMS, CS+NoTMS, and CS−), we compared SCR responses during the first four versus last four recall trials. Both conditioned threat cues exhibited significant reductions across the recall session (CS+TMS: $t(78) = 4.08$, $p < .001$, Cohen's dz = 0.46; CS+NoTMS: $t(78) = 4.00$, $p < .001$, Cohen's dz = 0.45). And square-root transformed SCR measuring replicate this

difference. Trial-by-trial trajectories similarly demonstrated a progressive decline in SCR responses across recall trials.

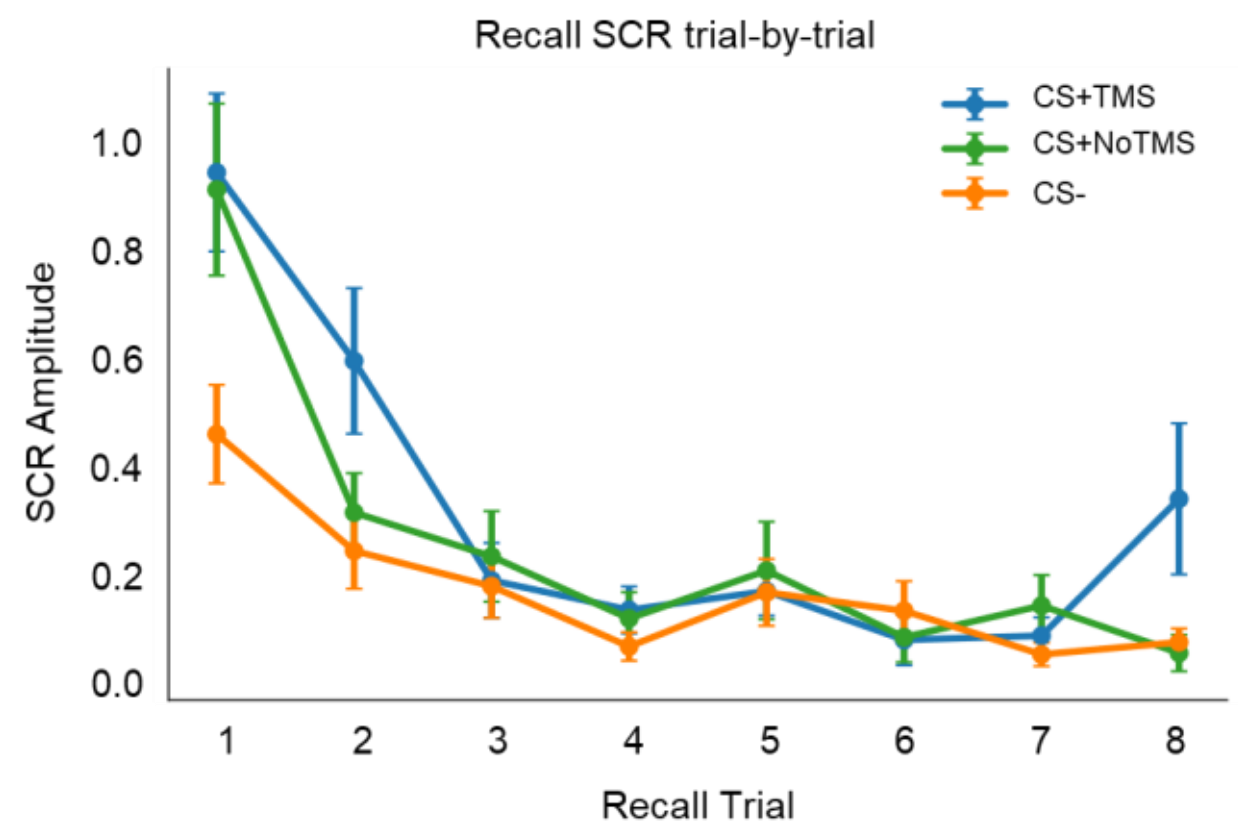

Figure S1B. Trial-by-trial psychophysiological dynamics during extinction-memory recall.

In summary, these additional psychophysiological analyses indicate that (1) conditioned fear acquisition was successfully established at the implicit autonomic level, and (2) extinction-related reductions in fear expression were already evident throughout the recall session rather than emerging only at the final explicit rating assessment. Importantly, while these additional SCR analyses provide independent psychophysiological evidence for successful fear acquisition and extinction, the primary objective of the present study was not to characterize the temporal dynamics of psychophysiological extinction itself. Rather, our central aim was to determine whether fear-learning–induced reorganization of resting-state brain networks predicts subsequent neural expression during extinction-memory recall and renewal. The newly added SCR analyses therefore serve to verify that conditioning and extinction occurred as expected, thereby strengthening the interpretation of the subsequent neuroimaging findings rather than constituting the primary outcome of the study.

In response to this comment, we have supplied this analysis into raw manuscript. Specifically:

We have added a description of skin conductance response acquisition to the Methods in main text as follows:

> "Skin conductance responses (SCRs) were also continuously acquired during the conditioning, memory recall, and renewal phases to provide an implicit psychophysiological measure of conditioned fear expression. Detailed SCR acquisition, preprocessing, and statistical analyses are described in the Supplementary Methods. (Main text, p25, line,649)"

We have added a detailed description of SCR preprocessing, quantification, and statistical analyses to the Supplementary Methods as follows:

“Skin conductance responses (SCRs) were recorded continuously during the conditioning, memory recall, and renewal phases to quantify implicit psychophysiological responses to conditioned stimuli.

For each conditioned-stimulus (CS) presentation, SCR amplitude was quantified as the peak response occurring within 0–6 s following CS onset relative to the mean signal during the 2-s pre-stimulus baseline. Trial labels were determined according to the corresponding event files. During conditioning, trials were categorized as CSm, CSp1, and CSp2. Because both CSp1 and CSp2 represented conditioned threat cues, they were combined as CS+, whereas CSm was treated as CS−.

For each participant, SCR amplitudes were averaged across all presentations of CS+ and CS−, respectively, and these participant-level estimates were compared using paired-sample t-tests to evaluate successful fear acquisition.

To further evaluate extinction-related changes in implicit fear expression, trial-by-trial SCR responses during the Day 3 memory-recall phase were extracted separately for CS+TMS, CS+NoTMS, and CS−. In addition, for each participant, SCR amplitudes were averaged separately across the first four and last four recall trials within each condition, and paired-sample t-tests were performed to assess changes in conditioned autonomic responses across the recall session. (Supplement, p8, line,239)”

We also included the SCR analyses and corresponding statistical results as follows:

“To determine whether the experimental paradigm successfully induced conditioned fear learning and extinction at the implicit psychophysiological level, we examined skin conductance responses (SCRs) during both the conditioning and Day 3 memory-recall phases.

During conditioning, conditioned threat cues (CS+) elicited significantly greater autonomic responses than safety cues (CS−) during early acquisition (Figure S1A). Specifically, participant-level SCR amplitudes averaged across CS+ presentations were significantly greater than those averaged across CS− presentations (CS+: 0.43 ± 0.54; CS−: 0.20 ± 0.23; paired t-test: $t(78) = 5.60$, $p < .001$, Cohen's dz = 0.63), providing psychophysiological evidence for successful fear acquisition. We additionally performed a sensitivity analysis using square-root transformed SCR amplitudes (negative values were set to zero prior to transformation). The transformed data yielded highly similar results, with significantly greater SCR responses to CS+ than CS− (CS+: 0.560 ± 0.350; CS−: 0.369 ± 0.247; paired t-test: $t(78) = 7.94$, $p < .001$, Cohen's dz = 0.89).

We next examined trial-by-trial SCR dynamics during the Day 3 recall session to characterize the temporal evolution of conditioned autonomic responses during extinction-memory expression (Fig. S1B).

Both CS+TMS and CS+NoTMS conditions exhibited progressively decreasing SCR amplitudes across recall trials. Consistent with these trajectories, participant-level SCR amplitudes averaged across the first four recall trials were significantly greater than those averaged across the last four recall trials for both extinction conditions (CS+TMS: $t(78) = 4.08$, $p < .001$, Cohen's dz = 0.46; CS+NoTMS: $t(78) = 4.00$, $p < .001$, Cohen's dz = 0.45); this difference was replicated when using square-root transformed SCR amplitudes (CS+TMS: $t(78) = 4.93$, $p < .001$, Cohen's dz = 0.55; CS+NoTMS: $t(78) = 5.96$, $p < .001$, Cohen's dz = 0.67), indicating that conditioned autonomic responses progressively diminished during memory recall rather than remaining stable until the final subjective assessment.

Above all, these psychophysiological findings provide convergent evidence that the experimental paradigm successfully established conditioned fear acquisition and extinction-memory expression at both the subjective and autonomic levels. (Supplement, p9, line,272)"

Comment 2. *The level of acquisition is also very important (Lonsdorf et al., 2017 Neuroscience & Biobehavioral Reviews), and it is not a given that the acquisition occurred at a psychophysiological level. Did the authors discard subjects who had not explicitly acquired it, the so-called learning-related non-responders?*

Response: We thank the reviewer for raising this important methodological point. We agree that individual differences in fear acquisition should be carefully considered in fear-conditioning studies. We in fact published our own study (Marin et al 2016) on this topic as well and highlighted the need to not exclude any participants. And in our analyses, both in those reported in the original study, and in those pertaining to the SCR analyses we added to respond to comment 1 above, we did not exclude any participant from our analyses. As described above, consistent with the subjective fear ratings reported in the main manuscript, SCR responses to CS+ were significantly greater than those to CS− during early acquisition ($t(78)=5.60$, $p<.001$), providing convergent evidence that conditioned fear learning was successfully established at both the declarative and implicit psychophysiological levels- without the exclusion of any participants.

Comment 3. *How can authors exclude that the effect of the TMS is not related to the stimulation per sè? The lack of a control group with active stimulation in a control area is an important limitation that should be acknowlwdge.*

Response: We can not exclude the TMS effect- the reviewer is correct. We agree that the absence of an active stimulation control site is an important limitation of the present study.

We have therefore revised the manuscript to avoid overinterpreting the TMS-related findings as direct evidence of site-specific modulation of the DLPFC-vmPFC pathway. Instead, we describe these effects more conservatively as arising under the TMS-modulated extinction condition. We also added this limitation to the Discussion, noting that future studies should include active control stimulation sites, such as vertex, visual cortex, or off-target prefrontal regions, to distinguish target-specific neuromodulatory effects from nonspecific effects of stimulation.

In response to this concern, we add the related state into revised main text, discussion section as follows:

> "Third, although our stimulation protocol was designed as a facilitatory neuromodulation intervention targeting the DLPFC-vmPFC regulatory circuit, our experimental protocol did not include sham stimulation or active stimulation over a control region. Therefore, although the observed predictive relationship was significantly stronger under the TMS-modulated condition within the present design, we cannot determine whether this finding reflects modulation of the targeted DLPFC-vmPFC circuit or nonspecific effects of active stimulation. A more mechanistic understanding of this effect will require future studies incorporating sham stimulation, active control stimulation sites, individualized electric-field modeling, on-target versus off-target stimulation comparisons, and multimodal neuroimaging approaches capable of directly characterizing stimulation-induced changes in large-scale brain networks. (Main text, p19, line,478)"

Comment 4. *The fact that post-acquisition brain dynamics do not predict brain patterning in subsequent sessions except when TMS is applied is not something I would expect, but rather, under natural conditions, a more "fearful" brain on day 1 would be expected to be more resistant to extinction, for example. Why is this not the case? Author should deeper discuss this point.*

Response: We thank the reviewer for highlighting this theoretically important finding. We agree that stronger post-acquisition fear expression might be expected to predict greater resistance to subsequent extinction. However, the Day 1 measure examined here should not be interpreted as an index of a "more fearful" brain. It reflects conditioning-induced reorganization of functional connectivity within a dynamically defined threat-circuit state and may capture associative encoding, post-learning plasticity, and reconfiguration of threat and regulatory systems rather than fear magnitude itself. Its relationship with later extinction-memory expression is therefore not necessarily unidirectional.

One possible explanation is that stimulation during extinction interacted with the neural configuration established after conditioning, thereby making subsequent memory-related activation more dependent on pre-existing individual differences. In this sense, TMS may have revealed variability in the mapping between Day 1 network organization and Day 3

brain activation, rather than necessarily producing stronger extinction. By contrast, extinction without stimulation may depend on more heterogeneous or compensatory neural processes that were not captured by this specific Day 1 connectivity measure. The limited number of extinction trials may also have reduced the stability of this association.

Importantly, the predicted outcome was a multivariate pattern of brain activation during recall and renewal, not a direct behavioral measure of extinction resistance. Therefore, the absence of significant prediction in the no-TMS condition does not indicate that Day 1 brain organization is unrelated to extinction without stimulation. It only indicates that the specific Day 1 neural signature examined here did not reliably predict the corresponding Day 3 activation pattern.

We also acknowledge that a significant association in the TMS-paired condition and a nonsignificant association in the no-TMS condition do not, by themselves, establish a significant difference between conditions. Moreover, because the study lacked sham stimulation and an active control site, the present findings cannot determine whether this pattern reflects DLPFC-specific modulation or nonspecific effects of stimulation. We have revised the Discussion to present this result as an unexpected and hypothesis-generating observation.

Now, we supply the related state into revised main text, discussion section as follows:

> "Notably, although both CS+TMS and CS+NoTMS conditions exhibited fear extinction, only extinction parried TMS did fear-learning–induced brain reorganization predict later recall and renewal activations. This dissociation may reflect an important property of neuromodulation. A growing body of literature suggests that focal brain stimulation does not remain restricted to the stimulated cortical site, but propagates through distributed large-scale functional networks. Such perturbations have been shown to vary substantially across individuals, contributing to considerable heterogeneity in both neural and behavioral responses to TMS. Within this framework, TMS may alter the neural implementation of extinction learning relative to natural extinction. Rather than affecting all participants uniformly, stimulation may allow pre-existing individual differences in fear-learning–related brain organization to exert a stronger influence on subsequent memory-expression processes, thereby increasing the detectability of brain–brain predictive relationships. Meanwhile, the absence of significant prediction in the no-TMS condition does not indicate that Day 1 brain organization is unrelated to extinction without stimulation. It only indicates that the specific Day 1 neural signature examined here did not reliably predict the corresponding Day 3 activation pattern. (Main text, p17, line,411)"

Comment 5. *The participants ranged in age greatly, reaching up to 60 years old. Considering that brain changes, for example, in white matter (Fotenos et al., 2005*

Neurology), occur after age 40, I think it's useful to understand why the authors chose such a large sample. Is it possible to cluster the subjects or use age as a covariate? Furthermore, how is the sample distributed by gender? A power analysis is missing.

Response: We thank the reviewer for this important and constructive comment. Firstly, the inclusion of a broad range of age is something that we feel necessary for inclusion purposes, and to enhance generalizability of our findings; such reasons are consistent with our funding agency; the NIMH. We do agree that this approach increases variance and matters related to aging that would be difficult to control for. In response, we have substantially expanded the manuscript by (1) providing detailed demographic information, including age and sex distributions; (2) evaluating the potential influence of age and sex on the primary brain-state findings; (3) conducting additional age- and sex-controlled sensitivity analyses for the rCCA prediction models.

First, we added a detailed description of the sample demographics in the revised manuscript. 87 healthy participants (43 males and 44 females) with an age range of 18–60 years (mean ± SD = 28.79 ± 9.20 years) completed a Pavlovian fear-conditioning paradigm. Because the study involved multiple sessions across three consecutive days, some data loss occurred due to missing imaging acquisitions. Specifically, four participants did not complete the post-conditioning resting-state scan, and three participants were missing Day 3 memory-expression imaging data. To ensure complete data availability across all analyses, these participants were excluded from subsequent analyses, resulting in a final sample of 80 participants included in the study. The final sample comprised 41 males and 39 females, with a mean age of 28.36 ± 8.33 years (range: 18–60 years).

We add the related state in revised main text, Method section as follows:

> "Eighty-seven healthy adults (43 males and 44 females) with an age range of 18–60 years (mean ± SD = 28.79 ± 9.20 years) were recruited at The University of Texas Health Science Center at Houston (UTHouston). Exclusion criteria included any neurological, psychiatric, or MRI contraindications. All participants provided written informed consent in accordance with the Declaration of Helsinki and protocols approved by the Institutional Review Board of UTHouston. Seven participants were excluded from subsequent analyses because of incomplete imaging data (missing post-conditioning resting-state scans, n = 4; missing Day 3 memory-expression imaging data, n = 3), resulting in a final sample of 80 participants (41 males, 39 females; mean age = 28.36 ± 8.33 years, range = 18–60 years). (Main text, p20, line,507)"

In response to the reviewer's question regarding the relatively broad age range, our intention was to recruit a sample that better reflects the demographic heterogeneity commonly observed in populations targeted by fear-extinction–based interventions and neuromodulation approaches. Importantly, although the age range extended beyond 40

years, only 6 of the 80 participants included in the final analyses were older than 40 years of age. Thus, the sample was predominantly composed of younger adults, reducing the likelihood that age-related neural changes in older individuals disproportionately influenced the reported findings. We nevertheless performed additional analyses incorporating age as a continuous covariate to formally evaluate potential demographic influences.

To address the reviewer's suggestion regarding covariate control, we performed two complementary analyses.

First, because the primary findings of the CAP analysis were driven by conditioning-induced changes in occupancy rate (OCR) and dwell time (DT) of the identified fear-learning–related state, we examined whether age and sex significantly influenced these measures. Specifically, we fitted linear regression models with age and sex as predictors of OCR and DT changes. Neither age nor sex significantly associated OCR changes (age: $\beta = 5.19 \times 10^{-5}$, p = .939; sex: $\beta = -0.009$, p = .433). Similarly, neither age nor sex significantly associated DT changes (age: $\beta = -0.017$, p = .152; sex: $\beta = 0.170$, p = .387). These findings indicate that the reported conditioning-related brain-state reorganization was not driven by demographic variability.

Second, we performed an additional age- and sex-controlled sensitivity analysis for the rCCA prediction models. Specifically, age and sex effects were removed from both the brain-reorganization features and memory-expression features within each training fold prior to model fitting, thereby preventing information leakage while accounting for potential demographic influences. Importantly, the resulting predictive performance remained highly comparable to the original analyses, with significant brain–brain prediction observed for both recall ($r = 0.43$, $p = .008$) and renewal ($r = 0.33$, $p = .03$). These values closely matched the original findings reported in the manuscript, indicating that the observed predictive relationships were robust and not attributable to age- or sex-related differences across participants.

For the concern "*A power analysis is missing*", We acknowledge that no formal a priori power analysis was performed when determining the sample size for the present study. Nevertheless, the final sample size of the present study (N = 87 recruited; N = 80 included in the primary neuroimaging analyses after exclusion because of missing imaging data) is comparable to, and in many cases larger than, those reported in previous multimodal human fear-conditioning studies combining fMRI, resting-state imaging, and TMS-based neuromodulation. We therefore believe that the present sample size is consistent with current practice in this field while recognizing that larger independent cohorts will be important for future validation.

We add all these important results into revised Supplement as follows:

"**Demographic Covariate and Sensitivity Analyses**

To evaluate whether demographic variability influenced the reported brain-state findings, we performed additional linear regression analyses including age and sex as predictors of conditioning-induced changes in occupancy rate (OCR) and dwell time (DT) for the identified fear-learning–related state (State 1). Neither age nor sex significantly associated OCR changes (age: $\beta = 5.19 \times 10^{-5}$, $p = .939$; sex: $\beta = -0.009$, $p = .433$). Similarly, neither age nor sex significantly associated DT changes (age: $\beta = -0.017$, $p = .152$; sex: $\beta = 0.170$, $p = .387$). These findings indicate that the observed fear-learning–related reorganization of brain-state dynamics was not attributable to demographic variability within the sample.

To further evaluate whether age and sex influenced the multivariate prediction results, we performed an additional age- and sex-controlled sensitivity analysis for the rCCA models. Specifically, age and sex effects were removed from both the brain-reorganization features and memory-expression features within each training fold prior to model fitting, thereby preventing information leakage while accounting for potential demographic influences. The resulting predictive performance remained highly comparable to the primary analyses. Significant brain–brain prediction was observed for both recall ($r = 0.43$, permutation $p = .008$) and renewal ($r = 0.33$, permutation $p = .03$). These values closely matched the original prediction results reported in the main manuscript, indicating that the observed predictive relationships were robust to demographic adjustment. (Supplement, p9, line,413)"

Comment 6. *Authors nicely show that a fear-learning induced brain state exhibited a progressive increase in engagement across the post-conditioning resting period. If I understand correctly, there has been no end to this increase. Do the authors have any idea how long this activity might continue to increase? It would be very interesting to see how long the post conditioning dynamics last, or if these processes really last 6 hours, which is the time generally associated with memory consolidation. You may want to add this point in the discussion.*

Response: This is really a great point and we wish we have had more time post-learning to be able to answer this exceptionally important question. But it was challenging as the reviewer might appreciate given the already very complex experimental design we have. And the short answer to the reviewer's question is we don't really know. There are multiple animal studies to suggest that these windows of consolidation continue long after learning, into sleep, and even during the time of retrieval. Consequently, the current data cannot determine whether these dynamics persist for tens of minutes, several hours, or throughout the time window typically associated with fear-memory consolidation.

We believe this is an important direction for future research. Addressing this question will require longitudinal sampling of resting-state brain activity across multiple post-conditioning time points, ideally using repeated resting-state fMRI runs spanning the consolidation period. Such a design would allow characterization of the full temporal profile of conditioning-induced brain-state dynamics and determine whether their evolution parallels the time course of fear-memory consolidation reported in previous studies.

Beyond determining how long post-learning brain dynamics persist, we believe the reviewer's question **point an important future direction** for CAP-based studies of memory consolidation. Traditional consolidation research has primarily focused on determining how long post-learning neural changes persist. By contrast, transient-state frameworks may enable additional dimensions of quantification, including "**how rapidly** consolidation-related brain states emerge", "**how stable** their engagement becomes over time", and "**how their temporal evolution relates** to subsequent memory expression". Future longitudinal studies will therefore be important for determining whether these state-level metrics provide conclusions that are consistent with, or complementary to, traditional measures of memory consolidation.

To clarify this point, we add a new section into revised main text, discussion section as follows:

> "This observation naturally raises an important unresolved question: how long these state-level dynamics persist following fear learning. Prior work has suggested that fear-memory consolidation can continue for several hours following acquisition, with most studies focusing primarily on the duration of post-learning neural processes. Although the present study cannot directly address this question because only a single post-conditioning resting-state fMRI run was acquired, the CAP framework provides a natural extension of these traditional approaches. By explicitly modeling the temporal evolution of recurring brain states, CAP has the potential to characterize not only how long consolidation-related dynamics persist, but also how rapidly they emerge and how stable their engagement becomes over time. Addressing these questions will require repeated resting-state fMRI acquisitions spanning the consolidation period, allowing future longitudinal studies to characterize the full trajectory of conditioning-related brain-state dynamics. (Main text, p16, line,381)"

Comment 7. *The motor cortex activation across fear conditioning phases is very interestingly, do authors believe is merely motor preparation? could you please comment on this?*

Response: Yes, we believe it is so- a preparation for action sort of an activation, though it could also reflect something more about functions of the motor cortex that we still don't understand (much of how we thought the thalamus was a relay structure, or that the visual cortex is only for simple processing of visual cues). But we explored this more, insight of the reviewer's comment. In response to the reviewer's suggestion, we revisited this interpretation and now discuss these findings within a broader framework of threat-related sensorimotor engagement rather than attributing them solely to motor preparation. Recent studies suggest that motor cortical recruitment during fear processing is not limited to movement execution or response preparation, but is also associated with action readiness, defensive preparation, and threat anticipation. Accordingly, we now interpret the observed precentral cortex involvement as reflecting engagement of threat-related sensorimotor circuitry that may support motor preparation together with broader processes of action readiness, defensive preparation, and threat anticipation during fear learning.

Accordingly, we have revised the relevant discussion throughout the manuscript to adopt a more balanced interpretation of the motor cortical findings as follows:

> "…The hippocampal and amygdala components are central to associative threat learning and contextual memory encoding, the posterior insula supports interoceptive and salience processing of aversive stimuli, and the precentral cortex likely reflects engagement of threat-related sensorimotor circuitry that was associated with motor preparation, action readiness, defensive preparation, and threat anticipation during fear learning…. (Main text, p16, line,365)"

Minor points

Comment 8. *Figures are particularly hard to understand for non-experts. Could please the authors try to add more information? For example, Figure 1 in my opinion is not very clear, in particular it is not clear which moment of the experiment the numbered state refers to, this is understood only later in the text by referring to Figure 2;*
I suggest being consistent in the abstract (post conditioning or post-conditioning)

Response: We sincerely appreciate the reviewer's suggestion. We carefully considered revising the overall figure layout. However, because the current schematic is intended to preserve the sequential analytical workflow presented throughout the manuscript, substantial changes to the figure itself could inadvertently introduce additional complexity or inconsistency with the accompanying text. Instead, we chose to substantially expand the Figure 1 legend so that each analytical step in panel A is explicitly explained and can be understood independently of the main text. Specifically, we clarified (1) that the five numbered brain states represent coactivation patterns identified from the combined pre- and post-conditioning resting-state fMRI data, (2) that the state-transition diagram

illustrates dynamic switching among these recurrent states during resting-state fMRI and forms the basis for the engagement and transition analyses, and (3) that the connectivity schematic represents fear-learning–induced functional connectivity reorganization within the identified brain state. We hope these additions will improve the readability of Figure 1, and we add the related description in revised main text as follows:

> "Figure 1. Conceptual and Analytical framework for tracking fear-learning–induced dynamic neural mechanism. Participants completed a three-day experimental protocol (Milad fear learning paradigm) with fear conditioning and extinction. (A). On Day 1, participants underwent a fear conditioning paradigm, with resting-state fMRI acquired before (PRE) and after (POST) conditioning to capture learning-induced changes in spontaneous brain activity. Coactivation pattern (CAP) analysis was applied to the combined PRE- and POST-conditioning resting-state fMRI data. The five numbered brain states shown at the top of panel A represent recurrent coactivation patterns identified by the hidden Markov model from the combined resting-state scans. The schematic immediately below the conditioning paradigm illustrates representative transitions among these recurrent brain states during resting-state fMRI, and changes in state engagement and transition dynamics before versus after conditioning were used to quantify fear-learning–induced brain-state reorganization. At the bottom of panel A, functional connectivity was estimated within the identified fear-learning–related brain state to characterize conditioning-induced connectivity reorganization, with only significant connectivity changes illustrated schematically (Main text, p6, line,149)"

Comment 9. *Please provide more details on how the subjective judgments were asked, were they done with VAS?*

Response: We thank the reviewer for pointing out that the subjective rating procedure required further clarification. We have now expanded the Methods section to explicitly describe how subjective fear ratings were obtained. Specifically, after completion of the conditioning phase, participants completed a computerized questionnaire inside the MRI scanner. Using a computer mouse, they rated how afraid they were of each stimulus color both at the beginning and at the end of the conditioning session on a 9-point numerical rating scale (1 = least afraid, 9 = most afraid). These ratings were subsequently used to quantify subjective fear acquisition and extinction-related behavioral changes.

The corresponding description has now been added to the Methods section as follows:

> "Behavioral indices of fear learning and extinction were assessed using computerized subjective fear ratings collected inside the MRI scanner. After completion of the conditioning phase, participants completed a questionnaire in which they rated how afraid they were of each stimulus color at both the beginning and the end of the conditioning session. Ratings were provided using a 9-point

numerical rating scale (1 = least afraid, 9 = most afraid) by selecting the corresponding response with a computer mouse. The same procedure was repeated following the recall and renewal sessions to assess subjective fear memory expression. (Main text, p25, line,648)”

Responses to Reviewer #2

Comment (summary): *The present study addresses a genuinely important question, and the experimental design is a notable strength of the manuscript. The three-day multi-modal protocol, combining pre- and post-conditioning resting-state fMRI, individually targeted TMS during extinction, and multi-day memory recall and renewal testing, is carefully constructed and represents a methodologically ambitious human fear-learning paradigms in the recent literature. The integration of dynamic brain-state modeling with a predictive multivariate framework, and the use of the same participants across conditioning, neuromodulation, and memory expression phases, is commendable and provides a principled basis for the brain-brain predictive analyses, as well as the use of nested cross-validation scheme with permutation testing and rCCA with L2 penalization.*
However, numerous concerns spanning the abstract, introduction, methods, results, and discussion require clarification before the manuscript can be accepted.

Response: We sincerely thank the reviewer for the thoughtful and constructive overall assessment of our work. We greatly appreciate the reviewer's recognition of the strengths of our experimental design, multimodal longitudinal protocol, and predictive analytical framework. We are also grateful for the detailed comments identifying areas where the presentation, methodological description, and interpretation of our findings could be further strengthened.

In response to the reviewer's suggestions, we have carefully revised the manuscript throughout. Please see our point-to-point response to your comments below.

Abstract

***Comment 1**. Conflation of Brain Reorganization and TMS Effects (lines 25–28)*
The abstract states that the study examined "how fear learning dynamically reorganizes brain activity immediately after learning, and whether such reorganization can be modulated with TMS application during extinction learning to prospectively predict extinction-memory expression." This phrasing conflates two separate processes: fear-learning-induced brain reorganization (Day 1, before TMS) and TMS applied during extinction (Day 2). The reorganization indexed by the predictive model is a Day 1 phenomenon; TMS did not modulate it. The abstract should clearly distinguish between these two processes to avoid the impression that TMS caused the brain reorganization that predicted memory.

Response: We thank the reviewer for this important clarification. The brain reorganization used in the predictive analyses was quantified from Day 1 resting-state data prior to any TMS intervention. TMS was administered during extinction learning on Day 2. We have therefore revised this state on the updated manuscript as following:

> "Herein, we examined how fear learning dynamically reorganizes brain activity immediately after learning, and whether such reorganization serves as a predictive biomarker of subsequent extinction-memory expression under TMS-modulated extinction. (Main text, p2, line,28)"

***Comment 2**. Null Result Stated as a Finding (lines 40–41)*
The statement "but not under natural extinction" is presented as a substantive finding. As detailed further below, this cannot be presented as a true mechanistic dissociation. It should not be stated in the abstract without qualification.

Response: We agree that the original wording could be interpreted as presenting the absence of a significant association in the natural extinction condition as evidence for a mechanistic dissociation.

To address this concern, we have revised the Abstract and corresponding sections throughout the manuscript to avoid overinterpretation of the findings. The revised Abstract now reads as follows:

> "Critically, conditioning-induced functional connectivity reorganization within this state predicted individual differences in extinction recall- and renewal-related brain activation under TMS-modulated extinction (cross-validated; recall $r = 0.47$, $p = .001$; renewal $r = 0.37$, $p = .01$; permutation-tested), but no significant predictive associations were detected under natural extinction. In separate brain–behavior correlation analyses, the neural variates identified in the TMS-modulated extinction were associated with individual differences in behavioral expression. (Main text, p2, line,42)"

We also agree that a statistically significant effect in one condition and a non-significant effect in another condition do not, by themselves, establish a statistically significant difference between conditions. Therefore, we performed an additional direct statistical comparison of predictive performance between the TMS-modulated and natural extinction conditions. Specifically, we compared the cross-validated prediction correlations ($\Delta r = r(TMS) - r(NoTMS)$ using a paired permutation framework based on the same 1,000 permutation distributions used throughout the predictive analyses. This analysis demonstrated that prediction performance was significantly greater under the TMS-modulated extinction condition than under natural extinction for both extinction-memory recall (Δr, permutation $p = .002$) and renewal (Δr, permutation $p = .04$). The result provide direct statistical support for the related statements throughout the manuscript that

prediction performance was significantly stronger under TMS-modulated extinction than under natural extinction.

Comment 3. *Outcome Variables*
The abstract mentions both brain-to-brain and brain-to-behavior predictive associations in close proximity, which may create some ambiguity for readers regarding which correlations (r = 0.47, r = 0.37) refer to which outcome. A brief clarification distinguishing the two analyses more explicitly would improve readability.

Response: We thank the reviewer for this helpful suggestion. Our intention was for the reported cross-validated correlations (recall: r = 0.47; renewal: r = 0.37) to refer specifically to the brain-to-brain prediction analyses, whereas the subsequent behavioral analyses were intended to evaluate the behavioral relevance of the identified neural signatures. To improve clarity, we have revised the Abstract to explicitly distinguish these two analytical frameworks.

The original text has been revised to:

> "Critically, conditioning-induced functional connectivity reorganization within this state predicted individual differences in extinction recall- and renewal-related brain activation under TMS-modulated extinction (cross-validated; recall r = 0.47, p = 0.001; renewal r = 0.37, p = 0.01; permutation-tested), but no significant associations were detected under natural extinction. In separate brain–behavior correlation analyses, the neural variates identified in the TMS-modulated extinction were associated with individual differences in behavioral expression (Main text, p2, line,42)."

Introduction

Comment 4*. Methods Material in the Introduction*
A substantial amount of methodological detail appears in the introduction, I understand this may be needed due to the journal structure but including descriptions of the analytic pipeline, parcellation approach, and predictive modeling framework (lines 108–121) makes it too heavy. This material should either be relocated to the Methods section or be better explained what it means, why is it of specific relevance for the paper questions?

Response: We substantially revised this section to focus on the **scientific questions and conceptual rationale** of the study rather than **methodological detail**.

We revised this state on the updated manuscript as following:

“We hypothesized that fear learning would induce systematic reorganization of brain-state dynamics and that such learning-induced neural reorganization would contain information relevant to subsequent extinction-memory expression. To test this possibility, we quantified fear-learning–induced changes in spontaneous brain dynamics from pre- to post-conditioning and examined whether these changes predicted subsequent memory expression under TMS-modulated extinction and natural extinction. We further evaluated the behavioral relevance of these neural signatures by examining their relationship to individual differences in fear expression. These findings provide insight into the neural mechanisms of fear-learning–induced brain-state reorganization and demonstrate that such learning-induced brain-state reorganization may serve as a prospective neural biomarker linking fear acquisition to subsequent memory expression. An illustration of our conceptual and analytic framework is shown in Figure 1 (Main text, p5, line,134).”

Comment 5. *Typographical Errors (lines 88, 112)*
Line 88 contains the phrase "Withing this process," which should read "Within this process." Line 112 reads "TMS navigate extinction," which is grammatically incorrect and should be revised (e.g., " TMS promotes individualized memory expression extinction").

Response: We have revised these phrases on the revised manuscript as following:

“Within this process, transcranial magnetic stimulation …… (Main text, p4, line103)”

“…. subsequent memory expression under TMS-modulated extinction and natural extinction (Main text, p5, line128)”

Comment 6. *TMS Rationale Insufficiently Specified (lines 89–97)*
The introduction describes TMS as "a widely used neuromodulation approach for probing the causal role of prefrontal control circuitry in fear-memory updating" but does not specify the expected direction of effect, whether stimulation was intended to enhance or suppress prefrontal activity, and by what mechanism. The introduction should state clearly whether the expected effect of DLPFC stimulation was facilitatory or inhibitory, and what the a priori prediction was regarding its effect on extinction memory. If TMS application was exploratory in nature, this should be explicitly stated and findings should be framed accordingly throughout the manuscript.

Response: We thank the reviewer for this important comment. Our objective was to **facilitate vmPFC-related regulatory circuitry involved in extinction-memory expression**.

This rationale was motivated by converging evidence from both animal and human studies. In rodents, our original rodent studies (Milad & Quirk, 2002; Milad et al., 2004) demonstrated that the ventromedial prefrontal cortex (vmPFC) plays a critical role in extinction-memory expression, with vmPFC neurons selectively signaling the retrieval of extinction memory[4,5] and that connectivity-guided stimulation of dorsolateral prefrontal cortex (DLPFC) can enhance extinction memory in humans through secondary modulation of vmPFC circuitry. Building upon these findings, subsequent human studies showed that connectivity-guided stimulation of the dorsolateral prefrontal cortex (DLPFC) can enhance extinction memory through secondary modulation of vmPFC-centered regulatory circuitry[6] (Raij et al., 2018).

This facilitatory rationale was also reflected in the stimulation parameters used in the current study. Prior TMS studies have consistently shown that low-frequency stimulation is generally associated with suppressive effects on cortical excitability, whereas higher-frequency stimulation (typically 5–25 Hz) is more commonly associated with facilitatory effects on the stimulated network[7,8] (Chen et al., 1997; Pascual-Leone et al., 1994). To remain consistent with the mechanistic rationale of enhancing extinction-related regulatory circuitry, we employed high-frequency (20 Hz) stimulation with the expectation of facilitating activity within the targeted DLPFC-vmPFC network and thereby promoting extinction-memory expression (See more details in Supplement).

To clarify this point, we have revised the Introduction to explicitly state the expected facilitatory nature of the intervention and its hypothesized effect on extinction memory as following:

> "Within this process, we leveraged transcranial magnetic stimulation (TMS) as a mechanistically motivated neuromodulation approach targeting dorsolateral prefrontal cortex (DLPFC) — designed to indirectly modulate ventromedial prefrontal cortex (vmPFC) function through connectivity-guided targeting — to facilitate extinction memory, consistent with prior rodent and human studies. (Main text, p5, line107)"

Comment 7*. Transient Brain States vs. Resting-State Networks (lines 299–301)*
The authors state that "human neuroimaging studies have rarely characterized how fear learning reorganizes brain dynamics at the level of transient brain states." This claim requires substantial qualification. There is an extensive literature examining resting-state network reorganization following fear conditioning using both seed-based and data-driven approaches. For example, Schultz et al. (2012) demonstrated that amygdala resting-state functional connectivity is significantly altered following Pavlovian fear conditioning, with increased amygdala-prefrontal coupling post-conditioning that correlated with behavioral fear indices, a finding directly relevant to the present studys design of acquiring resting-state fMRI before and after conditioning. Zidda et al. (2018)

further showed that resting-state default mode network connectivity, specifically its coupling with the amygdala and hippocampus, is linked to individual differences in cue and context fear conditioning and predictive of trait anxiety, demonstrating that static resting-state network measures already carry meaningful individual-difference information about fear learning outcomes. Longitudinal work has further demonstrated that these alterations are not transient. Martynova et al. (2020) showed that increased resting-state functional connectivity of the left amygdala following fear conditioning persisted across immediate post-learning, 24-hour, and one-week scanning sessions in healthy participants, directly paralleling the pre-post conditioning design used here. Tambini et al. (2017) showed that amygdala-hippocampal functional connectivity increased progressively during post-acquisition and post-extinction rest, and that this persistent post-learning connectivity was stronger in participants who showed spontaneous recovery of fear 24 hours later, linking resting-state connectivity changes to subsequent memory expression in a manner that conceptually overlaps with the present papers core claim. Feng et al. (2014; 2016) further demonstrated that amygdala-dACC and hippocampus-insula resting-state connectivity were enhanced during fear memory consolidation, while amygdala-mPFC coupling was decreased, and that the change in amygdala-mPFC functional connectivity predicted individual subjective fear ratings and that resting-state functional connectivity between the amygdala and vmPFC following fear memory reactivation predicted subsequent extinction efficacy in a multi-day paradigm, again using a multi-session design comparable in structure to the present study. Given this body of literature, the authors claim of novelty cannot rest on the mere use of resting-state fMRI in the context of fear learning. The authors must clearly articulate what their coactivation pattern and hidden Markov model approach offers that prior static functional connectivity work does not, specifically how characterizing transient, moment-to-moment brain state dynamics differs from and extends prior network-level analyses. The authors do cite Marstaller et al. (2016; their ref. 41), who applied dynamic network methods to fear conditioning, which further underlines the need to more precisely situate the present contribution within the existing dynamic neuroimaging literature. Without this clarification, the framing of the papers novelty is overstated.

Response:

We thank the reviewer for this thoughtful and constructive comment.

In response to this comment, we substantially revised both the Introduction and Discussion to more accurately position our work within the existing literature. Specifically, we now explicitly summarize prior evidence showing that fear learning induces persistent reorganization of resting-state functional connectivity and that these alterations are associated with subsequent behavioral and memory-related outcomes. We further

acknowledge that dynamic neuroimaging approaches have increasingly been applied to affective and learning-related processes.

We state in our revised manuscript that **our contribution is to characterize fear-learning–induced reorganization at the level of transient brain-state architecture and to determine whether such state-level reorganization prospectively predicts subsequent extinction-memory expression under neuromodulation.** In contrast to conventional resting-state connectivity analyses that primarily quantify average connectivity strength between regions or networks, our CAP-based framework allows characterization of the engagement, persistence, and switching behavior of recurring large-scale brain states.

We have revised the Introduction accordingly. Specifically, we added a new section summarizing prior resting-state connectivity findings following fear conditioning and introduced a clearer distinction between static connectivity approaches and transient brain-state frameworks. We also revised the Discussion to emphasize that our findings extend, rather than replace, previous resting-state connectivity studies by providing a complementary perspective on how fear learning reorganizes the temporal architecture of spontaneous brain activity.

The related revised text is as follows:

> "Understanding the underlying neural mechanisms of how conditioned threat memories are generated, consolidated, and updated is central to explaining the neurobiology of human defensive behavior, and to informing interventions for anxiety- and trauma-related disorders[4,9,10]. Extensive neuroimaging research has identified the large-scale neural circuitry supporting fear conditioning and extinction, including the amygdala, hippocampus, insula, anterior cingulate cortex, and ventromedial prefrontal cortex (vmPFC) [11–17]. Additionally, converging evidence from both animal and human studies suggests that fear learning is inherently dynamic. In rodent models, the engagement of key fear-learning structures, including the amygdala, hippocampus, and vmPFC, varies across different stages of acquisition, consolidation, and extinction[18–25]. Post-learning consolidation processes emerge immediately following learning and continue to evolve over time [26]. Consistent with these observations, human neuroimaging studies have demonstrated that fear conditioning induces measurable reorganization of spontaneous brain activity during post-learning. Using both seed-based and data-driven approaches, prior work has shown that fear conditioning alters resting-state functional connectivity within amygdala-prefrontal, amygdala-hippocampal, and default-mode network systems[27,28], that these changes can persist across consolidation periods ranging from hours to days, and that they are associated with subsequent fear expression, extinction efficacy, and individual differences in anxiety-related traits[29–32]. Collectively, these findings suggest that fear learning leaves a measurable and temporally evolving trace on large-scale

brain organization that extends beyond the learning episode itself and contributes to subsequent memory expression.

Despite these advances, prior work has largely characterized post-learning brain reorganization at the level of regional connectivity or network interactions averaged across entire scans. While such approaches provide important information regarding average patterns of functional organization, they offer limited insight into the moment-to-moment evolution of spontaneous brain activity. Comparatively less is known about whether fear learning reorganizes spontaneous activity at the level of recurring transient brain states, including how frequently such states occur, how long they persist, and how they transition over time. Consistent with a broader shift in systems neuroscience, recent frameworks are increasingly moving beyond static descriptions toward dynamic models that characterize cognition and learning in terms of transitions -through time during and after learning- among a set of recurrent brain states[33–36]. For example, Sendi et al demonstrated that alterations in the engagement and stability of specific brain states are associated with subsequent symptom expression following trauma exposure, highlighting the clinical relevance of dynamic state organization in the early aftermath of learning-related stress[37]. Longitudinal work from Awada et al further showed that reproducible coactivation patterns constitute stable brain-state signatures linked to motivational and affective disturbances in psychiatric populations[38]. Complementarily, clinical trial research from Cai et al revealed that treatment response in major depressive disorder and bipolar disorder is reflected in reorganization of brain-state engagement and transition probabilities[39]. These findings illustrate that characterizing state-to-state switching dynamics provides a principled framework for interrogating how cognitive processes and neuromodulation reshape functional brain organization over time.

However, the specific contribution of transient brain-state organization to fear-learning consolidation and subsequent memory expression remains insufficiently understood. In particular, it remains unknown whether fear-learning–induced reorganization of spontaneous brain-state architecture can prospectively predict subsequent extinction-memory expression and neuromodulation efficacy. (Main text, p4, line 96)”

We also state the advantage of HMM and revised the Discussion as follows:

“Previous studies have established that fear learning induces persistent reorganization of resting-state functional connectivity, with such changes being associated with subsequent fear expression and extinction outcomes. Extending this literature, our state-based framework characterizes how fear learning reshapes the temporal architecture of spontaneous brain activity, including the engagement, persistence, and switching of recurring large-scale brain states. Within this framework, the Hidden Markov Model offers a critical advantage by jointly modeling both spatial topologies and temporal transition probabilities at a single-TR resolution. Unlike conventional sliding-window approaches that suffer

> from arbitrary temporal smoothing, or standard frame-wise clustering that discards temporal order, this generative probabilistic approach bypasses window-size dependencies, thereby enabling the tracking of instantaneous, non-linear network switching driven by fear learning. Leveraging this approach, we demonstrated that fear learning selectively amplifies a distributed threat-related brain state shortly after learning. This state was characterized by coordinated engagement of medial temporal, insular, cingulate, and sensorimotor regions, consistent with an integrated network supporting threat encoding, interoceptive salience, and defensive action preparation (Main text, p15, line 358)"

Comment 8. *Overclaiming in the Closing Statement (lines 118–121)*
The final sentence of the introduction states that the study "demonstrates that these learning-induced dynamics can be formalized as neural biomarkers that effectively predict extinction memory expression after neuromodulation." Given the absence of external validation, the inverted feature-to-sample ratio, and the modest cross-validated effect sizes, the term "effectively" overstates the strength of the evidence. This should be tempered accordingly, for example by using "may serve as" rather than "can be formalized as."

Response: We thank the reviewer for this thoughtful comment and agree.

In response, we revised the state of the Introduction to adopt more cautious language. Specifically,

> "These findings provide insight into the neural mechanisms of fear-learning–induced brain-state reorganization and demonstrate that such learning-induced brain-state reorganization may serve as a prospective neural biomarker linking fear acquisition to subsequent memory expression. (Main text, p5, line 133)"

Methods

Comment 9. *Discrepancy in Cross-Validation Design*
The main manuscript describes a 5-fold nested cross-validation procedure, whereas the Supplementary Methods specify a 10-fold outer loop repeated five times with a 5-fold inner loop for hyperparameter selection. This is a substantive discrepancy that must be resolved. The authors should confirm which procedure was actually implemented and ensure consistency across the entire manuscript.

Response: We thank the reviewer for carefully identifying this inconsistency in the description of the cross-validation procedure. The discrepancy arose from an error in reporting. We apologize for the inaccurate description.

To clarify, we carefully re-examined the original analysis code and raw outputs. We confirm that the predictive modeling analyses were implemented using a nested cross-validation framework with a 10-fold outer loop and a 10-fold inner loop for hyperparameter optimization. And the entire outer cross-validation procedure was repeated five times using different fold assignments.

We have now revised the main text, Supplementary Methods, and all related des description as following:

> "To prevent information leakage and circular inference, hyperparameter selection was performed exclusively within the training data using an inner 10-fold cross-validation procedure within a nested cross-validation framework (Main text, p. , line)"

> "In the outer loop, we conducted 10-fold cross-validation repeated five times. In each iteration, nine folds were used for model training and one fold for testing. Canonical weights derived from the training data were applied to the held-out test data to generate predicted canonical variate scores. Within each outer training set, an inner 10-fold cross-validation procedure was used to select the optimal regularization parameter. (Supplement, p16, line 195)"

***Comment** 10. Feature-to-Sample Ratio*

The predictive model ingests 276 functional connectivity features in a sample of N = 87, yielding a feature-to-sample ratio of approximately 3:1, substantially exceeding the commonly recommended minimum of 10 observations per predictor (Riley et al., 2019). While L2 regularization partially mitigates this imbalance, it does not eliminate the risk of unstable parameter estimation. The authors do not explicitly address how this inverted ratio may compromise the reliability of canonical loadings. A direct discussion of this issue, alongside evidence that the model is not capitalizing on noise, is warranted.

Response: We thank the reviewer for raising this important point.

We agree that the number of functional connectivity features exceeds the sample size, creating a high-dimensional modeling setting that can increase susceptibility to overfitting and instability if not properly controlled. For this reason, our analytical framework was specifically designed to minimize these risks.

First, we employed regularized canonical correlation analysis (rCCA), which incorporates L2 regularization to stabilize covariance estimation and reduce variance associated with high-dimensional feature spaces. Second, all predictive analyses were evaluated using a

nested cross-validation framework, ensuring that hyperparameter selection was performed exclusively within training data and that model performance was assessed on completely unseen participants. Third, statistical significance was determined using permutation testing, demonstrating that the observed predictive relationships exceeded chance-level expectations.

To directly address the reviewer's concern regarding the reliability of canonical loadings, we performed an additional non-parametric bootstrap stability analysis (1000 iterations). Across both recall and renewal models, the dominant connectivity and activation features identified in the original analyses consistently remained among the highest-loading contributors across bootstrap resamples, and their relative rankings were largely preserved (Details see **Response to Comment 20**). These findings suggest that the reported neural signatures were not driven by sampling-specific noise or a small subset of participants.

In response to the concern, we added stability analyses (Detailed result see Response to Comment 20) and related discussion in the revised manuscript. The related state is as follows:

> "One limitation is that the number of connectivity features exceeded the sample size, resulting in a high-dimensional prediction setting[40]. Although regularization, nested cross-validation, permutation testing, and bootstrap stability analyses were implemented to mitigate overfitting and assess model robustness, replication in larger independent cohorts will be important for establishing the generalizability of the identified neural signatures. (Main text, p19, line 459)"

In addition, we realized that the **original manuscript did not clearly report participant demographics and data attrition**, for which we apologize. We have now added a detailed description of participant inclusion and exclusion in the revised Methods. Briefly, 87 healthy participants (43 males and 44 females; age range 18–60 years, mean ± SD = 28.79 ± 9.20 years) completed the fear-conditioning experiment. Because the study required complete imaging data across multiple sessions spanning three consecutive days, four participants were missing the post-conditioning resting-state scan and three participants were missing the Day 3 memory-expression imaging data. These participants were excluded from subsequent analyses, resulting in a final sample of 80 participants (41 males and 39 females; mean age = 28.36 ± 8.33 years, range = 18–60 years) included in all predictive analyses. We have revised the manuscript accordingly to clearly report the final analytical sample used throughout the prediction analyses.

Comment 11*. Definition and Justification of Five Coactivation States (lines 150–158)*
The authors state that "Five distinct recurring patterns of spatial coactivation pattern was defined as basic brain state across all participants," a sentence that contains a

grammatical error ("patterns... was") that should be corrected. More substantively, the rationale for five states is not presented in the main text. The main text should briefly describe the model selection criteria and why their convergence at k=5 was deemed sufficient. All acronyms introduced in lines 153–158 (dAI, vmPFC, sgACC, MFG, SMA, PCC) are undefined at first use and must be spelled out. Finally, the state labels appear to be assigned post-hoc based on spatial profiles, and the criteria and thresholds used to assign these interpretive labels should be made explicit.

Response: We thank the reviewer for these helpful comments.

First, we corrected the grammatical error in the original sentence. The revised text now reads:

> "Five distinct recurring coactivation patterns were defined as basic brain states across all participants. (Main text, p7, line 171)"

Second, we agree that the rationale for selecting a five-state solution should be more explicitly described in the main text. Consistent with prior studies[37,41], the optimal number of states was determined using clustering-validity metrics that quantify the balance between within-cluster similarity and between-cluster separability. Following this literature, we quantified clustering quality using two independent distance-based validity indices: the Davies–Bouldin index and the Ray–Turi index[42]. Both metrics converged on a five-state solution (k = 5), indicating an optimal balance between within-state homogeneity and between-state separability. The related state was provided in "page 23, line567" on main text and the corresponding clustering-validity results in Supplementary Figure S2.

Third, all anatomical abbreviations are now defined at first use. All abbreviations for the ROIs are defined in the legend of Figure 1.

Finally, we agree that the criteria used to assign interpretive state labels should be made more explicit. Our state-labeling strategy was consistent with prior studies that assign descriptive labels according to dominant spatial activation profiles and the most prominently engaged regions or networks[43–45]. Specifically, we quantified the regional activation profile of each state, normalized the activation weights for visualization, and ranked all 24 predefined ROIs according to their activation weights. No predefined numerical threshold was used to assign state labels. Instead, descriptive labels were based on the dominant positive and negative regional contributions across the complete spatial activation profile, together with their correspondence to well-established functional neuroanatomical systems. For clarity of visualization, only the ROIs exhibiting the largest absolute normalized activation weights were displayed in the figure.

To improve transparency and interpretability, we have now added a new section in the Supplementary Materials detailing the rationale and criteria used for state labeling. The added text reads as follows:

"To improve interpretability, the five coactivation states were labeled according to their dominant spatial activation profiles. Specifically, state labels were assigned based on the ROIs showing the most prominent positive and negative activation weights, following a commonly adopted approach in CAP- based studies in which states are descriptively named according to their most salient regional or network-level activation patterns. The spatial activation profiles of all five states are shown in Figure S3. (Supplement, p11, line 299)"

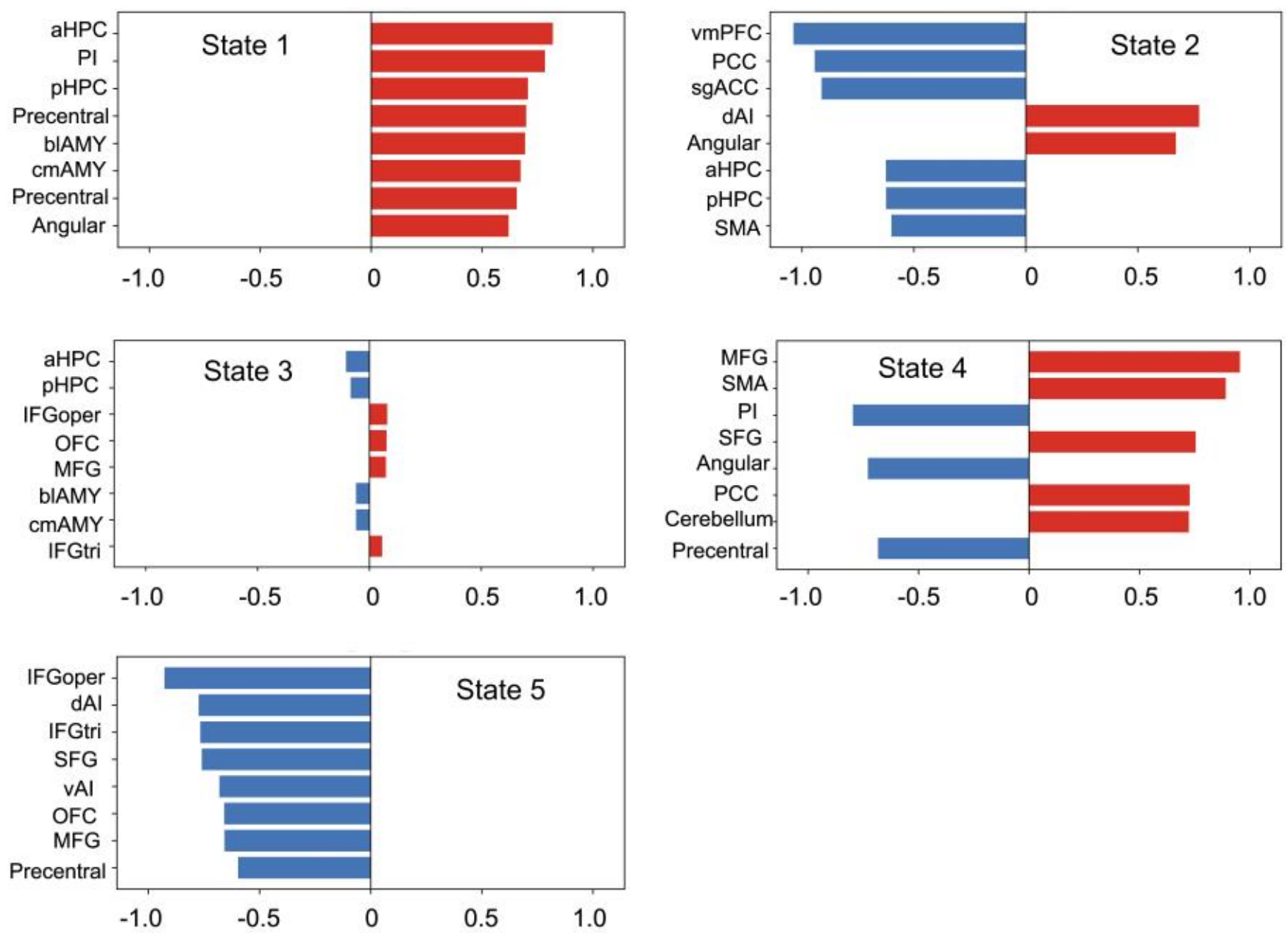

Figure S2. Spatial activation profiles of the five coactivation states. For each state, red bars indicate the ROIs with the strongest positive activation weights and blue bars indicate the ROIs with the strongest negative activation weights."

***Comment 12**. TMS Rationale, Expected Direction, and Hypotheses*
As noted in the introduction, it is not stated whether stimulation was intended to be inhibitory or excitatory, which circuit was expected to be modulated, or what the a priori hypotheses were. If this was an exploratory application of TMS, it should be framed explicitly as such throughout the methods, and all TMS-related findings should be interpreted as hypothesis-generating.

Response: To clarify this point, we have revised the Methods section as following:

"…Stimulation was delivered to a target within the left posterior dorsolateral prefrontal cortex (DLPFC), defined as the cortical region showing the strongest and most reliable functional connectivity with the ventromedial prefrontal cortex (vmPFC), a key node implicated in extinction memory. The stimulation protocol was designed as a facilitatory neuromodulation intervention with the a priori goal of enhancing extinction-memory expression. (Main text, p21, line 536)"

Comment 13. *Outlier Handling and Correlation Type*
The manuscript does not report whether outliers were assessed or excluded from the correlation analyses. Given the scatter visible in Figure S6, several data points appear to exert considerable leverage on the reported correlations. The authors should report: (a) whether outlier screening was performed and by what criterion; (b) whether results survive exclusion of potential outliers; and (c) whether Pearson or Spearman correlations were used and why, given that normality of canonical variate scores cannot be assumed.

Response: First, we would like to clarify the analysis sample used throughout the predictive modeling. Although 87 participants completed the experimental protocol, 4 participants were excluded because the post-conditioning resting-state fMRI required to derive the fear-learning brain reorganization features was unavailable, and an additional 3 participants were excluded because the Day 3 memory-expression fMRI data required for the outcome measures were unavailable. Consequently, all rCCA analyses—including the primary brain-to-brain prediction models (Figure 4), the brain-to-behavior analyses (Figure 6), and the supplementary analyses presented in Figure S6—were performed on the same final sample of 80 participants.

We would also like to clarify an important distinction between the primary predictive analyses reported in the main text and the supplementary brain–behavior analyses shown in Figure S6 (**Similar Concern** from **Comment 15**). The primary findings of this study are based on brain-to-brain prediction models linking fear-learning–induced functional connectivity reorganization to later memory-expression activation patterns. These analyses were evaluated using **nested cross-validation and permutation-based inference**. While the analyses presented in Figure S6 (now Figure S9) were exploratory **in-sample** brain–behavior association analyses. In the original submission, these supplementary analyses were reported using **in-sample rCCA correlations** and **did not include permutation-based significance testing**. Following the reviewer's comments, we have now incorporated permutation testing for all brain–behavior analyses and revised the corresponding results accordingly (More details see **Response to Comment 15**). As reported in the revised manuscript, none of the associations shown in Figure S6 survived permutation testing (all $p > .05$). Consequently, these analyses are now interpreted as exploratory control analyses rather than primary evidence supporting brain–behavior coupling. Importantly, because the revised conclusions no longer rely on statistical significance of these associations, the interpretation is substantially less sensitive to the influence of any individual observation.

In response to concern *(a) whether outlier screening was performed and by what criterion*. Within this final analysis cohort (N = 80), no participants were excluded from any

correlation or predictive analyses on the basis of outlier screening or influential observations.

Regarding the concern *(b) whether results survive exclusion of potential outliers and several data points appear to exert considerable leverage on the reported correlations,* we performed an additional sensitivity analysis using Cook's distance. Specifically, a simple linear regression was fitted between the two canonical variate scores, and Cook's distance was calculated for each participant. Following a commonly adopted criterion, observations with Cook's distance greater than 4/N were considered potentially influential. We then repeated the correlation analyses after excluding these observations. The resulting Pearson and Spearman correlations remained highly similar to the original analyses, with no change in the overall statistical conclusions. These findings indicate that the reported associations are not driven by a small number of influential observations.

Regarding the concern (c) *whether Pearson or Spearman correlations were used and why*, we would argue that Pearson correlations were originally used because canonical loadings and canonical variate relationships in CCA/rCCA are conventionally defined using Pearson correlation. Nevertheless, as a robustness check, we additionally evaluated Spearman rank correlations and observed a similar overall pattern of associations, indicating that the observed relationships were not solely driven by assumptions of linearity or normality.

We have clarified these points in the revised manuscript and Supplementary Materials as follows:

> "To examine whether the stronger brain–brain predictive relationships observed under TMS-modulated extinction could be attributed to intrinsic differences between the two conditioned stimuli, we separately evaluated associations between fear-learning–induced brain reorganization and behavioral outcomes for each conditioned cue across recall (CS+TMS, CS+NoTMS) and renewal (CS+TMS, CS+NoTMS) conditions (Figure S9). Because the CS+TMS and CS+NoTMS stimuli corresponded to distinct conditioned cues acquired during Day 1 fear learning (CS+1 and CS+2), this analysis allowed us to assess whether cue identity influenced subsequent brain–behavior coupling.
>
> Although moderate-to-large correlation coefficients were observed across all four conditions (recall CS+TMS: $r(78) = 0.70$; renewal CS+TMS: $r(78)=0.71$; recall CS+NoTMS: $r(78) = 0.72$; renewal CS+NoTMS: $r(78) = 0.73$), none of these associations survived permutation-based significance testing (recall CS+TMS: $p = .93$; renewal CS+TMS: $p = .87$; recall CS+NoTMS: $p = .60$; renewal CS+NoTMS: $p = .59$). The magnitude of the observed associations was highly similar across the two conditioned cues and across recall and renewal phases.
>
> To evaluate whether the reported correlations were disproportionately influenced by a small number of observations, we performed an additional sensitivity analysis

using Cook's distance. A simple linear regression was fitted between the two canonical variate scores, and Cook's distance was computed for each participant using the fitted regression model. Following a commonly used criterion, observations with Cook's distance greater than 4/N were considered potentially influential. Pearson and Spearman correlation analyses were then repeated after excluding these observations to assess the robustness of the reported associations. Repeating the correlation analyses after excluding these observations yielded Pearson correlation coefficients that were highly similar to those obtained using the complete dataset, and all major statistical conclusions remained unchanged. (recall CS+TMS: r(78) = 0.67; renewal CS+TMS: r(78)=0.65; recall CS+NoTMS: r(78) = 0.75; renewal CS+NoTMS: r(78) = 0.75; all p>.05)

As another robustness analysis, Spearman rank correlations yielded a similar pattern of associations across all four conditions (recall CS+TMS: r(78) = 0.41; renewal CS+TMS: r(78)=0.71; recall CS+NoTMS: r(78) = 0.53; renewal CS+NoTMS: r(78) = 0.67, all p>.05), indicating that the observed relationships were not driven by assumptions of normality or linearity.

Above all, the enhanced brain–brain predictive relationships observed in the TMS-modulated extinction condition are unlikely to be explained by cue-specific differences established during fear acquisition. (Supplement, 20, line 490)"

***Comment 14**. Grammatical Error in Methods Narrative (lines 188–190)*
The phrase "brain circuits to related to fear learning" (lines 188–189) contains a typographical error. More broadly, the sentence is syntactically unclear and should be rewritten for precision.

Response: We thank the reviewer for identifying this issue. To improve clarity and precision, we rewrote the sentence on the revised manuscript:

"If fear learning induces a meaningful reorganization of large-scale brain-state dynamics, this reorganization would be expected to continue evolving during the post-learning resting period rather than emerge as an instantaneous shift. If so, a temporally-graded shift in these states would be expected (Main text, p9, line 216)"

Results

***Comment 15**. Inconsistency Between Out-of-Sample and Supplementary Correlations*
The main results report out-of-sample canonical correlations of r = 0.47 (recall, p = 0.001) and r = 0.37 (renewal, p = 0.01) for the TMS condition. However, Figure S6 in the supplement reports brain-behavior correlations of r = 0.70–0.73 across all four conditions, including CS+NoTMS. This magnitude gap is substantial and unexplained. The authors must clarify whether the Figure S6 values are cross-validated or in-sample

estimates. In-sample correlations of r > 0.70 in N = 87 with 276 input features would constitute a serious overfitting concern and would directly undermine the cross-validation framework the paper is built upon. This is arguably the most critical inconsistency in the manuscript and must be addressed directly.

Response: We thank the reviewer for highlighting this important issue. We agree that the original manuscript **did not sufficiently distinguish** between the **cross-validated brain-to-brain prediction** analyses reported in the main text and the **in-sample brain-to-behavior association** analyses presented in Figure 6 and Supplementary Figure S6.

To clarify the analytical differences among these analyses, we have added the following summary table to clarify the **previous** strategies we applied：

Table. Analytical Details for Predictive and Correlation Analysis **on Raw Version**

Analysis	Figure	Input Size (X)	Input Size (Y)	Strategy	Statistical Inference
Brain to Brain	Figure 4	Subj x 276	Subj x 24	Nested Cross validation	Permutation test
Brain to Behavior	Figure 6	Subj x 24	Subj x 1	In Sample	No Permutation test
Brain to Behavior	Figure S6	Subj x 276	Subj x 1	In Sample	No Permutation test

Importantly, the **nested cross-validation framework and permutation-based inference** described in the main manuscript were applied exclusively to the **brain-to-brain** predictive analyses. These analyses were designed to evaluate out-of-sample prediction performance between two multivariate neural datasets and therefore constitute the primary predictive framework of the study.

By contrast, the **brain-to-behavior analyses** were conducted as **in-sample association** analyses. These analyses were intended to examine whether neural variates identified from the brain models were associated with individual differences in behavioral expression. **We did not perform nested cross-validation**, which precluded a formal hyperparameter optimization step. So, we **fixed** the regularization parameter at c = 1. Consequently, these analyses should not be interpreted as predictive output or compared directly with the cross-validated correlations reported for the brain-to-brain analyses.

We agree with the reviewer that the statistical nature of these analyses was insufficiently described in the original manuscript. **In the revised version**, we have added **Permutation testing** to all **brain-to-behavior** association analyses and updated the corresponding results accordingly.

Regarding the relatively high correlations observed in Supplementary Figure S6, we agree that in-sample rCCA can produce inflated correlation estimates when model fitting and evaluation are performed on the same dataset, particularly when the predictor space is high-dimensional (Subjects × 276) relative to sample size. To address this concern, we now explicitly describe these analyses as in-sample associations. Additionally, we

supplemented the results for Figure S6 with a permutation test and found that, **despite relatively high correlation, none of the groups passed the permutation test**.

We have revised the Results, Supplementary Materials, figure legends, and methodological descriptions throughout the manuscript to clearly distinguish predictive (cross-validated, out-of-sample) analyses from associative (in-sample) analyses and to prevent direct comparison between these fundamentally different statistical quantities.

Building upon the analysis of Figure 6, we added a permutation test and revised the descriptions of the figures and results in main text, as detailed below:

> "To further examine the relationship between neural dynamics and behavioral expression, we conducted multivariate correlation analyses linking individual differences in behavior to brain-derived measures. As shown in Figure 6A and 6B, TMS modulated extinction recall memory (r=0.33, p=.01) were significantly associated with subjective fear ratings. In contrast, no significant correlation was observed between natural extinction (CS+NoTMS) memory and behavioral scores (recall: r=0.14, p=.95; renewal: r=0.19, p=.85). Although the renewal analysis did not reach statistical significance following permutation testing, the effect size remained larger in the TMS condition than in the natural extinction condition. These findings suggest that significant brain–behavior associations were observed only for TMS-modulated extinction recall memory, while the overall pattern remained consistent with stronger brain–behavior coupling under TMS-modulated compared with natural extinction learning. (Main text, p14, line 319)

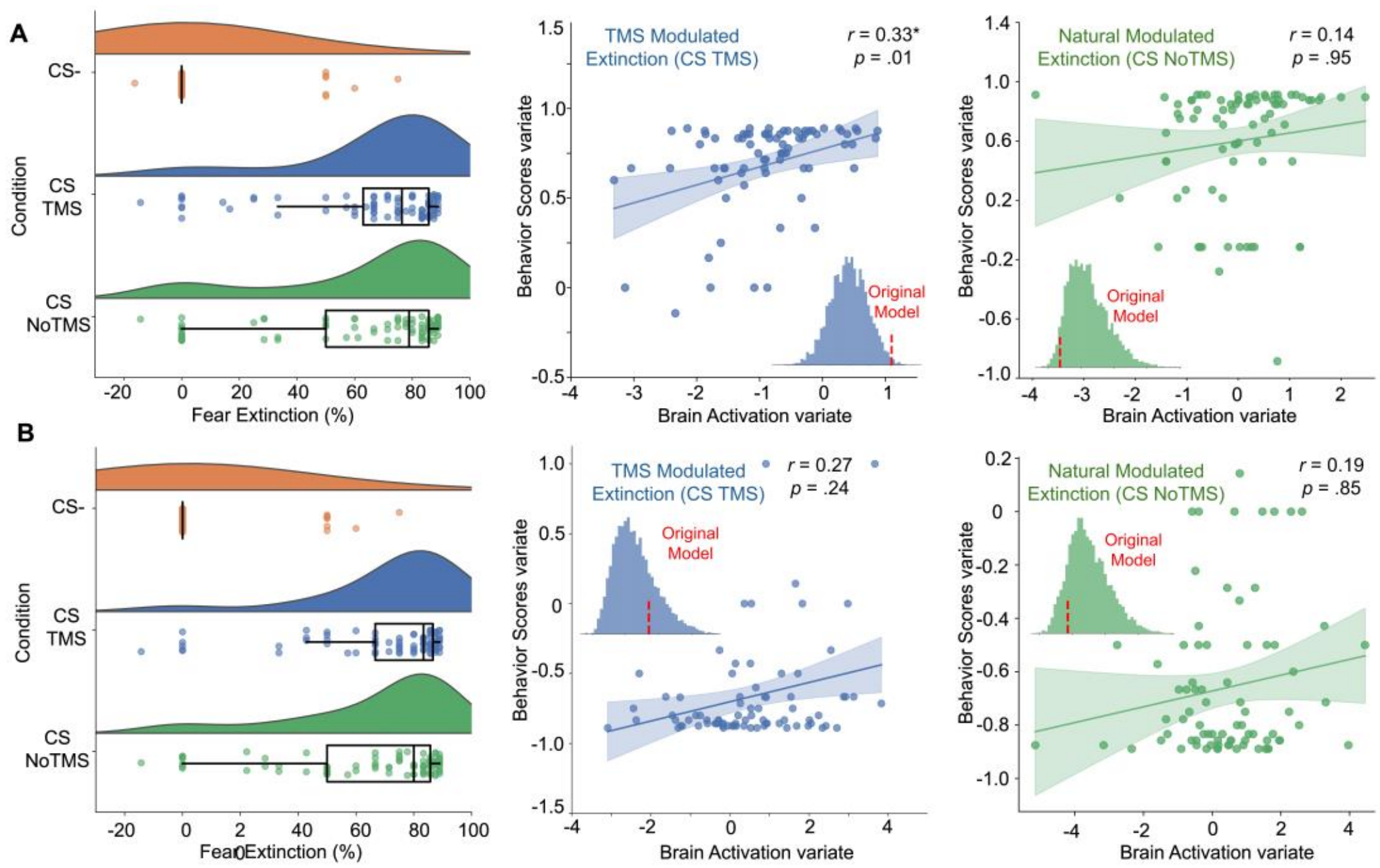

Figure 6. Behavioral expression of extinction memory and its association with neural measures. (A) Subjective fear ratings during the memory recall phase. (B) Subjective fear ratings during the memory renewal phase.

At the same time, we have revised the description of the discussion regarding brain reorganization-behavior correlations in the main text as follows:

"Subjective fear ratings indicated that extinction was evident in both stimulation conditions: fear responses decreased from conditioning to the post-extinction memory phase for both CS+TMS and CS+NoTMS, whereas CS− showed minimal change relative to baseline. Although the magnitude of behavioral extinction did not significantly differ between CS+TMS and CS+NoTMS, extinction tended to be stronger in the TMS condition. To further examine whether the observed TMS-related effects could be attributed to pre-existing differences between the two conditioned cues, we explored the relationship between fear-learning–induced brain reorganization and subsequent behavioral change separately for the CS+TMS and CS+NoTMS conditions (i.e., two conditioned threat cues CSp1 and CSp2 established during Day 1). Following permutation-based inference, none of these brain–behavior associations reached statistical significance (Figure S9). Importantly, the overall effect-size patterns were comparable across the two CS+ conditions, providing no evidence that one cue exhibited systematically stronger coupling between fear-learning–induced reorganization and behavioral outcome prior to extinction learning. These findings suggest that the stronger brain–brain predictive relationships observed under TMS-modulated extinction are unlikely to be explained by intrinsic differences between the conditioned cues established during fear acquisition. (Main text, p18, line 35)"

Additionally, we have also revised the description of the relevant results in the supplementary materials:

"To examine whether the stronger brain–brain predictive relationships observed under TMS-modulated extinction could be attributed to intrinsic differences between the two conditioned stimuli, we separately evaluated associations between fear-learning–induced brain reorganization and behavioral outcomes for each conditioned cue across recall (CS+TMS, CS+NoTMS) and renewal (CS+TMS, CS+NoTMS) conditions (Figure S9). Because the CS+TMS and CS+NoTMS stimuli corresponded to distinct conditioned cues acquired during Day 1 fear learning (CS+1 and CS+2), this analysis allowed us to assess whether cue identity influenced subsequent brain–behavior coupling.

Although moderate-to-large correlation coefficients were observed across all four conditions (recall CS+TMS: $r = 0.70$; renewal CS+TMS: $r = 0.71$; recall CS+NoTMS: $r = 0.72$; renewal CS+NoTMS: $r = 0.73$), none of these associations survived permutation-based significance testing (recall CS+TMS: $p = .93$; renewal CS+TMS: $p = .87$; recall CS+NoTMS: $p = .60$; renewal CS+NoTMS: $p = .59$). This apparent discrepancy is expected because rCCA explicitly identifies linear projections that maximize the correlation between the two variable sets; therefore, in-sample

canonical correlations can be relatively high even when the association does not generalize beyond the observed sample. For this reason, we relied on permutation-based inference rather than the nominal correlation magnitude alone to determine statistical significance. The magnitude of the observed associations was comparable across the two conditioned cues and across recall and renewal phases (Supplement p19, line 470).

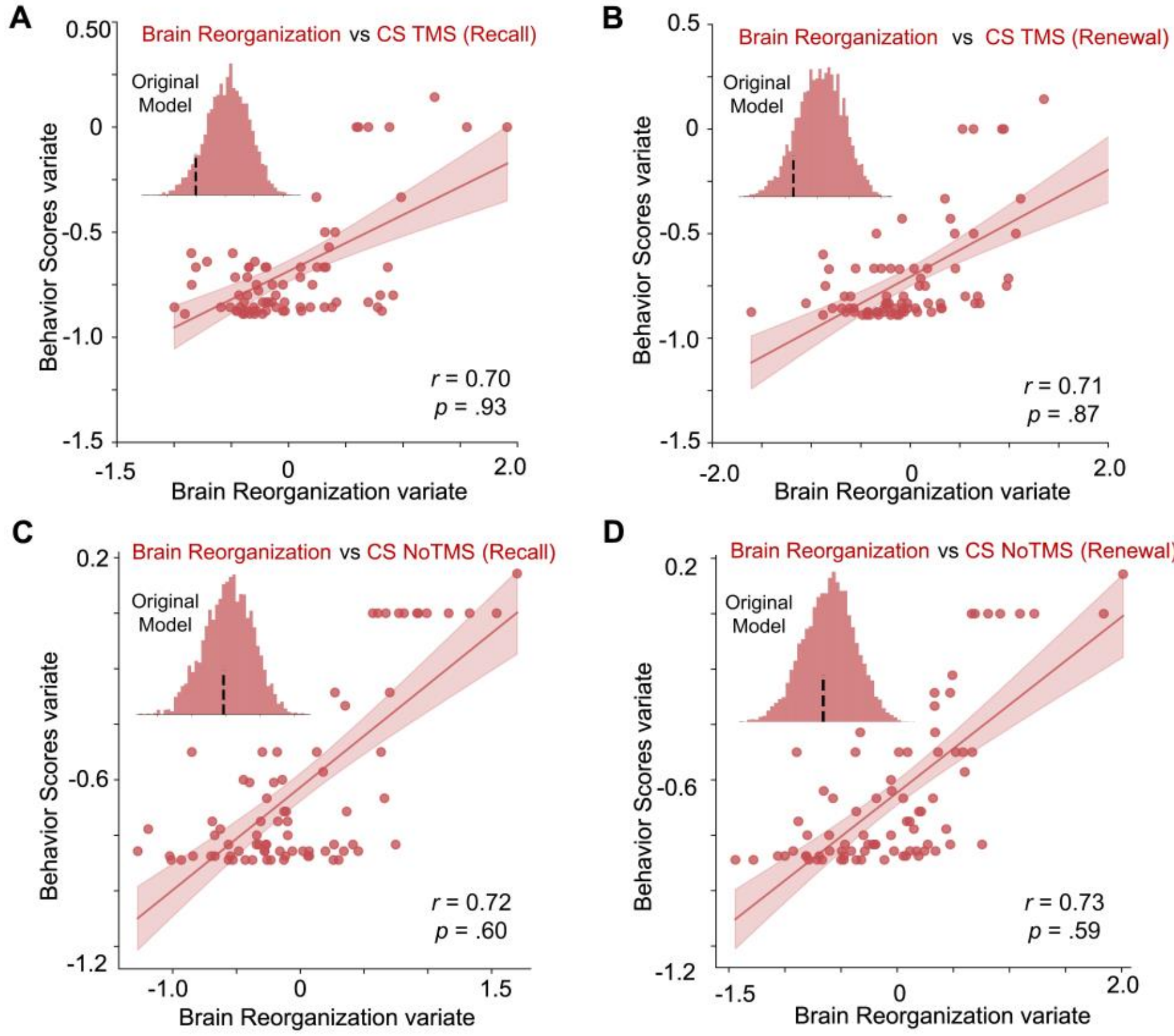

Figure S9. Correlations between fear-learning–induced brain reorganization and behavioral scores across four experimental conditions. (A) Recall under CS+TMS; (B) Renewal under CS+TMS; (C) Recall under CS+NoTMS; (D) Renewal under CS+NoTMS.
"

***Comment 16**. Result Reporting Does Not Follow Conventions (lines 269–270)*
The sentence "TMS modulated extinction memory (recall: r=0.33, p=3e-3; renewal: r=0.27, p=0.01) were significantly associated with subjective fear ratings" does not follow standard reporting conventions and is grammatically incorrect. Results should be reported in standard format, e.g., r(85) = 0.33, p = .003. The subject-verb agreement error ("memory... were") should also be corrected.

Response: In the revised manuscript, we now report correlation results using the conventional format (e.g., *r*(df) = value, *p* = value) throughout the manuscript. We also corrected the grammatical error by revising the sentence structure.

Furthermore, because all brain–behavior analyses were re-evaluated using permutation-based inference during revision, the corresponding Results section has been updated accordingly.

Comment 17*. Undefined Modulation Direction (lines 272–274)*
The statement that "robust brain-behavior coupling emerged only when extinction was modulated by TMS" is insufficiently specific. The authors should state precisely in what direction and by what mechanism TMS modulated extinction-related brain activity and behavior, rather than using the uninformative term "modulated."

Response: We agree that the intended direction and mechanistic rationale of the TMS intervention should be stated more explicitly. In response, we have revised both the Introduction and Methods sections to clarify the theoretical basis of the stimulation protocol, the targeted DLPFC–vmPFC regulatory circuit, and our a priori expectation that connectivity-guided DLPFC stimulation was designed as a facilitatory neuromodulation intervention intended to enhance extinction-memory expression.

At the same time, we believe it is important to distinguish between the **hypothesized rationale motivating the intervention** and the **empirical findings directly** demonstrated by the present study. Although the stimulation protocol was designed with a facilitatory objective, the current study was not specifically designed to comprehensively characterize the neural mechanisms through which TMS influences extinction-memory expression. In particular, we lack comprehensive and direct evidence for this statement.

Therefore, we have chosen to adopt more conservative wording when describing the results. Rather than attributing the observed brain–behavior associations to a demonstrated facilitatory effect of TMS, we describe the findings in terms of the experimental condition under which they were observed. Specifically, the revised text states:

> “These findings suggest that significant brain–behavior associations were observed only for TMS-modulated extinction recall memory, while the overall pattern remained consistent with stronger brain–behavior coupling under TMS-modulated compared with natural extinction learning. (Main text p14, line319)”

We believe this wording more accurately reflects the empirical evidence while avoiding mechanistic interpretations that were not directly tested. We have additionally expanded the Discussion to acknowledge that a more definitive characterization of TMS-related mechanisms will require dedicated future studies incorporating stimulation-field modeling (e.g., E-field estimation), active control stimulation sites, on-target versus off-target comparisons, and multimodal neuroimaging approaches, including whole-brain functional MRI and structural connectivity analyses.

The related state was added into discussion section as following:

> "Third, although our stimulation protocol was designed as a facilitatory neuromodulation intervention targeting the DLPFC-vmPFC regulatory circuit, the present study was not designed to directly characterize the neural mechanisms through which TMS influences extinction-memory expression. In addition, our experimental protocol did not include sham stimulation or active stimulation over a control region. Therefore, although the observed predictive relationship was significantly stronger under the TMS-modulated condition within the present design, we cannot determine whether this finding reflects modulation of the targeted DLPFC-vmPFC circuit or nonspecific effects of active stimulation. Accordingly, the present findings should not be interpreted as direct evidence that TMS enhanced vmPFC activity or facilitated extinction-memory processes. A more mechanistic understanding of this effect will require future studies incorporating sham stimulation, active control stimulation sites, individualized electric-field modeling, on-target versus off-target stimulation comparisons, and multimodal neuroimaging approaches capable of directly characterizing stimulation-induced changes in large-scale brain networks. (Main text p19, line35)"

Discussion

Comment 18. *Definition of "Fear-Related Brain State" (lines 283–286)*
The discussion refers to "a fear-related brain state" as a central finding, but this label is not adequately justified. State 1 encompasses regions with broad functional roles including hippocampus, insula, and sensorimotor cortex. The authors should provide explicit justification for this label, for example by demonstrating that this states occurrence rate or dwell time correlates with conditioning-related indices, or that it is selectively engaged by CS+ versus CS- trials.

Response：We agree that the term "fear-related brain state" required clearer justification, and that more precise state labeling is important for improving the rigor and interpretability of the manuscript.

In response to the reviewer's suggestion, we first tested whether individual differences in the dynamic changes of State 1 were associated with explicit fear acquisition. Specifically, we computed the Explicit Acquisition Index as Fear rating (CS+) − Fear rating (CS−), and examined its association with ΔOCR (State 1) and ΔDT (State 1), defined as post-conditioning minus pre-conditioning occurrence rate and dwell time, respectively. These exploratory correlations were not statistically significant.

Instead, we polished the state-labeling strategy to make it more rigorous and accurate. Our state-labeling strategy was consistent with prior studies that label states according to

their dominant spatial activation profiles and the most prominently engaged regions or networks[43–45], Specifically, we quantified the regional activation profile of each state, ranked ROIs according to their activation weights, and assigned descriptive labels based on the most prominent positive and negative regional contributions. The resulting labels therefore reflect the dominant spatial organization of each state. We have added this rationale and the detailed state-labeling criteria to the Supplementary.

Accordingly, we revised the terminology throughout the manuscript. State 1 is now referred to as the "*Conditioning-sensitive global circuit activation state*," rather than simply a "*Global threat-circuit activation state*." This label reflects two observations: first, this state showed coordinated positive activation across most regions of the 24-node threat-circuit atlas; second, it was the state showing the most consistent conditioning-related changes across multiple dynamic metrics, including occurrence rate, dwell time, transition probability, and trajectory entropy. We also revised the labels of the other states to more explicitly and rigorously reflect their dominant spatial profiles. State 2 is described as a "*Salience–default mode antagonistic state*," characterized by elevated dAI, PI, and angular activity together with suppression of vmPFC, sgACC, and PCC. State 5 is described as a "*Global circuit deactivation state*," reflecting widespread attenuation across the 24-node threat-circuit atlas.

We revised these states on the main text as following:

> "As shown in figure 2A, these states were distinguished by their spatial activation profiles and were labeled based on their predominant circuit engagement. (1) Conditioning-sensitive global circuit activation state, characterized by coordinated positive activation across the majority of regions and conditioning-related increases across multiple dynamic metrics following fear conditioning; (2) Salience–default mode antagonistic state, marked by elevated dAI, Angular activity with suppression of vmPFC, sgACC, and PCC; (3) Silent state, exhibiting minimal activation across regions; (4) Fronto-motor regulatory state, dominated by MFG, SMA, and PCC activation with concurrent suppression of insular regions and (5) Global circuit deactivation state, reflecting widespread attenuation across the circuit.(Main text, p7, line182)"

We also add new section in supplement as following:

> "To improve interpretability, the five coactivation states were labeled according to their dominant spatial activation profiles. Specifically, state labels were assigned based on the ROIs showing the most prominent positive and negative activation weights, following a commonly adopted approach in CAP- and HMM-based studies in which states are descriptively named according to their most salient regional or network-level activation patterns. The spatial activation profiles of all five states are shown in Figure S3.(Supplement 11, line 299)

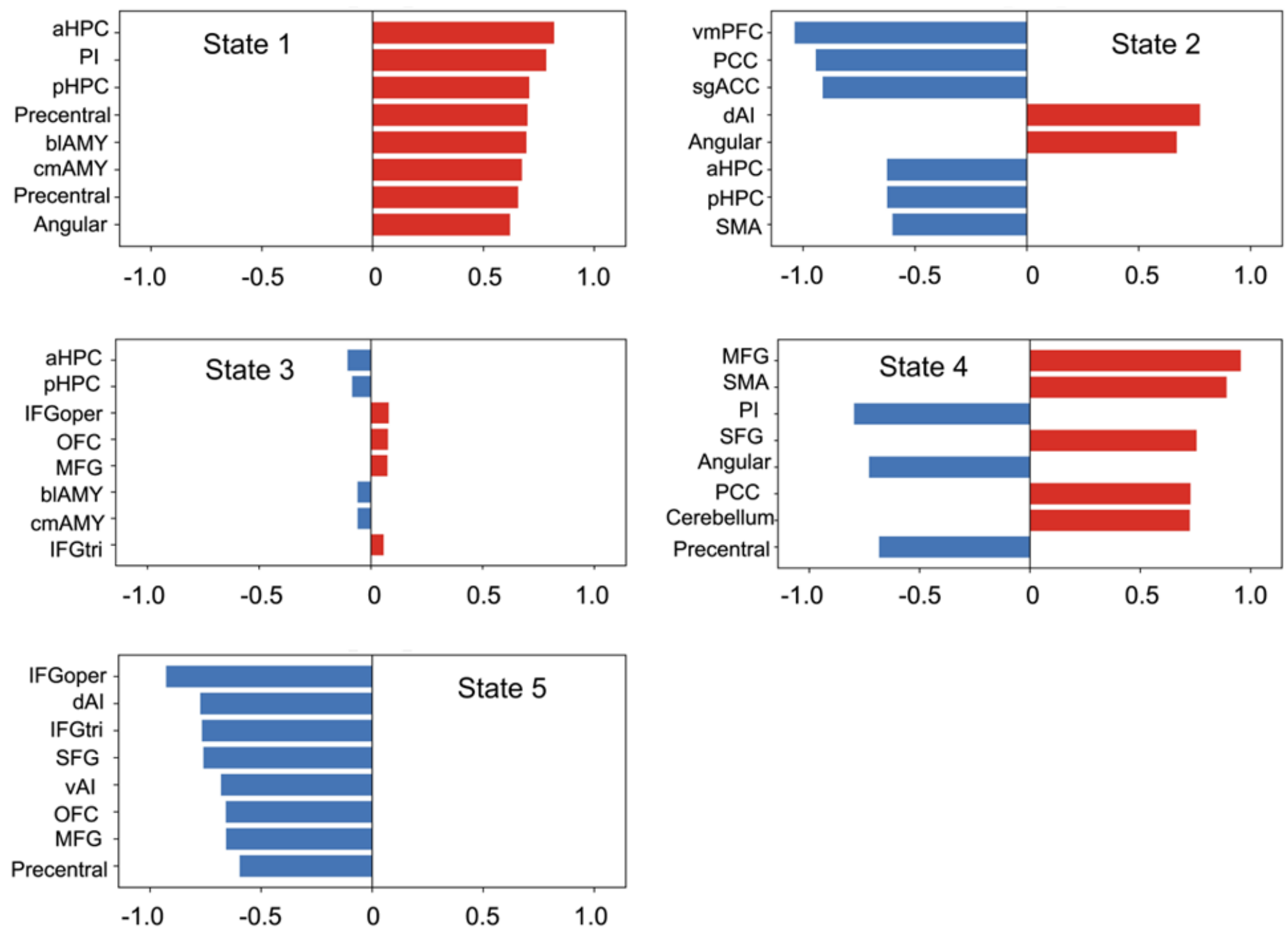

Figure S3. Spatial activation profiles of the five coactivation states. For each state, red bars indicate the ROIs with the strongest positive activation weights and blue bars indicate the ROIs with the strongest negative activation weights"

Comment 19*. TMS Specificity Interpretation (lines 336–339)*
The claim that the absence of prediction in the CS+NoTMS condition "suggests that naturally acquired extinction may engage a convergent, group-level neural implementation" is difficult to follow. What is meant by "convergent, group-level neural implementation," and why would such an implementation preclude individual-difference prediction? This interpretation is not self-evident and requires elaboration. Furthermore, the null result in CS+NoTMS (r = 0.14–0.19, p > 0.05) cannot be straightforwardly interpreted as evidence of a true mechanistic dissociation from the TMS condition. The issue is not the overall sample size, but rather that effect sizes of this magnitude are small enough that non-significance may reflect insufficient sensitivity to detect a weak effect rather than a true absence of one. Without a formal equivalence test specifying the smallest effect size of interest, or a power analysis targeting this specific effect size range, the observed null result in the NoTMS condition does not license the mechanistic conclusion the authors draw from it. The authors should either conduct such an analysis or substantially temper the interpretive claims made on the basis of this comparison.

Response: We agree that our previous interpretation was speculative. Specifically, we acknowledge that the phrase "convergent, group-level neural implementation" was insufficiently defined and that the absence of statistically significant prediction in the

CS+NoTMS condition should not be interpreted as evidence for a true absence of individual-difference effects, nor as definitive support for a mechanistic dissociation from the TMS condition. We also agree that the observed non-significant associations in the CS+NoTMS condition ($r = 0.14–0.19$) may reflect weak effects that were not detected with sufficient statistical confidence, rather than a true absence of predictive coupling.

In response to this concern, we firstly have removed the original mechanistic interpretation and substantially tempered the relevant discussion. Accordingly, we have revised the relevant discussion as follows:

> "Notably, although both CS+TMS and CS+NoTMS conditions exhibited fear extinction, only extinction paired TMS did fear-learning–induced brain reorganization predict later recall and renewal activations. This dissociation may reflect an important property of neuromodulation. (Main text, p17, line 399)"

In addition, we performed an additional direct statistical comparison of predictive performance between the TMS-modulated and natural extinction conditions. Specifically, we compared the cross-validated prediction correlations ($\Delta r = r(TMS) - r(NoTMS)$ using a paired permutation framework based on the same 1,000 permutation distributions used throughout the predictive analyses. This analysis demonstrated that prediction performance was significantly greater under the TMS-modulated extinction condition than under natural extinction for both extinction-memory recall (Δr, permutation $p = .002$) and renewal (Δr, permutation $p = .04$). The result provides direct statistical support for the related statements throughout the manuscript that prediction performance was significantly stronger under TMS-modulated extinction than under natural extinction.

For the concern related to the *TMS Specificity Interpretation*, we tempered our interpretation. Specifically, we now propose that stimulation during extinction may have interacted with the neural configuration established after conditioning, potentially making subsequent memory-related activation more dependent on pre-existing individual differences. We include this possible interpretation in the revised Discussion as follows:

> "...A growing body of literature suggests that focal brain stimulation does not remain restricted to the stimulated cortical site, but propagates through distributed large-scale functional networks. Such perturbations have been shown to vary substantially across individuals, contributing to considerable heterogeneity in both neural and behavioral responses to TMS. Within this framework, TMS may alter the neural implementation of extinction learning relative to natural extinction. Rather than affecting all participants uniformly, stimulation may allow pre-existing individual differences in fear-learning–related brain organization to exert a stronger influence on subsequent memory-expression processes, thereby increasing the detectability of brain–brain predictive relationships. Meanwhile, the absence of significant prediction in the no-TMS condition does not indicate that Day 1 brain organization is unrelated to extinction without stimulation. It only indicates that the

> specific Day 1 neural signature examined here did not reliably predict the corresponding Day 3 activation pattern. (Main text, p17, line35)"

General

***Comment 20**. Canonical Loading Stability*
The manuscript reports canonical loading profiles as a primary interpretive result but provides no assessment of whether the same brain regions consistently drive the model across CV folds. A stability analysis, such as bootstrap confidence intervals or fold-by-fold correlation of loading vectors, is needed to support the neural signature interpretation.

Response: To address this concern, we performed an additional non-parametric bootstrap stability analysis (1000 iterations) for both the connectivity-side and activation-side canonical loading profiles. In each iteration, participants were resampled with replacement and canonical loadings were recomputed. Bootstrap distributions were subsequently used to estimate 95% confidence intervals and evaluate the reproducibility of the identified neural signatures.

Accordingly, we have incorporated a description of this procedure into the revised Methods section in main text as follows:

> "To characterize neural signatures contributing to the predictive models, canonical loadings were computed as the Pearson correlation between each original brain feature (X-side: functional connectivity; Y-side: brain activation) and the corresponding canonical variate derived from the model. For visualization of connectivity-side neural signatures, significant functional connections were identified using false discovery rate (FDR) correction ($q < .05$) across all connectivity features. To evaluate the stability of the identified neural signatures, we additionally performed a non-parametric bootstrap procedure (1000 iterations). In each iteration, participants were resampled with replacement and canonical loadings were recomputed. Bootstrap distributions were then used to estimate confidence intervals and assess the stability of both connectivity-based and activation-based loading profiles across resamples. (Main text, p25, line 639)"

Furthermore, to improve transparency regarding the robustness of the identified neural signatures, we added the following statement to the revised Results section:

> "To evaluate the robustness of these neural signatures, we performed a non-parametric bootstrap stability analysis (1000 iterations) for both connectivity-side and activation-side canonical loading profiles. Across both recall and renewal

models, the dominant connectivity features and regional activation patterns identified in the primary rCCA analyses were consistently reproduced across bootstrap resamples. Additionally, the highest-contributing functional connections and brain regions exhibited stable loading estimates and preserved their relative importance rankings across iterations (More details see supplement). (Main text p13, line 291)"

Detailed bootstrap stability results, including confidence intervals and loading-profile reproducibility analyses for both recall and renewal models, are now provided in the Supplementary Results as follows:

"Stability of Connectivity- and Activation-Side Neural Signatures

To evaluate the robustness of the neural signatures identified by the rCCA models, we performed a non-parametric bootstrap analysis (1000 iterations) and estimated confidence intervals for both connectivity-side and activation-side canonical loading profiles (Figure S8).

For the recall model (Figure S8A), the connectivity-side neural signature showed high stability across bootstrap resamples. The strongest contributing functional connections identified in the original analysis remained among the top-ranked features following resampling, and most major connections retained loading estimates that were consistently different from zero. Similarly, the activation-side neural signature was robust, with the caudate, cerebellum, precentral gyrus, superior frontal gyrus (SFG), posterior cingulate cortex (PCC), and related regions remaining the dominant contributors across bootstrap iterations. The relative ranking of these regions was largely preserved, indicating that the recall-related activation signature was not driven by a small subset of participants.

For the renewal model (Figure S8B), bootstrap analysis likewise demonstrated stable connectivity-side and activation-side loading profiles. Functional connections involving the orbitofrontal cortex (OFC), dorsal anterior insula (dAI), ventral anterior insula (vAI), inferior frontal gyrus (IFG), and related regions consistently exhibited the strongest loading magnitudes across resamples. On the activation side, IFG opercularis, posterior insula (PI), IFG triangularis, precentral gyrus, subgenual ACC (sgACC), and neighboring regions remained the most prominent contributors. Additionally, the ordering of the highest-loading regions and connections closely matched that observed in the original models.

Overall, the bootstrap results demonstrated that both recall- and renewal-related predictive signatures were highly reproducible across resamples. The principal connectivity and activation features identified in the primary analyses consistently remained among the strongest contributors and exhibited stable loading estimates, supporting the robustness and interpretability of the identified brain–brain predictive relationships (Supplement, p17, line 444).

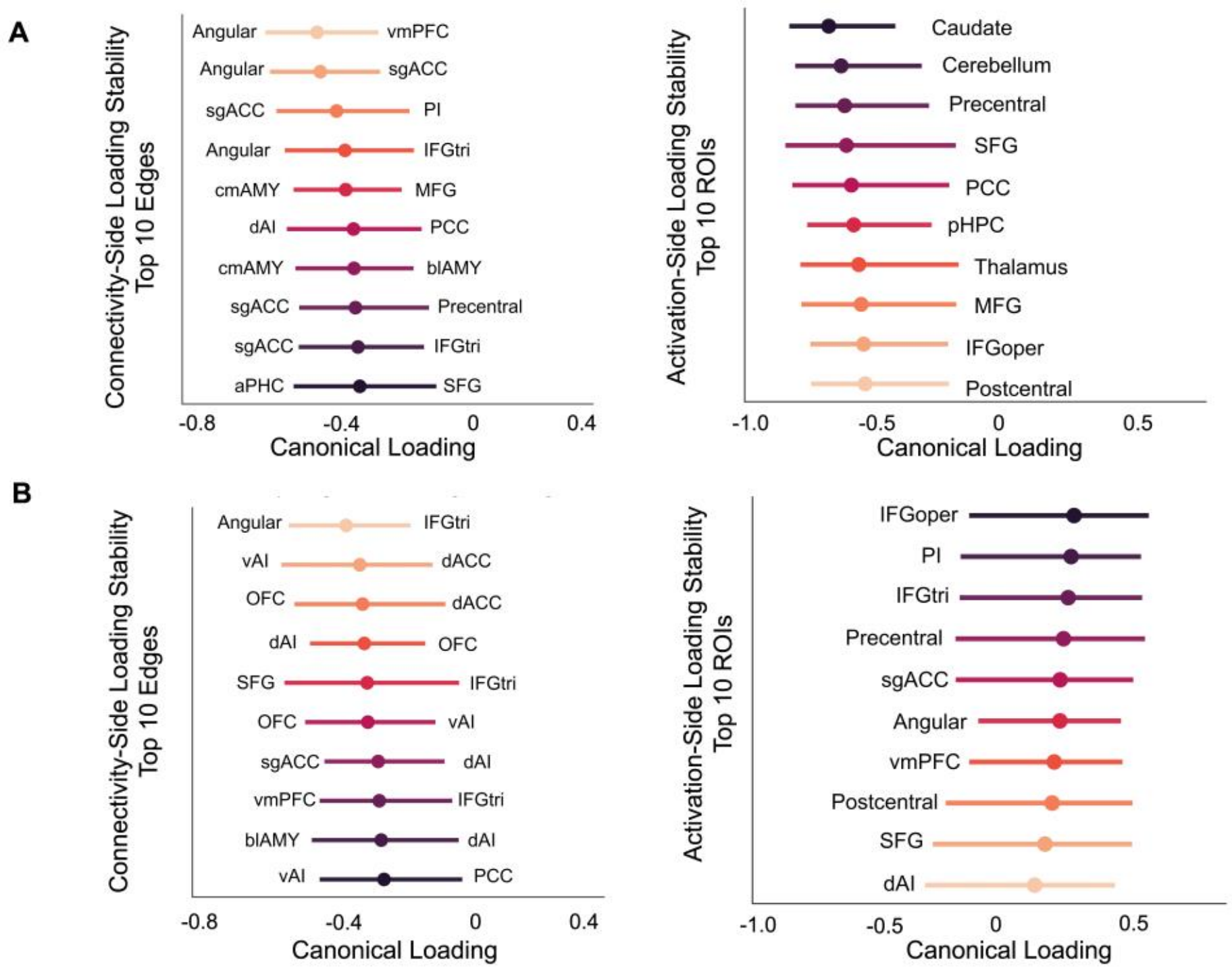

Figure S8. Bootstrap stability of connectivity-side and activation-side neural signatures. (A) Bootstrap stability analysis of the neural signatures identified in the memory recall prediction model. (B) Bootstrap stability analysis of the neural signatures identified in the memory renewal prediction model."

***Comment 21**. Behavioral Measure*
The extinction degree index relies exclusively on subjective fear ratings with no physiological corroboration (e.g., skin conductance response). This should be acknowledged as a limitation of the behavioral assessment.

Response：We thank the reviewer for this valuable comment. In response to this suggestion, we performed additional analyses of skin conductance responses (SCR).

Specifically, we analyzed SCR during the conditioning and memory recall sessions to evaluate **successful fear acquisition** and the **reduction of conditioned responses following extinction**. These physiological findings are consistent with the subjective fear ratings and provide independent evidence supporting the behavioral effects reported in the manuscript.

Accordingly, we have incorporated the SCR analyses into the revised manuscript, specifically:
We have added a description of SCR acquisition to the Methods in main text as follows:

> "Skin conductance responses (SCRs) were also continuously acquired during the conditioning, memory recall, and renewal phases to provide an implicit psychophysiological measure of conditioned fear expression. Detailed SCR acquisition, preprocessing, and statistical analyses are described in the Supplementary Methods. (Main text, p25, line 650)"

We have added a detailed description of SCR preprocessing, quantification, and statistical analyses to the Supplementary Methods as follows:

> "Skin conductance responses (SCRs) were recorded continuously during the conditioning, memory recall, and renewal phases to quantify implicit psychophysiological responses to conditioned stimuli.
>
> For each conditioned-stimulus (CS) presentation, SCR amplitude was quantified as the peak response occurring within 0–6 s following CS onset relative to the mean signal during the 2-s pre-stimulus baseline. Trial labels were determined according to the corresponding event files. During conditioning, trials were categorized as CSm, CSp1, and CSp2. Because both CSp1 and CSp2 represented conditioned threat cues, they were combined as CS+, whereas CSm was treated as CS−.
>
> For each participant, SCR amplitudes were averaged across all presentations of CS+ and CS−, respectively, and these participant-level estimates were compared using paired-sample t-tests to evaluate successful fear acquisition.
>
> To further evaluate extinction-related changes in implicit fear expression, trial-by-trial SCR responses during the Day 3 memory-recall phase were extracted separately for CS+TMS, CS+NoTMS, and CS−. In addition, for each participant, SCR amplitudes were averaged separately across the first four and last four recall trials within each condition, and paired-sample t-tests were performed to assess changes in conditioned autonomic responses across the recall session.(Supplement p8, line 239)"

We also included the SCR analyses and corresponding statistical results in revised supplement as follows:

> "To determine whether the experimental paradigm successfully induced conditioned fear learning and extinction at the implicit psychophysiological level, we examined skin conductance responses (SCRs) during both the conditioning and Day 3 memory-recall phases.
>
> During conditioning, conditioned threat cues (CS+) elicited significantly greater autonomic responses than safety cues (CS−) during early acquisition (Figure S1A). Specifically, participant-level SCR amplitudes averaged across CS+ presentations were significantly greater than those averaged across CS− presentations (CS+: 0.43 ± 0.54; CS−: 0.20 ± 0.23; paired t-test: $t(78) = 5.60$, $p < .001$, Cohen's dz = 0.63), providing psychophysiological evidence for successful fear acquisition. We

additionally performed a sensitivity analysis using square-root transformed SCR amplitudes (negative values were set to zero prior to transformation). The transformed data yielded highly similar results, with significantly greater SCR responses to CS+ than CS− (CS+: 0.560 ± 0.350; CS−: 0.369 ± 0.247; paired t-test: $t(78) = 7.94$, $p < .001$, Cohen's dz = 0.89).

We next examined trial-by-trial SCR dynamics during the Day 3 recall session to characterize the temporal evolution of conditioned autonomic responses during extinction-memory expression (Figure S1B).

Both CS+TMS and CS+NoTMS conditions exhibited progressively decreasing SCR amplitudes across recall trials. Consistent with these trajectories, participant-level SCR amplitudes averaged across the first four recall trials were significantly greater than those averaged across the last four recall trials for both extinction conditions (CS+TMS: $t(78) = 4.08$, $p < .001$, Cohen's dz = 0.46; CS+NoTMS: $t(78) = 4.00$, $p < .001$, Cohen's dz = 0.45); this difference was replicated when using square-root transformed SCR amplitudes (CS+TMS: $t(78) = 4.93$, $p < .001$, Cohen's dz = 0.55; CS+NoTMS: $t(78) = 5.96$, $p < .001$, Cohen's dz = 0.67), indicating that conditioned autonomic responses progressively diminished during memory recall rather than remaining stable until the final subjective assessment.

Above all, these psychophysiological findings provide convergent evidence that the experimental paradigm successfully established conditioned fear acquisition and extinction-memory expression at both the subjective and autonomic levels. (Supplement p9, line 272)

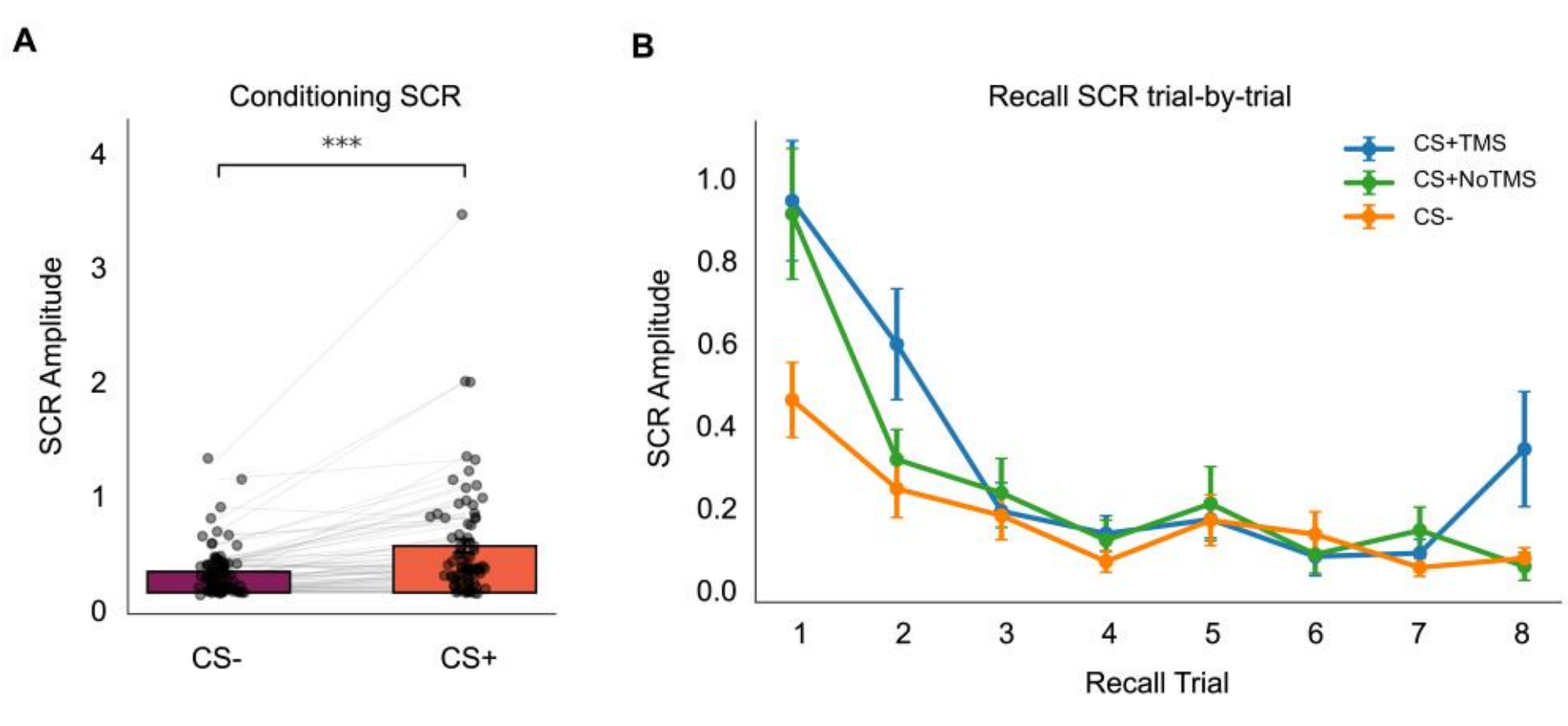

Figure S1. Psychophysiological evidence for successful fear acquisition and extinction. (A). Comparison between CS+ and CS- during fear conditioning. (B). Trial-by-trial psychophysiological dynamics during extinction-memory recall.”

Comment 22. *Overall, the nested cross-validation architecture and permutation-based inference are genuine methodological strengths, and the experimental design is among the more ambitious and carefully considered in the human fear-learning literature.*

However, the conflation of brain reorganization and TMS effects in the abstract, the absence of a clear TMS rationale and hypothesized direction, the unexplained discrepancy between out-of-sample and supplementary correlations, and several instances of imprecise or overstated interpretation all require substantive revision before the findings can be fully evaluated.

Response： We sincerely thank the reviewer for this thoughtful overall assessment and for recognizing the strengths of our experimental design and analytical framework. We also appreciate the reviewer's constructive suggestions regarding the presentation and interpretation of our findings. In the revised manuscript, we have carefully addressed each of these concerns through substantial revisions to the Abstract, Introduction, Methods, Results, Discussion, and figure presentation. We believe that these revisions have substantially strengthened both the scientific rigor and the presentation of our work, and we are grateful for the reviewer's insightful comments that helped improve the manuscript.

Reference

1. Gießing, C., Alavash, M., Herrmann, C. S., Hilgetag, C. C. & Thiel, C. M. Individual differences in local functional brain connectivity affect TMS effects on behavior. *Sci Rep* **10**, 10422 (2020).
2. Nicolo, P., Ptak, R. & Guggisberg, A. G. Variability of behavioural responses to transcranial magnetic stimulation: Origins and predictors. *Neuropsychologia* **74**, 137–144 (2015).
3. Janssens, S. E. W. & Sack, A. T. Spontaneous Fluctuations in Oscillatory Brain State Cause Differences in Transcranial Magnetic Stimulation Effects Within and Between Individuals. *Front. Hum. Neurosci.* **15**, (2021).
4. Milad, M. R. & Quirk, G. J. Neurons in medial prefrontal cortex signal memory for fear extinction. *Nature* **420**, 70–74 (2002).
5. Lebrón, K., Milad, M. R. & Quirk, G. J. Delayed Recall of Fear Extinction in Rats With Lesions of Ventral Medial Prefrontal Cortex. *Learn. Mem.* **11**, 544–548 (2004).
6. Raij, T. *et al.* Prefrontal Cortex Stimulation Enhances Fear Extinction Memory in Humans. *Biol Psychiatry* **84**, 129–137 (2018).
7. Chen, R. *et al.* Depression of motor cortex excitability by low-frequency transcranial magnetic stimulation. *Neurology* **48**, 1398–1403 (1997).
8. Pascual-Leone, A., Valls-Solé, J., Wassermann, E. M. & Hallett, M. Responses to rapid-rate transcranial magnetic stimulation of the human motor cortex. *Brain* **117 (Pt 4)**, 847–858 (1994).
9. Milad, M. R. & Quirk, G. J. Fear extinction as a model for translational neuroscience: ten years of progress. *Annu Rev Psychol* **63**, 129–151 (2012).

10. Olsson, A. & Phelps, E. A. Social learning of fear. *Nat Neurosci* **10**, 1095–1102 (2007).

11. Greco, J. A. & Liberzon, I. Neuroimaging of Fear-Associated Learning. *Neuropsychopharmacol* **41**, 320–334 (2016).

12. Fullana, M. A. *et al.* Neural signatures of human fear conditioning: an updated and extended meta-analysis of fMRI studies. *Mol Psychiatry* **21**, 500–508 (2016).

13. Liu, X. *et al.* Pathological fear, anxiety and negative affect exhibit distinct neurostructural signatures: evidence from psychiatric neuroimaging meta-analysis. *Transl Psychiatry* **12**, 405 (2022).

14. Klavir, O., Prigge, M., Sarel, A., Paz, R. & Yizhar, O. Manipulating fear associations via optogenetic modulation of amygdala inputs to prefrontal cortex. *Nat Neurosci* **20**, 836–844 (2017).

15. Sotres-Bayon, F., Sierra-Mercado, D., Pardilla-Delgado, E. & Quirk, G. J. Gating of Fear in Prelimbic Cortex by Hippocampal and Amygdala Inputs. *Neuron* **76**, 804–812 (2012).

16. Burgos-Robles, A. *et al.* Amygdala inputs to prefrontal cortex guide behavior amid conflicting cues of reward and punishment. *Nat Neurosci* **20**, 824–835 (2017).

17. Burgos-Robles, A., Vidal-Gonzalez, I. & Quirk, G. J. Sustained Conditioned Responses in Prelimbic Prefrontal Neurons Are Correlated with Fear Expression and Extinction Failure. *J. Neurosci.* **29**, 8474–8482 (2009).

18. Phelps, E. A. & LeDoux, J. E. Contributions of the Amygdala to Emotion Processing: From Animal Models to Human Behavior. *Neuron* **48**, 175–187 (2005).

19. Yin, S., Liu, Y., Petro, N. M., Keil, A. & Ding, M. Amygdala Adaptation and Temporal Dynamics of the Salience Network in Conditioned Fear: A Single-Trial fMRI Study. *eNeuro* **5**, ENEURO.0445-17.2018 (2018).

20. Milad, M. R. *et al.* Recall of Fear Extinction in Humans Activates the Ventromedial Prefrontal Cortex and Hippocampus in Concert. *Biological Psychiatry* **62**, 446–454 (2007).

21. Milad, M. R. *et al.* Neurobiological basis of failure to recall extinction memory in posttraumatic stress disorder. *Biol Psychiatry* **66**, 1075–1082 (2009).

22. LeDoux, J. The emotional brain, fear, and the amygdala. *Cell Mol Neurobiol* **23**, 727–738 (2003).

23. Pattwell, S. S. *et al.* Altered fear learning across development in both mouse and human. *Proceedings of the National Academy of Sciences* **109**, 16318–16323 (2012).

24. Wen, Z. *et al.* Temporally and anatomically specific contributions of the human amygdala to threat and safety learning. *Proceedings of the National Academy of Sciences* **119**, e2204066119 (2022).

25. Wen, Z. *et al.* Distributed neural representations of conditioned threat in the human brain. *Nat Commun* **15**, 2231 (2024).

26. Nader, K., Schafe, G. E. & Le Doux, J. E. Fear memories require protein synthesis in the amygdala for reconsolidation after retrieval. *Nature* **406**, 722–726 (2000).

27. Zidda, F. *et al.* Default mode network connectivity of fear- and anxiety-related cue and context conditioning. *NeuroImage* **165**, 190–199 (2018).

28. Schultz, D. H., Balderston, N. L. & Helmstetter, F. J. Resting-state connectivity of the amygdala is altered following Pavlovian fear conditioning. *Front. Hum. Neurosci.* **6**, (2012).

29. Tetereva, A., Kartashov, S., Ivanitsky, A. & Martynova, O. Variance and Scale-Free Properties of Resting-State Blood Oxygenation Level-Dependent Signal After Fear Memory Acquisition and Extinction. *Front. Hum. Neurosci.* **14**, (2020).

30. Feng, P., Feng, T., Chen, Z. & Lei, X. Memory consolidation of fear conditioning: Bi-stable amygdala connectivity with dorsal anterior cingulate and medial prefrontal cortex. *Soc Cogn Affect Neurosci* **9**, 1730–1737 (2014).

31. Tambini, A., Rimmele, U., Phelps, E. A. & Davachi, L. Emotional brain states carry over and enhance future memory formation. *Nat Neurosci* **20**, 271–278 (2017).

32. Feng, P., Becker, B., Zheng, Y. & Feng, T. Sleep deprivation affects fear memory consolidation: bi-stable amygdala connectivity with insula and ventromedial prefrontal cortex. *Soc Cogn Affect Neurosci* **13**, 145–155 (2018).

33. Liu, X., Zhang, N., Chang, C. & Duyn, J. H. Co-activation patterns in resting-state fMRI signals. *NeuroImage* **180**, 485–494 (2018).

34. Xie, L. *et al.* Brain-wide resting-state fMRI network dynamics elicited by activation of single thalamic input. *Nat Commun* **16**, 11247 (2025).

35. Chen, X. *et al.* Leading basic modes of spontaneous activity drive individual functional connectivity organization in the resting human brain. *Commun Biol* **6**, 892 (2023).

36. Liu, Q. *et al.* Energy inefficiency underpinning brain state dysregulation in individuals with major depressive disorder. *Nat. Mental Health* 1–16 (2026) doi:10.1038/s44220-025-00583-4.

37. Sendi, M. S. E. *et al.* Brain dynamics reflecting an intra-network brain state are associated with increased post-traumatic stress symptoms in the early aftermath of trauma. *Nat. Mental Health* **3**, 185–198 (2025).

38. Awada, J. *et al.* A Longitudinal and Reproducible Anti-Coactivation Pattern Between the Cerebellum and the Ventral Tegmental Area Is Related to Apathy in Schizophrenia. *Biological Psychiatry* **99**, 124–133 (2026).

39. Cai, A. *et al.* Unraveling spatiotemporal dynamics in transdiagnosis subtypes of major depressive disorder and bipolar disorder: insights from co-activation patterns and treatment response. *Mol Psychiatry* 1–13 (2025) doi:10.1038/s41380-025-03326-0.

40. Riley, R. D. *et al.* Calculating the sample size required for developing a clinical prediction model. *BMJ* **368**, m441 (2020).

41. Klugah-Brown, B. *et al.* Dynamic brain connectivity patterns induced by oxytocin: An fMRI Co-Activation pattern analysis study. *Mol Psychiatry* 1–13 (2026) doi:10.1038/s41380-026-03549-9.

42. Vergara, V. M., Salman, M., Abrol, A., Espinoza, F. A. & Calhoun, V. D. Determining the number of states in dynamic functional connectivity using cluster validity indexes. *Journal of Neuroscience Methods* **337**, 108651 (2020).

43. Zhang, L., Pini, L., Shulman, G. L. & Corbetta, M. Brain-wide dynamic coactivation states code for hand movements in the resting state. *Proceedings of the National Academy of Sciences* **122**, e2415508122 (2025).

44. Ye, J. *et al.* Altered Brain Dynamics Across Bipolar Disorder and Schizophrenia During Rest and Task Switching Revealed by Overlapping Brain States. *Biological Psychiatry* **94**, 580–590 (2023).

45. Wang, D. *et al.* Altered temporal, but intact spatial, features of transient network dynamics in psychosis. *Mol Psychiatry* **26**, 2493–2503 (2021).
